# Drivers of banana re-infection in newly planted gardens in banana bunchy top disease endemic zones: Implications for the recovery of banana production

**DOI:** 10.1371/journal.pone.0357834

**Published:** 2026-09-11

**Authors:** Bonaventure Aman Omondi, Delano Ronald Togbé, Renata Retkute, Innocent Nduwimana, Celestin Niyongere, Charles Staver

**Affiliations:** 1 Bioversity International, Alliance of Bioversity International and CIAT, c/o IPGRI Building, IITA Campus, Abomey-Calavi, Cotonou, Benin; 2 Faculty of Agronomic Sciences, University of Abomey-Calavi, Abomey-Calavi, Benin; 3 Epidemiology and Modeling Group, Department of Plant Sciences, University of Cambridge, Cambridge, United Kingdom; 4 Bioversity International, The Alliance of Bioversity International and CIAT, co/ IITA Building, Bujumbura, Burundi; 5 Institute of Agronomic Sciences (ISABU) Rohero 1, Avenue de la Cathédrale, Bujumbura, Burundi; 6 CAB International (CABI), Quartier Kabondo Ouest, Avenue du Large, Bujumbura, Burundi; 7 Bioversity International, Parc Scientifique Agropolis 1990 bd Lironde, Montferrier sur Lez, Montpellier, France; 8 Faculty of Agricultural Sciences, Universidad Veracruzana, Xalapa, Mexico; Manonmaniam Sundaranar University, INDIA

## Abstract

Understanding how crop pests and diseases spread is essential for designing effective integrated pest and disease management strategies. This study investigated the landscape, field design, and management factors associated with the persistence and re-infection of Banana Bunchy Top Disease (BBTD) in endemic smallholder systems. We surveyed 121 banana gardens that had been re-established with virus-free tissue-culture plantlets three years earlier to identify the combination of conditions linked to lower disease incidence, to identify candidate parameters to test in controlled evaluation of disease management. BBTD prevalence was positively associated with higher banana density within a 60 m radius of the field, increased aphid abundance, greater varietal diversity and the use of locally sourced planting material. In contrast, higher altitude, the presence of hedgerows and frequent roguing of symptomatic plants were negatively associated with disease incidence. Model averaging showed that aphid prevalence, surrounding banana density, reported roguing, and seed source appeared in more than half of the best-performing models, highlighting their possible role in reducing re-infection and potential yield losses. These findings underscore the importance of coordinated, community-based replanting with certified virus-free planting material, consistent roguing guided by farmer symptom recognition, and the strategic use of barrier hedges or mixed cropping systems to limit aphid dispersal. Further research should explore optimal species combinations and landscape configurations to strengthen long-term BBTD suppression in smallholder production systems.

## 1 Introduction

Invasive species are a major threat to agricultural productivity and the global stability of ecosystems. Their proliferation, often driven by global trade, climate change, and human-mediated landscape alterations, disrupts local ecological balances and exacerbates crop loss [1,2]. In agroecosystems, invasive pests and pathogens can spread rapidly, especially in landscapes with conducive habitats and susceptible hosts in the absence of natural enemies and without coordinated management. Many pests and diseases rapidly establish in new areas of incursion depending on host availability, dispersal mechanisms (including effective vectors), and spatial connectivity between suitable habitats across the landscape. Understanding how host distribution and landscape connectivity influence pest and disease dynamics is important for managing invasive plant pathogens and insect vectors which have become endemic. At the local scale, the spread of disease is influenced by field-level management practices, such as sanitation, crop diversity, and planting patterns, which affect the inoculum pressure and vector behaviour. Spatial modelling of these agro-environmental factors can help to identify ecological thresholds and intervention points for disease control.

*Musa* spp. (banana and plantain) is a vital staple and cash crop contributing to food security and income for millions of smallholder farmers [3]. As a perennial crop, banana supports year-round harvesting, which is especially important for rural households’ food and economic security. More than 50 types and cultivars of banana (including plantain, *Musa* spp.) are cultivated [4–8]. The banana fruit is consumed in multiple forms depending on the variety. Dessert bananas are eaten fresh as fruits, whereas cooking bananas and plantains as starchy staples or snacks consumed boiled or fried. Some varieties (mainly of east African Highland bananas) are used to produce processed beverages, such as banana beer and juice, while others are considered multipurpose because of their suitability for several uses. Banana leaves are used to steam bananas and protein-rich foods, adding flavour during cooking. This wide range of uses makes banana an essential crop for food security, nutrition, and income generation, especially in smallholder farming systems across tropical regions. However, yields across Africa remain among the lowest globally, primarily due to a range of biotic stressors, including pests and diseases often transmitted in locally sourced seed and low land productivity with few external inputs [9]. The case of banana landscapes in Burundi illustrates the challenges of invasive diseases. Farm communities in the Rift Valley lost fields of the previously dominant beer banana variety, ‘Kayinja’, to Fusarium wilt race 1 (Blomme et al., 2011; [10]. They replaced ‘Kayinja’ with ‘Yangambi Km5’ only to face the proliferation of Banana Bunchy Top Disease (BBTD) [11] which is the subject of this paper. Another Fusarium strain, Tropical Race 4 (TR4), has reached sub-Saharan Africa but is not yet widespread. Many cultivars across the region are susceptible to TR4 [12]. Sustainable management of these invasive species is therefore essential for securing food systems and livelihoods on the continent, especially among smallholder farmers.

Banana bunchy top disease, caused by the banana bunchy top virus (BBTV), is one of the most destructive viral diseases affecting bananas [13,14]. It is transmitted by the banana aphid (*Pentalonia nigronervosa*) and infected planting material. Once established, the disease causes a progressive yield loss. More seriously, the local supply of clean banana suckers diminishes as, progressively, more banana mats become infected by BBTV. Farmers lose access to BBTV-free planting material as local seed supply degenerates [9] and banana production collapses. Currently, BBTD has spread to at least 18 countries in Africa and is estimated to affect 6–12 million smallholder households [15,16]. In heavily affected regions such as Malawi and the Democratic Republic of the Congo (DRC), production losses have significantly undermined livelihoods [15]. Moreover, BBTD poses a serious threat to banana genetic diversity in two centres of secondary diversity of cultivated bananas: The East African Highlands (home to over 100 AAA-EA types) [4,6] and West Africa, the centre of plantain diversity [5,8]. Such genetic loss may threaten the long-term sources of genetic material for banana improvement against other emergent biotic and abiotic challenges. Smallholder production systems, dominated by local landraces, are particularly vulnerable because of their dependence on locally sourced, uncertified, informal seed sourcing and the perennial low-input nature of the production system [11]. Smallholder production system involves limited investment in crop monitoring and management after establishment, and so infectious plant parts could remain active in the landscape for long. This is in contrast with high input systems which use clean seed, regular crop monitoring and with complete replanting every few cycles (e.g., 5 years); giving an opportunity eliminate potentially infected mats before re-establishment.

In 2014, The Learning Alliance for BBTD control in Africa (https://www.bbtvalliance.org/), an alliance of international agricultural research centres, sub-Saharan national research organizations, and rural communities undertook banana production recovery efforts in BBTD-affected landscapes in Burundi and eight other countries using a community-based collaborative learning and action approach [17,18]. This strategy involved landscape-scale destruction of potentially infected plants, enforcement of a banana-free fallow period to eliminate inoculum sources and viruliferous aphids, multiplication of disease-free tissue culture planting material, replanting in well-isolated fields, and collaborative monitoring of replanted gardens [19,20]. Effective implementation requires strong landscape-level coordination, particularly in crop monitoring, inoculum reduction by consistent roguing, and the adoption of clean seed supply systems (Omondi et al, 2021).

Understanding the risk of reinfection in recovered banana gardens affected by BBTD is an important component of long-term disease management and the recovery of banana production in BBTD-affected landscapes. Maintaining a low and stable reinfection risk allows for the gradual expansion of recovered areas, utilizing existing buffer zones and facilitating the growth of seed production and exchange systems within these zones. The recovery strategy deployed in Burundi offered a case study to monitor reinfection in 121 small farms previously reestablished with clean planting materials from tissue culture, hardened in two community nurseries in the villages. Each field was evaluated for landscape and cropping factors around it. The study area was characterized by small mixed crop perennial banana gardens and backyard banana mats. At a distance, these landscapes resemble large plantations in terms of banana canopy continuity, but they are a complex mosaic of diverse banana varieties, associated crops, and landscape features such as hedgerows, fallow fields, rice monocrops, and farming practices across an altitudinal gradient.

Our study aimed to identify the key risk factors associated with BBTD reinfection in replanted banana fields to improve the current banana recovery strategies. By assessing the prevalence of BBTD in farms that were replanted with BBTV-free tissue culture planting materials in 2013 and 2014, we aimed to evaluate the factors associated with disease transmission. A single assessment of the reinfection status of initially clean plots across farms with similar background risk provided a unique opportunity to evaluate the risk pressures of BBTD prevalence while minimizing the influence of temporal variability.

## 2 Materials and methods

### 2.1 Study area and farm selection

A field study was conducted on 121 farms across four communes—Kagazi, Rusagara, Munyika, and Gitebe—in Cibitoke Province, Burundi. Cibitoke Province is a major banana-growing region and a known hotspot for Banana Bunchy Top Disease (BBTD). It lies along the border between Burundi, Rwanda, and the Democratic Republic of Congo (DRC), spanning altitudes from 800 to 1300 m above sea level. Rusizi Valley, shared among these three countries, has been endemic to BBTD for over three decades. The spread of BBTV through the vector is favoured by environmental factors favouring vector aphid population build up and migration, and rapid plant growth and accumulation of BBTV titre to enable vector acquisition. These include favourable temperature, wind and topography [21]. Thus, the greatest BBTD risk has been associated with humid lowlands. Pilot sites were established in selected communes in 2014 to evaluate community-based approaches to BBTD recovery [19,22]. The farms assessed in this study had been replanted using BBTV-free tissue culture (TC) plantlets sourced from a private tissue culture propagation laboratory (Agrobiotec Limited, Bujumbura, Burundi) and hardened in community-based nurseries. While farmers were trained and encouraged to implement BBTD risk reduction and cultural management practices, implementation varied based on individual capacities and priorities. As all farms shared a known replanting start date with virus-free material, they provided a consistent baseline for assessing the environmental and management factors influencing BBTD reinfection. The presence of BBTD in the surrounding landscape ensured a comparable source of inoculum across sites, minimizing spatial bias that would otherwise occur in newly affected areas where infections remain localized in hotspots. By focusing on replanted fields, we eliminated the confounding effects of local disease history and topographic variation, allowing plot-level history and active management practices to emerge as the main explanatory factors.

### 2.2 Ethical statement

This study was conducted as part of a voluntary collaboration with the National Agricultural Research Institute (ISABU) and involved structured interviews with farmers on crop management practices. As the research focused solely on non-sensitive agricultural practices and did not involve biomedical procedures, vulnerable populations, or collection of sensitive personal data, formal ethical clearance was not required under applicable national and institutional guidelines for routine agricultural research. Participation was entirely voluntary; the study objectives were explained to all participants, informed verbal consent was obtained prior to interviews, and respondents were assured of confidentiality, anonymity in reporting, and their right to withdraw at any time without consequence. No personally identifiable information is included in this publication.

### 2.3 Data collection

**Fig 1:**
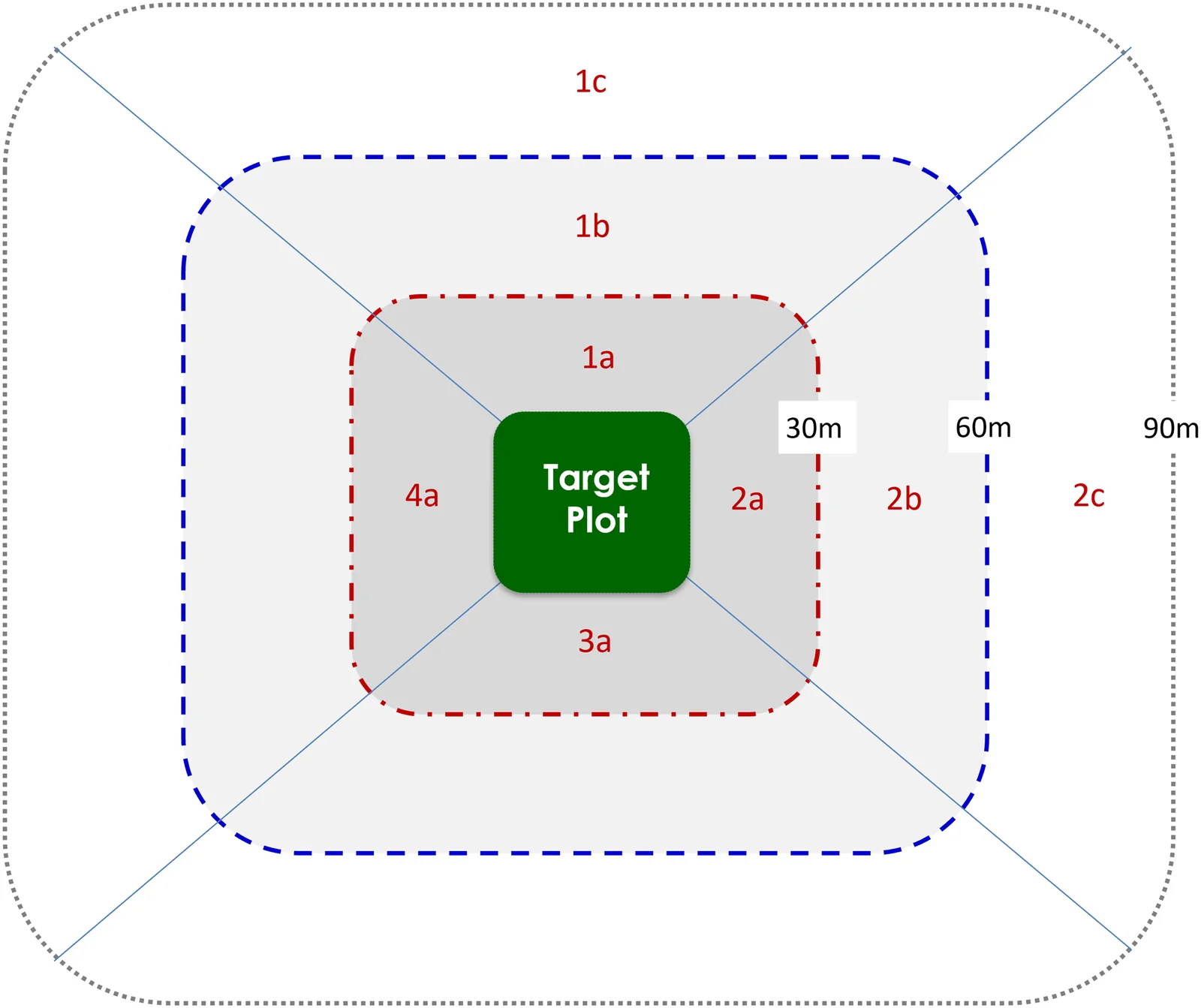
Sketch of the plot assessment plan showing the target plot (green) and the areas assessed for vegetation characterization.

The data were collected using a combination of direct field measurements and structured surveys. A sketch map of each target garden was created to orient the characterization and data acquisition of the field itself and a 90m surrounding observational zone corresponding to the buffer proposed to reduce aphid movement to the field. Thus, each of the 121 gardens planted with tissue culture (TC) banana plantlets in the BBTD recovery effort three months prior, was treated as a focal plot. The buffer was divided into three concentric bands of 30m each, following the observation that aphid arrival to a field declined with distance from the field up to 90 m, when the likelihood was very low (Fig 1; [23], The data collection team underwent standardized training using a uniform disease classification protocol, implemented cross-auditing of observations between data collectors, and leveraged experienced personnel previously trained and validated in accurate BBTD identification across multiple countries.

Each of the 121 replanted plots was assessed for crop density and diversity within the replanted gardens, banana cultivar diversity, and BBTD incidence. In each target field, two diagonal transect lines were laid along which plants were sampled at regular intervals to cover all quarters of the plot. Every third plant was assessed to determine BBTD incidence and aphid presence, along two X-transects, to cover all quarters of the garden. BBTD incidence was recorded by visually counting the symptomatic plants, which served as the primary response variable [24]. BBTV is a systemic virus, once a single shoot is infected, the virus spreads to all other shoots in the mat [25]. The unit of assessment was the banana mat, which included all shoots connected via the rhizome. The three youngest fully opened leaves per plant/shoot and three shoots of different sizes per mat were examined for BBTD symptoms on the lamina, petiole, and midrib following established protocols [19–21]. A mat was marked as diseased if any of its shoots exhibited any symptoms. The proportion of symptomatic mats was used as a proxy for the proportion of BBTV-infected plants.

Banana aphid populations were assessed in a single field evaluation conducted on each selected sampling plot at the time of data collection. Within each plot, three shoots (small, medium, and large) were inspected within each of the selected banana mat. For each shoot, the cigar leaf, top unfurled leaf and outermost leaf sheaths were carefully opened and examined for the presence of banana aphid (*Pentalonia nigronervosa*) colonies [26]. A modified version of Magee’s [27] banana aphid scale was used to classify colony development and reproductive complexity on a semi-quantitative 0–3 scale (Table 1). Aphid colonies were scored as follows: 0 – no aphids; 1 – solitary aphids or simple colonies comprising one apterous adult with neonates; 2 – developing colonies with several adults and multiple nymphs; and 3 – large, complex colonies containing apterous and alate adults with nymphs. This highest category reflected the greatest dispersal potential and epidemiological importance for BBTV transmission. The adapted scale therefore provided a consistent measure of colony complexity while retaining the biological basis of Magee’s original classification. A binomial scale was also used to express aphid presence per plant, scored as **0** = no aphids seen and **1** = colonies present. These data were separated for symptomatic and asymptomatic mats in the target plot and the buffer areas assessed.

**Table 1:** Complete list of variables and codification used before variable selection^1^.

Code	Variables	Range	Levels
PSm2	Plot size in m²	4 - 15624	null
DNIM	Distance of Nearest BBTD infected banana mat (meters)	0.275 - 197	null
BD30m	Mean of Banana density 30m (Mats/m²)	0.002–0.378	null
BD60m	Mean of Banana density 60m (#Mats/m²)	0.002–0.180	null
BD90m	Mean of Banana density 90m (#Mats/m²)	0.002–5.501	null
RPY	Roguing per year farmer declaration	0–72	null
BBTD^1^	Number of plants affected by BBTD	1 - 57	null
AAP	Aphids’ presence on asymptomatic mother plant	null	Yes (1), No (0)
ASP	Aphids’ presence on symptomatic mother plants	null	Yes (1), No (0)
CSAM	Size of colony on Asymptomatic mats	null	Small simple colony, Large simple colonies (Mixed colonies (apterous and alate))
EpE	Edge presence East	null	Yes (1), No (0)
EpS	Edge presence South	null	Yes (1), No (0)
EpW	Edge presence West	null	Yes (1), No (0)
EpN	Edge presence North	null	Yes (1), No (0)
SeedS	Seed sources	null	1-Did not receive other **seed**s, 2-received other seeds
Var	Varieties	null	one variety (1), two varieties (2), three varieties (3), four varieties (4), more than 5 varieties (5)
OG	Plot owner gender	null	Male (1), female (2)
OPM	Other planting material on the plot	null	Yes (1), No (0)
CC	Associated crop cycle	null	Perennial (1), Seasonal (2), both (3)
MFN	Never planting material received from neighbours’ or unknown sources	null	Yes (1), No (0)
MT	Type of planting material	null	Suckers (1), TC (2), Suckers + TC (3)
SF	Frequency of scouting banana infected	null	Less than monthly (1); Once a month (2); Twice a month (3); Weekly (4); Twice a week (5); Thrice a week (6); Four times a week (7); > four times a week (8)
BFP	Banana-free period	null	Yes (1), No (0)
Loc	Location	null	Munyika (1), Rusagara (2), Mugina (3), Rugombo (4)

^1^See also Supplementary Table 1.

^1^Dependent variable.

For each plot, management data were collected by interviewing plot owners were on the field history, management practices during replanting, cultivar selection, source of planting material, and interaction with neighbouring farms. Social factors (gender of the principal plot owner and farm size) and management practices (varietal diversity, seed sourcing, roguing frequency, use of banana-free fallow, and buffer zone maintenance) were assessed. The roguing protocol recommended for the area involved the immediate cutting of all shoots of a diseased mat at ground level, immediate burial of the shoot pieces under the soil; uprooting the entire corm and exposing it to the sun, chopped in small pieces to avoid sprouting [20]. This addressed two risks of aphid dispersal from the shoot and resprouting of infected rhizomes. Only the diseases mat was removed and farmers were encouraged to scout every month [19]. Self-reported roguing however captured what farmers claimed they did without a parallel evaluation of the actual roguing activity. As this represented two years of activity, we assume the actual practice was relatively close to the recommended due to training and group follow up.

A 90-meter buffer area was laid out around the target plot, divided into three concentric bands of 30 m each, making three bands of 0 to 30m; 30-60m; 60 – 90m. This area represented buffer areas of different vector migration distances from the mother gardens [20]. Each band was scouted for banana and BBTD presence. The immediate boundary line was also scored for the presence or not of a defined hedge/vegetation barrier to vector movement.

Data were collected within both the focal plot and its buffer to evaluate the host distribution (banana density, crop composition, and vegetation diversity) and location characteristics (altitude and topography). A summary of all the variables assessed is presented in Table 1.

### 2.4 Data exploration

The dataset contained missing values (up to 30% overall but dependent on the variable, Supplementary Table 1) that were imputed using the multiple imputation by chained equations (MICE) method from the *mice()* package for categorical variables and medians for continuous variables to enable analysis [28]. To assess potential bias from imputation, we examined the standard errors of the imputed values. The dependent variable of interest was the reported number of mats with BBTD symptoms per plot, with each representing an infection event. Accordingly, count regression (Poisson and negative binomial) models were the most appropriate for assessing the statistically significant factors associated with banana BBTV infection risk. To select the best count model, the mean and variance of the number of diseased banana plants were used to test the overdispersion of count variables. This was followed by an analysis of the number of diseased banana plants per plot.

**Fig 2:**
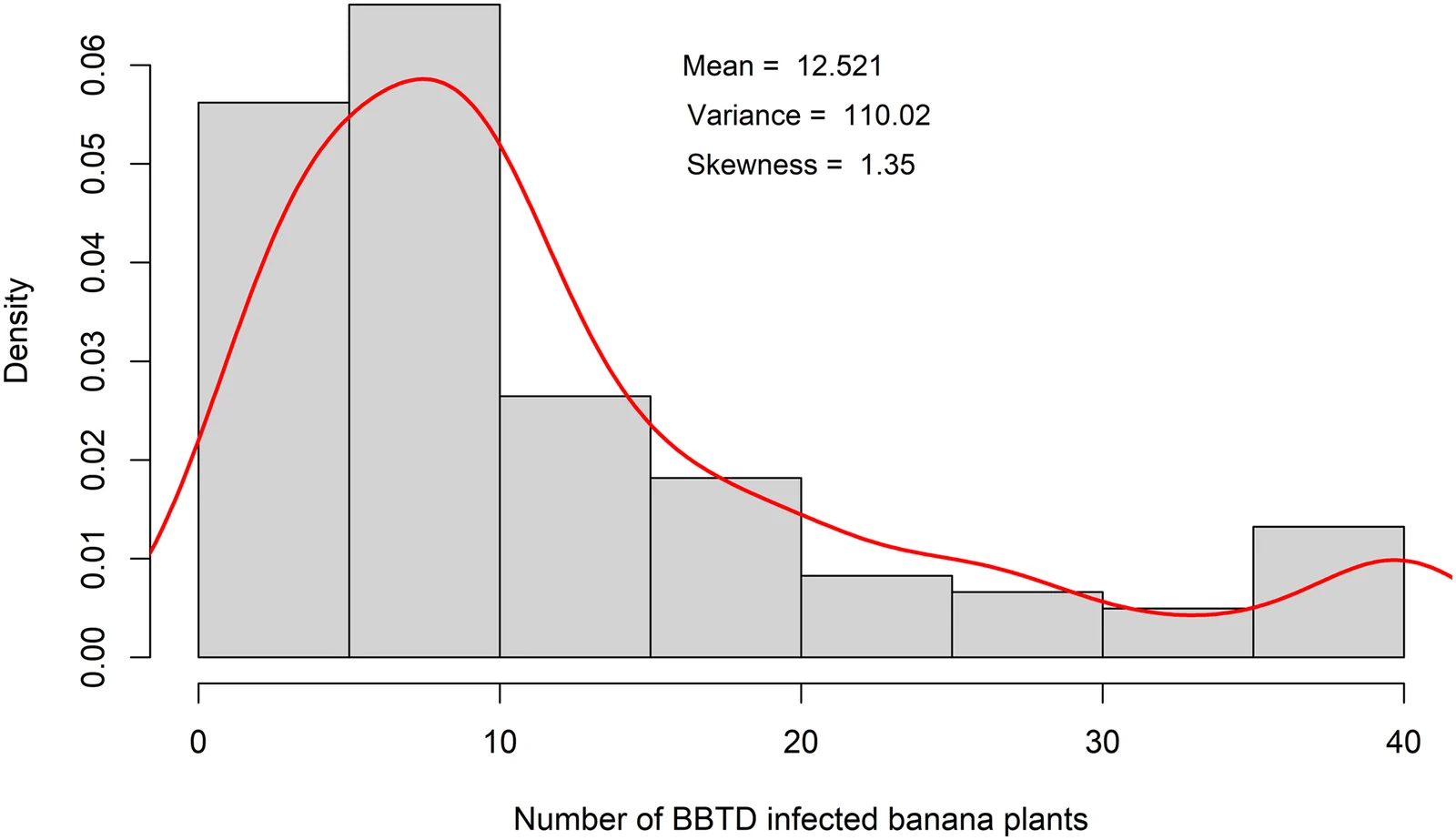
Histogram showing the density of the number of BBTD infected banana plants (for the count regression model).


The response variable showed a positively skewed distribution (skewness = 1.35) and extremely different values for the unconditional mean (12.52) and variance (110.02), which had a nonlinear relationship, suggesting an overdispersion (Fig 2). The log-likelihood ratio test for the significance of overdispersion is computed as follows:

$$\begin{array}{c} Log-likelihoodratio=-\text{2}\times (log-likelihoodofPoissonregression-log-likelihoodofnegativebinomialregression) \end{array}$$

The value of the likelihood-ratio test showed that the data were significantly over-dispersed (χ2 = 256.72, p < 0.0001), thus favouring the negative binomial regression model (NBRM), a generalization of the Poisson model. The NBRM permits sufficient flexibility with only two parameters (the shape parameter and mean). It subsumes the Poisson distribution, while allowing for overdispersion, where the variance may be greater than the mean.

To assess whether infection counts exhibited landscape-level spatial dependence among surveyed sites, we evaluated spatial autocorrelation prior to model fitting. Because regression analyses were conducted at the site level (i.e., field-level aggregated infection counts), the objective was to test for spatial dependence between geographically distinct survey locations rather than within-field clustering driven by short-range vector dispersal. Spatial autocorrelation was evaluated using Global Moran’s I and Local Indicators of Spatial Association (LISA) following established spatial statistical frameworks [29,30]. All analyses were conducted in R using the ***spdep*** package [31]. A spatial weights matrix was constructed using a fixed-distance band approach implemented via *dnearneigh().* Neighbouring sites were defined as those located within 10 km of each other. This generated a binary distance-based neighbour structure, which was converted into a row-standardized spatial weights matrix using *nb2listw(style = “W”)*, such that weights for each site summed to one. The 10 km threshold was selected to ensure adequate neighbour connectivity across the spatially distributed survey network and to evaluate potential landscape-scale dependence. It does not represent aphid dispersal distance, which operates at a much finer spatial scale than the distances separating sampled fields. Global Moran’s I was computed using *moran.test(),* and Local Moran’s I statistics were calculated using *localmoran()* based on the derived spatial weights matrix. The Global Moran’s I statistic for infected plant counts was −0.0212 (standard deviate = −0.588, p = 0.722), indicating no significant global spatial autocorrelation. Local Moran’s I statistics similarly showed no statistically significant clustering or spatial outliers at conventional significance thresholds.

To assess robustness to neighbourhood specification, the analysis was repeated using alternative fixed-distance thresholds of 15 km and 20 km. Results remained non-significant at both distances (15 km: Moran’s I = −0.0138, Z = −0.256, p = 0.601; 20 km: Moran’s I = −0.0178, Z = −0.566, p = 0.714), with statistics consistently close to zero. These findings confirm that the absence of spatial autocorrelation is not sensitive to the choice of distance band and is robust across plausible landscape-scale neighbourhood definitions. These results indicate an absence of meaningful landscape-level spatial autocorrelation among surveyed sites. Consequently, spatial autoregressive or conditional-autoregressive model structures were not incorporated into the regression framework. Disease counts were therefore modeled using negative binomial regression to account for overdispersion, with multicollinearity assessed via variance inflation factors (VIFs), and model selection conducted using AIC-based multi-model inference.

### 2.5 Model fitting

To model the factors affecting the number of plants affected by BBTD, a negative binomial generalized linear model (NB-GLM) was used to fit the global model, which included all the variables (Table 1), followed by model selection and model averaging. The global model was constructed to include all hypothesized predictors, ensuring the exploration of a comprehensive model space.

### 2.6 Multicollinearity checking

To ensure the stability and interpretability of the regression model, potential linear dependencies between the predictor variables in the global model were assessed using the *alias()* function from the *stats* package (version 4.3.2) in R version 4.4.3 ([32]. This feature was used to identify the precise linear relationships within the design matrix. It detects predictors that can be expressed as exact linear combinations of others, thereby indicating multicollinearity problems. Identifying such dependencies ensures that the model parameters are clearly identifiable and interpretable, thereby avoiding estimation problems or misleading conclusions.

Variance inflation factors (VIF) were then calculated using the *vif()* function from the package *car* (version 3.1−2) to assess multicollinearity between the predictors. This function provides generalized variance inflation factor (GVIF) values along with degrees of freedom (df) and adjusted generalized variance inflation factor ${GVIF}^{\left(\frac{1}{2\times Df}\right)}$ values for each variable. Adjustment was performed to make the GVIF values comparable across the variables with different degrees of freedom. Indeed, for some variables, the associated degrees of freedom are greater than one, indicating that they are categorical variables with multiple levels. Variables with high (>5) adjusted GVIFs were discarded to ensure that there was no serious multicollinearity that could affect the stability of regression estimates [33]. Table 2 summarizes the variables retained after the multicollinearity check.

**Table 2:** List of variables included in the analysis after the collinearity test.

Variables.	GVIF	Df	GVIF^(1/(2*Df))
PSm2	3.12	1	1.76
DNIM	1.47	1	1.21
BD30m	2.44	1	1.56
BD60m	2.08	1	1.44
BD90m	1.19	1	1.09
RPY	1.84	1	1.36
AAP	1.43	1	1.19
ASP	1.34	1	1.16
CSAM	3.29	2	1.35
EpE	1.91	1	1.38
EpS	2.15	1	1.47
EpW	2.63	1	1.62
EpN	1.93	1	1.39
SeedS	1.66	1	1.29
Var	7.19	4	1.28
CC	1.90	2	1.17
MFN	1.52	1	1.23
MT	3.01	2	1.32
SF	10.65	7	1.18
BFP	1.44	1	1.20
Loc	6.67	3	1.37

GVIF: Generalized variance inflation factor; GVIF^(1/(2*Df)): Adjusted generalized variance inflation factor; Df: degrees of freedom.

Variable names: **AAP** – Aphids’ presence on asymptomatic mother plant, **ASP** – Aphids’ presence on symptomatic mother plants, **BD30m** - Mean of Banana density 30m (#Mats/m²) **BD60m** - Mean of Banana density 60m (#Mats/m²) **EpE** – Edge presence South; **EpS** – Edge presence South; **EpW**, Edge presence West; **Loc** - Location/Commune/Village; **MFN** – Never planting material received or collected from neighbours unknown; **MT** – Type of planting material; **PSm2** (Plot size in m²); **RPY** – roguing per year farmer declaration; **SeedS** – Seed sources.

### 2.7 Model selection and averaging: assessment of best combinations of descriptors

Effective disease management in agricultural systems is typically achieved through a combination of crop management strategies, which vary depending on the knowledge, experience, and resource availability of individual farmers. Understanding how different management practices interact to influence disease dynamics is essential to develop effective and scalable interventions. We aimed to simulate a hypothetical integrated management regime by identifying combinations of explanatory variables that best accounted for the observed variation in BBTD levels. We employed an information-theoretic approach to model selection by comparing all possible subsets of predictor variables derived from a global model using the corrected Akaike Information Criterion (AICc) [34]. This approach allows for the identification of the most parsimonious models that adequately explain the variation in the response variable while penalizing the model complexity, particularly when working with relatively small sample sizes. Model selection was implemented using the *dredge()* function of the *MuMIn* R package (version 1.47.5), which automated the process of fitting and ranking all potential subset models. This function evaluates each candidate model by computing its AICc score, thus facilitating a comprehensive and unbiased assessment of variable importance. Therefore, we systematically explored the influence of different combinations of management practices on BBTD infection, ultimately guiding the identification of the most promising strategies for integrated disease control.

The Akaike Information Criterion (AIC) is a measure of model quality relative to other models, balancing model fit and complexity. The formula for AIC is:


$$\begin{array}{c} AIC=-2lo:L+2k \end{array}$$

Where:

L = likelihood of data given for each fitted model

k= number of estimated parameters, including intercept.

For small sample sizes, AICc adjusts for potential overfitting by adding a correction term as follows:


$$\begin{array}{c} AICc=AIC+\frac{2k\left(k+1\right)}{n-k-1} \end{array}$$

Where:

n = sample size, and k is defined above.

This adjustment becomes significant when n is small relative to k. By penalizing models as the number of parameters approaches the sample size, AICc ensures a more conservative selection that protects against overfitting [35].

Parallel computing was utilized to enhance computational efficiency during model selection and validation. The *dredge()* function is parallelized across multiple cluster workers, allowing faster exploration of the model space. The candidate models generated by the *dredge()* function were ranked according to their AICc values, with the model exhibiting the lowest AICc considered the best-supported model. Model averaging was then performed using *avg()* function to account for model uncertainty. Models within the two AICc units of the best model were considered to have substantial support. This allowed for robust model comparison while balancing parsimony and predictive accuracy [36]. The subset of models for averaging was selected by computing, for each model, ΔAICc which is the difference in AICc with respect to the AICc of the best candidate model.


$$\begin{array}{c} \Delta AICc={AICc}_{i}-{AIC}_{min} \end{array}$$

Using a subset of models with ΔAICc < 2, the average coefficients and their standard errors were calculated. The ΔAICc values were also used to calculate the Akaike weight $w_{i}$ of each model and the relative importance of the predictor variables appearing in the final model, using the following formula:


$$\begin{array}{c} w_{i}=\frac{exp\left(-\frac{1}{2}{\Delta AICc}_{i}\right)}{\sum_{r=1}^{R}exp\left(-\frac{1}{2}{\Delta AICc}_{r}\right)} \end{array}$$

where R is the number of models and r is the model being considered.

Note that if one of the models must be the best model, then $w_{i}$ is the weight of evidence that a model is in fact the best model for the data and that all the models in the candidate subset have $w_{i}$that add up to 1. Finally, variable importance was assessed by calculating the relative importance values derived from the model-averaged coefficients.

### 2.8 Evaluation of model performance

Although the AIC is widely used to identify the most parsimonious model among a set of candidates, it does not inherently measure how well a selected model fits observed data. Therefore, after selecting the best model based on AIC, we performed additional evaluations to assess its predictive adequacy. We also calculated the root-mean-square error (RMSE) and mean absolute error (MAE) as quantitative measures of prediction error. These diagnostics helped to assess whether the model captured the underlying data structure without systematic bias.

## 3 Results

The full model space, derived from all possible combinations of predictors in the global model, comprised 2,097,152 candidate models. Among these, Model 631,309 had the lowest AICc value (836.82), indicating that it was the most parsimonious model for the dataset. This model was used for the initial inference, although it does not account for model uncertainty because it is based on a single best-fitting model. To address the model uncertainty, we employed model averaging. The averaged model coefficients (Table 3) are weighted across a subset of models with substantial support (ΔAICc < 2), providing more robust parameter estimates by integrating model uncertainty. This subset included 30 additional models that substantially contributed to the identification of the important predictors (Table 4). For ease of reference, these models were re-indexed from M1 to M31, with M1 representing the top-ranked model. This approach provides effect sizes, standard errors, and Incidence Rate Ratios (IRRs) for each predictor, along with associated p-values, allowing for explicit interpretation of the direction and magnitude of effects.

**Table 3:** Model-averaged coefficients (Full and conditional averaging).

	Full average model ^a^				Conditional average model ^b^			
	Estimate	SE	IRR	p-value	Estimate	SE	IRR	p-value
(Intercept)	1.832	0.306	6.246	0.000	1.830	0.306	6.234	0.000
BD30m	2.394	2.847	10.957	0.402	4.590	2.340	98.494	0.052
BD60m	3.676	3.450	39.488	0.289	5.320	2.910	204.384	0.071
EpE	0.320	0.144	1.377	0.028	0.320	0.144	1.377	0.028
Loc = Kagazi	−0.042	0.217	0.959	0.849	−0.042	0.217	0.959	0.849
Loc = Munyika	−0.623	0.210	0.536	0.003	−0.623	0.210	0.536	0.003
Loc = Rusagara	0.154	0.177	1.166	0.389	0.154	0.177	1.166	0.389
MT = TC	−1.378	1.066	0.252	0.197	−1.920	0.738	0.147	0.010
MT = Suckers + TC	0.029	0.122	1.030	0.813	0.041	0.142	1.041	0.778
PSm2	1.46e-04	3.66e-05	1.000	0.001	1.35e-4	3.86e-05	1.000	0.001
SeedS	0.364	0.141	1.439	0.010	0.364	0.141	1.439	0.010
RPY	0.001	0.003	1.001	0.680	0.006	0.004	1.006	0.146
AAP	0.166	0.233	1.181	0.478	0.351	0.223	1.420	0.120
ASP	−0.068	0.170	0.934	0.690	−0.318	0.235	0.728	0.181
MFN	−0.038	0.147	0.962	0.796	−0.362	0.295	0.696	0.225
EpW	−0.009	0.054	0.991	0.866	−0.175	0.162	0.839	0.284
EpS	−0.004	0.036	0.996	0.918	−0.151	0.173	0.860	0.389

IRR = Incidence Rate Ratios.

^a^Includes all candidate models in the averaging process, regardless of whether a particular predictor is included in each model.

^b^Only includes models in which the predictor is present.

AAP – Aphids’ presence on asymptomatic mother plant, ASP – Aphids’ presence on symptomatic mother plants, BD30m - Mean of Banana density 30m (#Mats/m²) BD60m - Mean of Banana density 60m (#Mats/m²) EpE – Edge presence East; EpS – Edge presence South; EpW, Edge presence West; Loc - Location/Commune/Village; MFN – Never planting material received or collected from neighbours unknown; MT – Type of planting material; PSm2 (Plot size in m²); RPY – roguing per year farmer declaration; SeedS – Seed sources.

**Table 4:** Summary of AICc results for models relating the BBTD control and the variables.

Models		df	L	AICc	ΔAICc	$w_{i}$
**M1**	3 + 4 + 5 + 8 + 10 + 11 + 13	12	−404.97	836.82	0.00	0.06
**M2**	3 + 5 + 8 + 10 + 11 + 13	11	−406.43	837.29	0.46	0.05
**M3**	4 + 5 + 8 + 10 + 11 + 12 + 13	12	−405.25	837.39	0.57	0.04
**M4**	1 + 3 + 4 + 5 + 8 + 10 + 11 + 13	13	−404.00	837.39	0.57	0.04
**M5**	4 + 5 + 8 + 11 + 13	9	−408.96	837.53	0.71	0.04
**M6**	1 + 4 + 5 + 8 + 11 + 13	10	−407.77	837.54	0.72	0.04
**M7**	2 + 3 + 5 + 8 + 10 + 11 + 13	12	−405.34	837.58	0.75	0.04
**M8**	1 + 4 + 5 + 8 + 10 + 11 + 13	12	−405.45	837.79	0.97	0.04
**M9**	4 + 5 + 8 + 10 + 11 + 13	11	−406.69	837.80	0.98	0.04
**M10**	1 + 2 + 3 + 5 + 8 + 10 + 11 + 13	13	−404.20	837.80	0.98	0.04
**M11**	1 + 3 + 5 + 8 + 10 + 11 + 13	12	−405.49	837.88	1.05	0.04
**M12**	2 + 3 + 4 + 5 + 8 + 10 + 11 + 13	13	−404.25	837.90	1.08	0.03
**M13**	1 + 4 + 5 + 8 + 10 + 11 + 12 + 13	13	−404.32	838.03	1.21	0.03
**M14**	3 + 4 + 5 + 8 + 10 + 11 + 12 + 13	13	−404.34	838.08	1.25	0.03
**M15**	1 + 2 + 3 + 4 + 5 + 8 + 10 + 11 + 13	14	−403.11	838.18	1.35	0.03
**M16**	3 + 4 + 5 + 8 + 9 + 10 + 11 + 13	13	−404.40	838.21	1.39	0.03
**M17**	3 + 4 + 5 + 7 + 8 + 10 + 11 + 13	13	−404.41	838.22	1.40	0.03
**M18**	3 + 5 + 8 + 10 + 11 + 12 + 13	12	−405.69	838.27	1.45	0.03
**M19**	5 + 8 + 11 + 12 + 13	9	−409.33	838.28	1.46	0.03
**M20**	1 + 3 + 4 + 5 + 8 + 9 + 10 + 11 + 13	14	−403.21	838.39	1.56	0.03
**M21**	4 + 5 + 8 + 11 + 12 + 13	10	−408.22	838.44	1.62	0.03
**M22**	1 + 2 + 4 + 5 + 8 + 11 + 13	11	−407.04	838.50	1.68	0.03
**M23**	1 + 4 + 5 + 8 + 9 + 11 + 13	11	−407.05	838.52	1.70	0.03
**M24**	3 + 4 + 5 + 6 + 8 + 10 + 11 + 13	13	−404.59	838.58	1.75	0.02
**M25**	1 + 5 + 8 + 11 + 13	9	−409.50	838.62	1.80	0.02
**M26**	1 + 2 + 4 + 5 + 8 + 10 + 11 + 13	13	−404.63	838.65	1.83	0.02
**M27**	1 + 4 + 5 + 8 + 9 + 10 + 11 + 13	13	−404.64	838.68	1.86	0.02
**M28**	1 + 2 + 5 + 8 + 11 + 13	10	−408.35	838.70	1.87	0.02
**M29**	5 + 8 + 11 + 13	8	−410.73	838.75	1.93	0.02
**M30**	1 + 5 + 8 + 11 + 12 + 13	10	−408.38	838.76	1.93	0.02
**M31**	1 + 3 + 4 + 5 + 7 + 8 + 10 + 11 + 13	14	−403.40	838.77	1.94	0.02

$w_{i}$ = Akaike weight of each model; L = Loglikelihood; AICc = Akaike Information Criterion corrected.

Δ𝐀𝐈𝐂𝐜 = differences in AICc with respect to the AICc of the best candidate model, Df = degree of freedom.

1 = Aphids’ presence on asymptomatic mother plant (AAP), 2 = Aphids’ presence on symptomatic mother plants (ASP), 3 = Mean of Banana density 30m (#Mats/m²) (BD30m) 4 = Mean of Banana density 60m (#Mats/m²) (BD60m) 5 = Edge presence South (EpE); 6 = EpS, Edge presence South 7 = EpW, Edge presence West; 8 = Loc, Location/Commune/Village; 9 = MFN, Never planting material received or collected from neighbours unknown; 10 = MT, Type of planting material;11 = Plot size in m² (PSm2);12 = roguing per year farmer declaration (RPY) 13 = Seed sources (SeedS).

Model M1, the top-ranked model (AICc = 836.82, Akaike weight w_i = 0.06; Table 4), included 12 predictors and identified several significant factors associated with BBTD incidence (Table 5). Key predictors and their estimated effects were:

**Table 5:** Variable factors associated with changes in BBTD incidence.

Predictors	Coefficients	IRR	CI	p-value
(Intercept)	1.819	6.17	4.26–8.99	<0.001
BD30m	4.468	87.17	0.79–10084.68	0.050
BD60m	4.990	146.91	0.50–50055.42	0.087
EpE	0.326	1.39	1.06–1.82	0.022
Loc = Kagazi	0.000	1.00	0.66–1.54	0.999
Loc = Munyika	−0.611	0.54	0.36–0.81	0.003
Loc = Rusagara	0.220	1.25	0.89–1.74	0.190
MT = TC	−2.160	0.12	0.03–0.49	0.002
MT = Suckers + TC	0.035	1.04	0.79–1.36	0.805
PSm2	1.46e-04	1.00	1.00–1.00	<0.001
SeedS = other seeds sources	0.319	1.38	1.06–1.79	0.017

**IRR** = Incidence Rate Ratios; CI = Confidence interval.

**AAP** – Aphids’ presence on asymptomatic mother plant, **ASP** – Aphids’ presence on symptomatic mother plants, **BD30m** - Mean of Banana density 30m (#Mats/m²) **BD60m** - Mean of Banana density 60m (#Mats/m²) **EpE** – Edge presence South; **EpS** – Edge presence South; **EpW**, Edge presence West; **Loc** - Location/Commune/Village; **MFN** – Never planting material received or collected from neighbours unknown; **MT** – Type of planting material; **PSm2** (Plot size in m²); **RPY** – roguing per year farmer declaration; **SeedS** – Seed sources.

- **Plot size (PSm2; β = 1.46 × 10^-4, IRR ≈ 1.00, p < 0.001):** Although the per-square-meter coefficient is small, increasing plot size from the minimum (4 m^2^) to the maximum observed (15,624 m^2^) is associated with a substantial increase in the expected number of infected plants.
- **Mean banana density at 30 m (BD30m; β = 4.468, IRR = 87.17, 95% CI 0.79–10,084, p = 0.050):** A unit increase across the observed density range (0.002–0.378 mats/m^2^) results in a large change in predicted infection counts, reflecting the substantial influence of local banana density on disease risk.
- **Mean banana density at 60 m (BD60m; β = 4.990, IRR = 146.91, 95% CI 0.50–50,055, p = 0.087):** Similarly, variation across the observed density range (0.002–0.180 mats/m^2^) leads to meaningful differences in expected infections, emphasizing the importance of surrounding banana density at broader spatial scales.
- **Edge presence to the east (EpE; β = 0.326, IRR = 1.39, 95% CI 1.06–1.82, p = 0.022):** Presence vs. absence of a vegetation edge to the east increases expected infected plant counts by ~39%, showing a moderate and practically relevant effect at the field scale.
- **Type of planting material – tissue culture (MT = TC; β = −2.160, IRR = 0.12, 95% CI 0.03–0.49, p = 0.002)**: Using tissue culture plantlets compared with suckers reduces expected infection counts by ~88%, demonstrating a strong protective effect despite categorical coding.
- **Seed source (SeedS = other sources; β = 0.319, IRR = 1.38, 95% CI 1.06–1.79, p = 0.017):** Using alternative seed sources increases expected infection counts by ~38%, reflecting a meaningful influence of seed system practices.
- **Location Munyika (β = −0.611, IRR = 0.54, 95% CI 0.36–0.81, p = 0.003):** Compared to the reference location, expected infection counts in Munyika are ~ 46% lower, highlighting strong local differences in disease incidence.

Other predictors, such as aphid presence (AAP, ASP), roguing frequency (RPY), edge presence south/west (EpS, EpW), and never receiving planting material from neighbours (MFN), were not statistically significant in the averaged model, although some exhibited non-negligible IRRs (Table 5).

**Fig 3:**
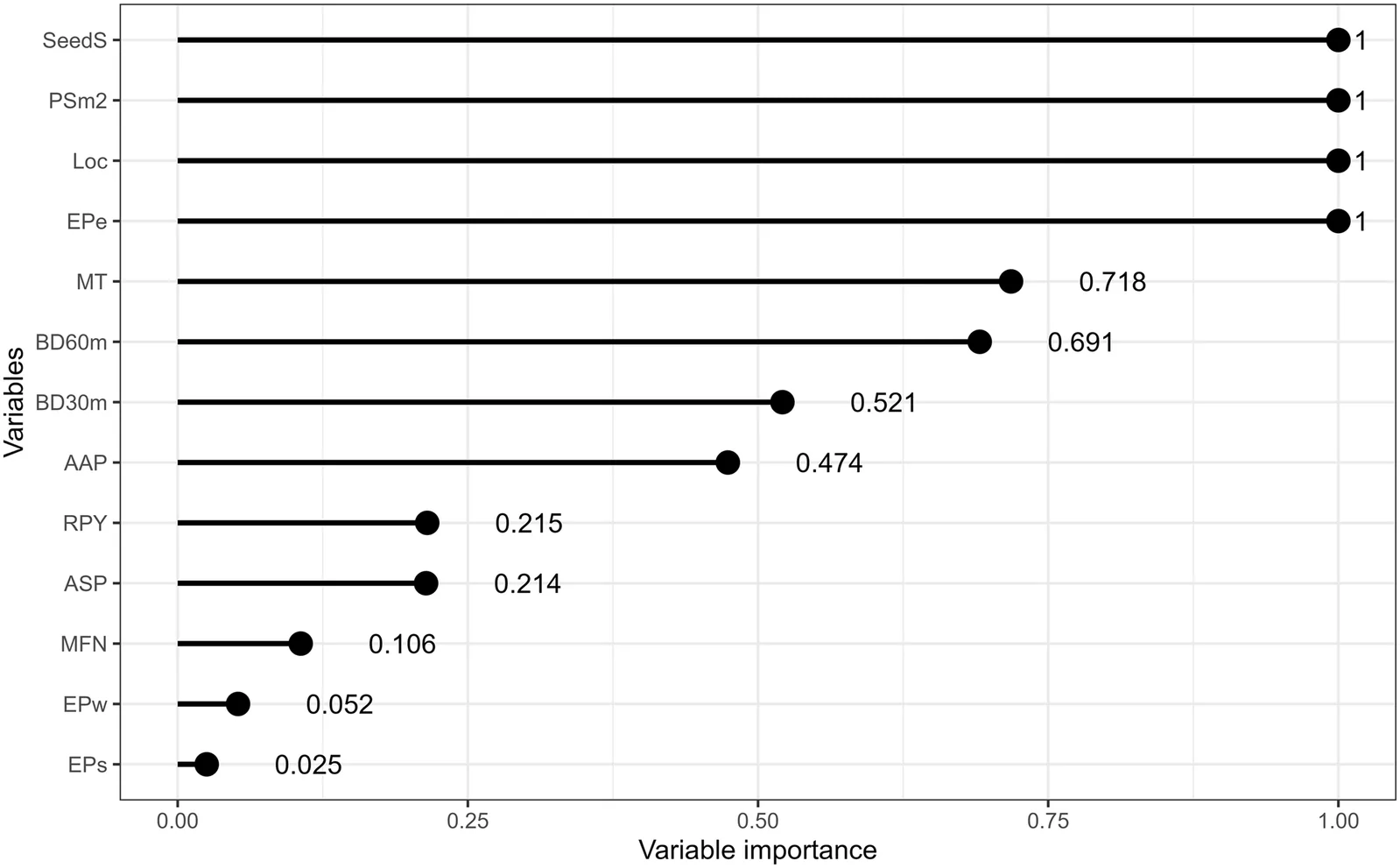
Relative importance of selected variables based on the frequency of inclusion in the best combination of models.

Model M2, the second-best model, included all predictors in M1, except for banana density within a 30–60-meter radius, resulting in a model with one fewer parameter. Models M2–M31 varied in the combination of included variables, often omitting some significant predictors or including others, but all had higher AICc values than M1. These differences primarily stem from the number of parameters estimated and reflected in the varying degrees of freedom. Approximately half of the models in the subset had between 9 and 11 degrees of freedom, with additional parameters contributing little explanatory power while increasing model complexity [37]. This added complexity reduces the model quality by increasing the number of coefficients to be estimated without improving the fit. Fig 3 illustrates the relative importance of each variable across the retained models. Higher values indicate a greater likelihood of a given variable appearing in the best supported models. Overall, the best-performing models for BBTD management aligned with the four main crop management strategies are listed in descending order of representation: (1) context-specific factors, such as garden location and plot size (28%), (2) seed system characteristics (26%), (3) banana density around the farm (16%) and vegetation barriers (10%), and (4) vector density (10%) and disease inoculum management (3%).

**Fig 4:**
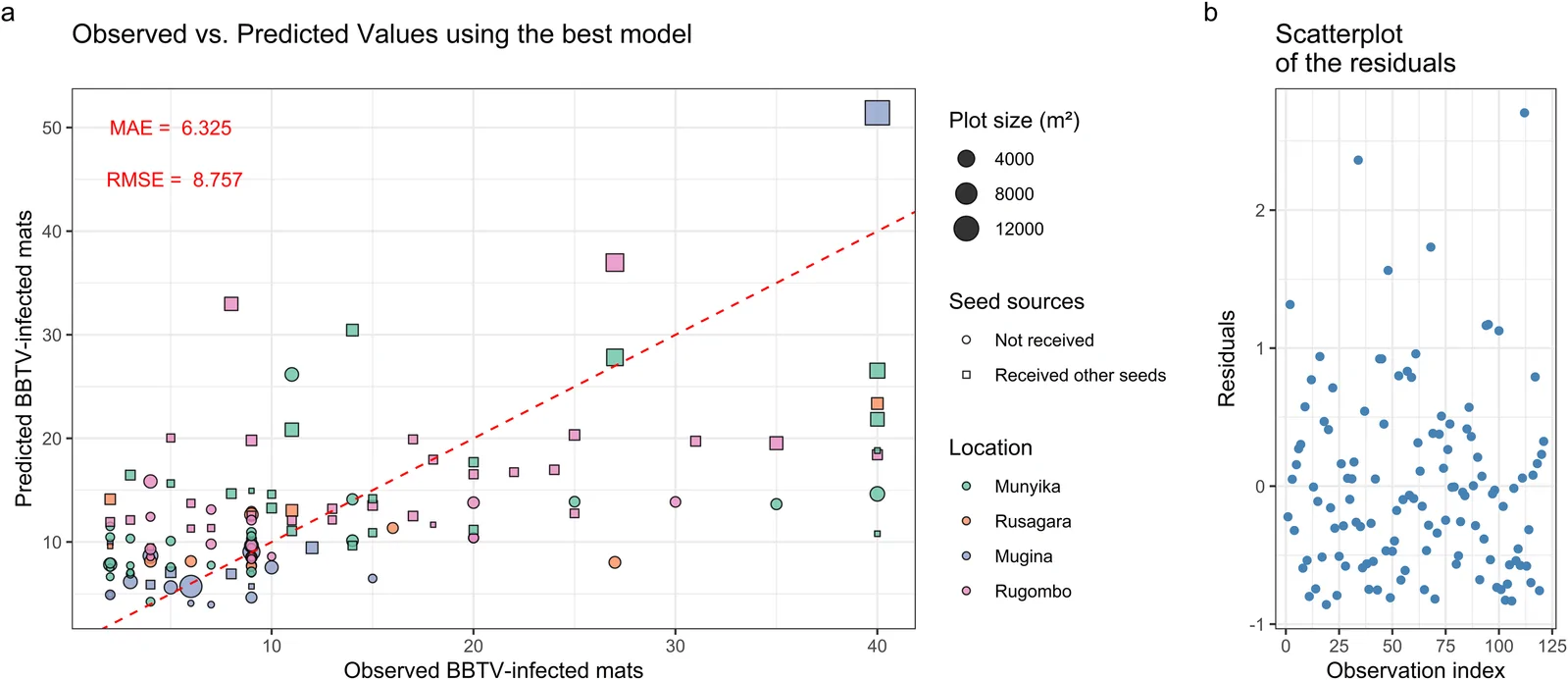
Observed vs. Predicted values and residual diagnostics for best model fit. The symbol size corresponds to field size, filled colours represent location and symbol shape indicate seed system. These variables were chosen based on Fig 4, as they are the top three most important across all models.

The best model residual diagnostics (Fig 4) indicate some heteroscedasticity, but no extreme departures from model assumptions. Mean Absolute Error (MAE = 6.325) and Root Mean Square Error (RMSE = 8.757) suggest that, on average, predicted counts were within ~6–9 plants of observed counts. The slightly higher RMSE than MAE indicates occasional larger deviations, consistent with wide confidence intervals for some predictors (e.g., BD30m, BD60m). Despite some small effect sizes or high uncertainty in certain variables, the model provides interpretable estimates of the main factors affecting BBTD incidence at the landscape scale. These estimates can guide management strategies while acknowledging uncertainty in precise effect magnitudes.

A stable core group of variables (Location; Plot size, Seed sources and Edge Presence East) appeared in nearly all models, indicating that these predictors consistently formed the backbone of the model set. A second group of predictors—including aphid density on asymptomatic plants, aphid density on symptomatic plants, banana density, and the presence of field edges to the south and west—occurred in most candidate models, typically extending the core model structure. Intermediate predictors, such as aphid presence on asymptomatic plants and banana density within 30 m, appeared in approximately half of the models, whereas tissue culture seed use and roguing were included only occasionally in exploratory model variants. This pattern indicates varying levels of model support across predictors, with some consistently retained and others appearing only in a subset of plausible models. Finally, rare predictors (tissue culture seed and roguing) were included only sporadically in exploratory model variants. Together, these patterns indicate that most candidate models represent variations around a common core predictor structure, with additional predictors providing incremental explanatory refinement. Together, these patterns suggest that most candidate models represent variations around a common core predictor structure, with additional predictors providing incremental explanatory refinement. Across the 31 candidate models, predictor inclusion showed a clear hierarchical pattern.

## 4 Discussion

Farmers initiating the recovery of BBTD from a common point using the same training protocol, in Burundi, resulted in varying levels of disease prevalence in their fields. Although farmers had received training and technical guidance, implementation of the protocol—including cultivar choice, field expansion, and garden maintenance—depended largely on individual farmer decisions and resource availability. This starting point– allowed us to assess the effect of ecologically grounded management strategies and disease environments while minimising the effect of disease arrival at each farm, hence offers insights into adaptable approaches for disease mitigation in similar agroecological settings. Our analysis therefore attempts to identify additional candidate measures for BBTD management previously not included the hazard reduction protocol [18]. Future experiments could focus on evaluating the utility of these measures in controlled background infection and landscape matrices, to improve our understanding on their practical utility and predictive power of the models for informing BBTD management strategies.

Using negative binomial regression to account for over-dispersed infection counts and AICc-based model selection with model averaging, we identified the factors most consistently associated with reinfection under endemic conditions. Across the best-supported models (ΔAICc < 2), landscape context (location and plot size), seed system characteristics, banana density within 30–60 m buffers, and presence of vegetation barriers were the most influential categories. The best-supported individual model accounted for only a modest proportion of total Akaike weight, indicating model uncertainty. Consequently, inference was based on patterns consistent across model subsets rather than reliance on a single model. Within the limits of this observational study, structural landscape features and seed sourcing integrity were more strongly associated with reinfection risk than short-term measures of vector abundance or symptomatic disease prevalence. In contrast, direct vector density variables and roguing frequency had lower inclusion likelihood and weaker statistical support in model averaging. Overall, BBTD prevalence was associated with factors linked to direct introduction of BBTV and to the movement of plant vectors. Landscape level complexity provides a relevant opportunity for the management of BBTD at community scale.

Landscape context and structural characteristics of gardens were consistently associated with infection levels. Location, particularly Munyika commune, was significantly associated with lower disease counts, suggesting that reinfection risk varies geographically, likely reflecting agro-ecological, topographical, or socio-management differences. Plot size also appeared frequently in supported models, indicating that structural properties of fields influence reinfection dynamics. While larger plots are more likely to relate to greater investment in banana production hence better surveillance, it could also be linked to gender (access to land and farming resources). These findings suggest a greater background disease drive or existing infection rate, given that BBTD is already endemic in this region. Also, Munyika is the lowest lying and could be more preferable for aphid movement. Indeed, in Rwanda, Burundi and DRC, BBTD spread has been restricted to lower lying Bugarama/Rusizi valley [38].

Farm, buffer and landscape level diversity and landscape-scale crop management practices could play an important role in BBTD control. In this study, banana canopy density around the recovered plot (connectivity between target plot and neighbouring plots) were associated with an increased BBTD risk in the target plot and retained in final selected variable combinations. These factors could influence of vector movement between mats and gardens [39]. Also, the presence of vegetation barriers, was an important factor in the final variables set associated with lower BBTD levels. While the significance of hedges in only one direction is intriguing, structural discontinuities in canopy connectivity may reduce vector immigration or movement. Aphid migration is wind assisted, as aphids are not strong fliers, and so their migration are likely to follow predominant wind direction [40]. This finding highlights the potential of non-host vegetation hedges and windbreaks to enclose gardens and minimise spread of viruliferous aphids between gardens, another candidate low-cost option for BBTD management. Banana aphids have a narrow host-spectrum and are directly affected by host distribution and BBTV status of the hosts [41–43]. Behavioural manipulation of insect pests and vectors by mixing non-host species barriers is a powerful tool in cultural pest management [44]. Planning cropping matrices at the landscape level would also fit well with community approaches to BBTD management, especially when the choice of the most effective hedge species was identified.

Surprisingly, the observed BBTD prevalence in surrounding gardens did not consistently appear among the strongest predictors of infection in the target plot. Also it has been reported that BBTD makes plants more attractive and supportive of aphid population density [41,45]. It would be expected that gardens with greater BBTD density would be more important sources of BBTD. However, this result would underline the importance of aphid movement and not population alone in BBTD spread, an observation already known in persistent transmission mechanisms. Thus, where there is high underlying disease pressure, measures to minimize aphid movement galleries (e.g., windbreaks, hedges and non-host crop associations) could be tested as potential BBTD management tools. A notable limitation of this finding is that, our assessment of BBTD prevalence was strictly based on counts of plants showing disease symptoms and could exclude BBTV-infected mats in the latent stage. The proportion of these plants relative to diseased plants could not be established and their role as BBTV sources to aphids is not well understood. In an earlier study in the same landscapes, asymptomatic plants represented 1–2% of diseased plants observed [19]. Also, Chabi et al., [46] showed that the onset of BBTV transmission is closely associated with the onset of the first symptoms [46]. We hypothesis that a clear understanding of the relationship of disease prevalence and inoculum pressure could be clarified with studies involving high precision diagnostic tools to accommodate the most important correlates.

The role of infected asymptomatic banana mats remains unclear in our study, as only diseased plants were included. The distinction between infection, infectivity, and symptom expression, which is well documented in cavendish systems [23], was not made in this study. Incubation time, and hence the onset of symptom expression, varies across cultivars and may also be influenced by the physiological conditions of the infected plant [46,47]. These factors could affect the detection and management of BBTD. Asymptomatic banana mats could influence the model if such plants are significantly infectious [48]. A delay between the appearance of the first symptom and infectivity was found in Cavendish and could also vary among banana cultivars [47]. Although our study did not distinguish between infected, diseased, and infectious plants in the fields studied, Chabi et al. [46] had found infectiousness of plants to be closely related to symptom appearance. As disease management is not uniform among the farm households studied, realistic estimates are difficult to obtain. Similarly, disease progress has been shown to vary among cultivars [49], seasons [50] and can vary according to the altitude and age of crops [51].

Seed system variables showed consistent inclusion in most of the models of parameter association with BBTD reinfection. Under the BBTD recovery project, farmers had been expected to multiply and recycle low-risk planting material derived from the initial batch of virus-free tissue culture plants supplied through the programme [19,20]. Accordingly, seed sourcing was analysed by assessing declared seed sources, seed types used and as a binary variable distinguishing farmers who relied exclusively on project-derived material from those who supplemented their planting stock with material obtained from other sources. Seed sourcing practices are widely recognized as primary determinants of BBTD risk and remain the dominant pathway for introducing the virus into new landscapes [14,52]. In the BBTD recovery project, five tissue-cultured cultivars had been distributed, with each farmer receiving up to three; however, gardens that incorporated additional planting material from outside formal project channels were significantly more likely to report higher infection levels. While this outcome is epidemiologically expected [52], the strength of seed-related variables suggests both a high underlying BBTD prevalence in the broader landscape and the limited reliability of symptom-based selection alone as a means of ensuring seed health. These findings quantitatively reinforce the importance of sustained clean seed governance beyond the initial replanting phase. At the same time, informal seed systems remain essential to farmers because they are often the only source of locally preferred cultivars unavailable through formal channels [11]. Seed sourcing decisions typically reflect varietal preference, perceived disease risk, and the absence of accessible mechanisms to verify planting material health [10]. Managing seed health, alongside measures to limit reinfection within fields, is therefore central to effective BBTD control [52]. However, strengthening clean seed systems in endemic regions presents logistical and social challenges, including limited availability of preferred landraces and gender-related barriers to resource access [53]. In mixed-cultivar smallholder systems—where varietal diversity may provide buffering against other diseases such as Fusarium—maintaining strict control over seed sourcing is particularly complex, especially given that acquisition of new cultivars is often opportunistic and mediated through informal networks of trust and experience [10,54].

The removal of diseased bananas around the target plot before planting and after planting has been an important previously. Yet, farmer-reported roguing frequency and aphid presence variables were less consistently retained in the best-supported models and were not statistically robust in model averaging. Roguing consisted of uprooting and destroying all plants in any mat with a single symptomatic shoot [20]. The pseudostem was quickly buried in a shallow trench to miniise aphid migration, while the rhizome was chopped into small pieces and dried above ground to stop any sprouting. Asymptomatic banana mats next to the diseased one were not removed, following the protocol of Omondi et al., [19]. Roguing can significantly reduce BBTD rates, lowering incidence to 2–10% when applied consistently and early in the disease expression stage [19]. Rather, the cross-sectional design captures cumulative infection at a single time point and does not allow separation of infection pressure from removal dynamics. The relationship between roguing and infection is complex: higher reinfection rates may lead to increased roguing, whereas effective roguing may reduce infection. Time-series studies are needed to disentangle these dynamics and better quantify roguing efficacy. Besides, reported scouting and roguing intensity may differ significantly from actual roguing, its timing after disease detection and its correct application to avoid canopy disturbance, which could increase aphid migration risk. Detecting the temporal and operational effects of roguing intensity would require repeated measurements over time.

Ecological interactions between the virus, vector and the banana host were relevant to this study. To date, BBTV has not been shown to infect other Zingiberales species grown in the region, but the virus has been reported in other species, while the non-banana vector *Pentalonia caladii* has also been observed in bananas (Wessels et al., 2016; [55] and is known to be a competent vector of BBTV [42]. Both *P. caladii* and *P. nigronervosa* can complete their life cycles on several hosts other than banana, although *P. caladii* has a broader host range, while *P. nigronervosa* is largely restricted to *Musa* spp. in nature (Foottit, 2010; Wessels et al., 2015). Despite both being competent BBTV vectors, *P. nigronervosa* is the more efficient transmitter. In bananas, host susceptibility and symptom expression likely contribute to disease dynamics, with evidence suggesting cultivar-level variations in BBTV accumulation and symptom severity [46,47,49,50]. Aphid colonization and reproduction may also vary by cultivar, influencing both vector population and transmission efficiency. Additionally, BBTD can alter the host preference of non-viruliferous *P. nigronervosa*, an effect lost once the aphid acquires the virus [45,56]. Also, bananas with BBTD support faster vector population growth compared to healthy plants [41]. Thus, virus manipulation on the host and the vector could enhance transmission in endemic landscapes [57]. Determining the effect of BBTD on aphid migration would be clarify the epidemiological consequences of the pathogen manipulation observation.

Our findings provide insight into the practical aspects of BBTD management. First, seed systems determine the introduction of BBTV into new gardens and landscapes and should be managed especially using indexed planting material on the one hand; and a strategy on the inclusion of farmer desired landraces in clean seed multiplication systems. Our observation on the protection of seed health agrees with modelling projections of Mapinda et al. [52]. Landscape complexity seems to function through limiting both vector abundance and dispersal. For instance, generalist natural enemies may play an important role in keeping aphid populations low and preventing population build-up, which triggers dispersal. They also accelerate nymphal mortality, reducing the time spent on the host plant to acquire and inoculate the virus [58]. Studies on the specific dimensions of these correlates are important to determine their potential to improve a farmer-friendly package for BBTD management.

The findings from the model assessment against field data highlight the potential error risk when interpreting outputs from a single “best model”, even a model selected using AIC. Since no single best model fully captures the underlying data structure, including persistent heteroscedasticity and non-random residual patterns (Fig 4), making model averaging as a more reliable inferential strategy. The averaged model (Table 3), combining estimates from multiple models weighted by their AIC support, provides a broader and more stable inference framework that explicitly incorporates uncertainty across the model space. This is especially valuable in ecological and epidemiological studies where model uncertainty is substantial [59–62]. The heteroscedasticity and residual patterns indicate that BBTD spread is influenced by factors that our linear models do not fully capture, likely including non-linear thresholds, interactions among predictors, or spatial heterogeneity in transmission mechanisms, showing that ecological system exhibits s context-dependent dynamics. This interpretation aligns with biological knowledge of BBTD, where seed and vector transmission dynamics may vary under different agroecological conditions. Consequently, while the model-averaged coefficients provide the best available estimates of average effects across the study area, they should be interpreted as approximations of general tendencies rather than precise, universal constants. Thus, the observed statistical uncertainty reflected in our models reflects underlying ecological heterogeneity.

Several limitations should be considered when interpreting these findings. The study was based on a cross-sectional assessment conducted three years after replanting, limiting inference on temporal infection dynamics of disease and vector dynamics. Observed disease prevalence reflect cumulative reinfection and management responses and do not allow separation of infection rate from removal processes. Although negative binomial modelling and AICc-based model averaging accounted for overdispersion and model uncertainty, some predictors exhibited wide confidence intervals, indicating variability in effect size estimates. Vector presence was assessed through field observation rather than direct measurement of dispersal or viruliferous status. Also, the species of vectors was not determined by molecular markers. Wessels et al., [43] has shown that a small proportion of aphids collected on banana could be *P. caladii*, a competent BBTV vector species, whose role in BBTV transmission in the field could be influenced by subsequent host preferences (Foottit et al., 2010). Further, asymptomatic infections were not quantified beyond symptom-based inspection. While this could underestimate BBTV estimation due to exclusion of asymptomatic plants, the symptom evaluation protocol used [50] has been found reliable and consistent in subsequent studies (e.g., [19,46]. Additionally, unmeasured factors such as microclimatic variation, cultivar-specific susceptibility/ BBTD symptom expression, and social network–mediated seed exchange may influence reinfection patterns. Longitudinal studies incorporating dynamic infection monitoring and direct vector movement data would strengthen causal inference and refine landscape-based management recommendations.

## 5 Conclusions

Over the past 50 years, quarantine exclusion, clean seed systems and roguing, have been the most common BBTD management options [17–19,23,63–65]. These approaches have been useful, especially in regions of low cultivar diversity, stringent quarantine systems and adequate surveillance capacity; and where periodic renewal of plantations is practiced. However, smallholder banana production systems in Africa are dominated by weak quarantine systems, high cultivar diversity and perennial production systems. Overall, our results highlight the interaction of landscape characteristics and field practices in shaping the BBTD dynamics in these systems. Three key factors in BBTD re-introduction into clean fields were revealed: landscape complexity, farmers’ seed movement, and host density, which can be consistently manipulated to achieve sustainable control. The most important variables appearing in the 31 best models reveal the importance of clean seed systems, control of vector movement, and a general reduction of inoculum pressure around the recovered plot in the sustainable management of BBTD. The success of these approaches may vary significantly according to latitude (or location) and weather conditions. Thus, common farmer cropping practices, such as intercropping and strip cropping, can be assessed as means to retard BBTD spread. Also, non-host hedges could supplement the buffer zones in the protection of recovered banana gardens from BBTV especially in high density landscapes with small farms. Similarly, clean seed sourcing and roguing diseased plants are essential low-cost practices that are available to farmers. We recommend that research extends these findings by using dynamic longitudinal data to evaluate the performance of IPM packages under different contexts. Additionally, an evaluation of gender-linked performance of identified technologies and practices, could support developing inclusive and sustainable integrated BBTD management strategies.

## Supporting information

S1 Data Copy of BBTD dataset. (XLSX)

## References

[1] Hulme P. Trade, transport and trouble: managing invasive species pathways in an era of globalization. J Appl Ecol. 2009;46:10–8. doi: 10.1111/j.1365-2664.2008.01600.x

[2] Meyerson LA, Mooney HA. Invasive alien species in an era of globalization. Front Ecol and Environ. 2007;5:199–208. doi: 10.1890/1540-9295(2007)5[199:IASIAE]2.0.CO;2

[3] FAOSTAT. FAO statistical database. 2023. Accessed 2023 June 1. https://www.fao.org/faostat/en/#data/QC

[4] Adheka JG, Dhed’a DB, Karamura D, Blomme G, Swennen R, De Langhe E. The morphological diversity of plantain in the Democratic Republic of Congo. Scientia Horticulturae. 2018;234:126–33. doi: 10.1016/j.scienta.2018.02.034

[5] Fainou M, Ewédjè EE, Kifouli A, Gustave L, Djedatin G, Affokpon A, et al. Diversity of local varieties of banana and plantain cultivated in Benin. IJBC. 2018;10:497–509. doi: 10.5897/IJBC2018.1232

[6] Karamura DA. Numerical taxonomic studies of the East African highland bananas (Musa spp. AAA-East Africa) in Uganda. Montpellier, France: University of Reading. 1999. https://hdl.handle.net/10568/104967

[7] Mbo Nkoulou LF, Tchinda Ninla LA, Cros D, Martin G, Ndiang Z, Houegban J, et al. Analysis of genetic diversity and agronomic variation in banana sub-populations for genomic selection under drought stress in southern Benin. Gene. 2023;859:147210. doi: 10.1016/j.gene.2023.147210 36681099

[8] Swennen R, Vuylsteke D, Ortiz R. Phenotypic diversity and patterns of variation in West and Central African plantains (Musa spp., AAB group Musaceae). Econ Bot. 1995;49:320–7. doi: 10.1007/BF02862352

[9] Jacobsen K, Omondi BA, Almekinders C, Alvarez E, Blomme G, Dita M, et al. Seed degeneration of banana planting materials: strategies for improved farmer access to healthy seed. Plant Pathology. 2018;68(2):207–28. doi: 10.1111/ppa.12958

[10] Nduwimana I, Sylla S, Xing Y, Simbare A, Niyongere C, Garett K, et al. Banana seed exchange networks in Burundi - linking formal and informal systems. Outlook Agric. 2022;:15. doi: 10.1177/00307270221103288

[11] Simbare A, Sane CAB, Nduwimana I, Niyongere C, Omondi BA. Diminishing farm diversity of East African highland bananas in banana bunchy top disease outbreak areas of Burundi—the effect of both disease and control approaches. Sustainability. 2020;12:7467. doi: 10.3390/su12187467

[12] Munhoz T, Vargas J, Teixeira L, Staver C, Dita M. Fusarium Tropical Race 4 in Latin America and the Caribbean: status and global research advances towards disease management. Front Plant Sci. 2024;15:1397617. doi: 10.3389/fpls.2024.1397617 39081528 PMC11286425

[13] Pemsl D, Staver C, Creamer B, Abdoulaye T, Alene A, Rusike J. Results of a global online expert survey: Major constraints, opportunities and trends for banana production and marketing and priorities for future RTB banana research RTB. 2014. https://hdl.handle.net/10568/83418

[14] Stainton D, Martin DP, Muhire BM, Lolohea S, Halafihi M, Lepoint P, et al. The global distribution of Banana bunchy top virus reveals little evidence for frequent recent, human-mediated long distance dispersal events. Virus Evol. 2015;1(1):vev009. doi: 10.1093/ve/vev009 27774281 PMC5014477

[15] Kumar PL, Hanna R, Alabi OJ, Soko MM, Oben TT, Vangu GHP, et al. Banana bunchy top virus in sub-Saharan Africa: investigations on virus distribution and diversity. Virus Res. 2011;159(2):171–82. doi: 10.1016/j.virusres.2011.04.021 21549775

[16] Kumar PL, Selvarajan R, Iskra-Caruana ML, Chabannes M, Hanna R. Biology, etiology, and control of virus diseases of banana and plantain. Adv Virus Res. 2015;91:229–69. doi: 10.1016/bs.aivir.2014.10.006 25591881

[17] Lepoint PCE, Staver C, Ajambo S, Rietveld AM, Kumar LP, Hanna R, et al. Workshop report: strategic planning workshop on recovering Banana production in BBTD affected areas: community and farm household approaches. 2014. https://cgspace.cgiar.org/server/api/core/bitstreams/c158a76e-0f5d-4256-abc6-6fd1a0dc39d8/content

[18] Lepoint P. Recovering banana production in BBTD affected areas: Strengthening cross-site learning tools in epidemiology, gender and social relations, and participatory experimentation approaches. CGIAR Research Program on Roots, Tubers and Bananas (RTB). 2016. doi: 10.13140/RG.2.1.1039.6323

[19] Omondi BA, Soko MM, Nduwimana I, Delano RT, Niyongere C, Simbare A, et al. The effectiveness of consistent roguing in managing banana bunchy top disease in smallholder production in Africa. Plant Pathology. 2020;69(9):1754–66. doi: 10.1111/ppa.13253

[20] Omondi AB, Skilton RA, Kumar PL, Munyua LM, Bahama JB, Niassy S. Guide to Banana Bunchy Top Disease: Surveillance, Control and Production Recovery. First ed. AU-IAPSC. 2026.

[21] Niyongere C, Losenge T, Ateka EM, Ntukamazina N, Ndayiragije P, Simbare A, et al. Understanding banana bunchy top disease epidemiology in Burundi for an enhanced and integrated management approach. Plant Pathology. 2012;62(3):562–70. doi: 10.1111/j.1365-3059.2012.02676.x

[22] Omondi BA, Soko MM, Chabi M, Nduwimana I, Adjalla CC, Athindehou F, et al. Tools for the management of the banana bunchy top disease in small holder systems. Acta Hortic. 2023;(1367):233–42. doi: 10.17660/actahortic.2023.1367.27

[23] Allen RN. Epidemiological factors influencing the success of roguing for the control of bunchy top disease of bananas in New South Wales. Aust J Agric Res. 1978;29:535–44. doi: 10.1071/AR9780535

[24] Dato KMG, Dégbègni MR, Atchadé MN, Zandjanakou Tachin M, Hounkonnou MN, Aman Omondi B. Spatial parameters associated with the risk of banana bunchy top disease in smallholder systems. PLoS One. 2021;16(12):e0260976. doi: 10.1371/journal.pone.0260976 34860836 PMC8641891

[25] Djailo BD, Lokana J, Ngama F, Nkosi BI, Blomme G. Systemicity of banana bunchy top viral infection in the Kisangani region of the Democratic Republic of Congo. Afr J Agric Res. 2016;11:527–32. doi: 10.5897/ajar2015.10641

[26] Robson J, Wright M, Almeida R. Within-Plant Distribution and Binomial Sampling of Pentalonia nigronervosa (Hemiptera: Aphididae) on Banana. J Econ Entomol. 2006;99:2185–90. doi: 10.1603/0022-0493-99.6.2185 17195692

[27] Magee CJP. Investigation on the bunchy top disease of the banana. Melbourne: H.J. Green, Government Printer. 1927. https://nla.gov.au/nla.obj-52819684/view

[28] Azur MJ, Stuart EA, Frangakis C, Leaf PJ. Multiple imputation by chained equations: what is it and how does it work?. MPR. 2011;20(1):40–9. doi: 10.1002/mpr.329 PMC3074241 21499542

[29] Anselin L. Local Indicators of Spatial Association—LISA. Geographical Analysis. 1995;27(2):93–115. doi: 10.1111/j.1538-4632.1995.tb00338.x

[30] Cliff AD, Ord JK. Spatial autocorrelation. London: Pion. 1973.

[31] Bivand RS, Wong DWS. Comparing implementations of global and local indicators of spatial association. TEST. 2018;27(3):716–48. doi: 10.1007/s11749-018-0599-x

[32] R Core Team. R: A Language and Environment for Statistical Computing. Vienna, Austria: R Foundation for Statistical Computing. 2025.

[33] Siril H, Gitagno D, Kaaya S, Caputo M, Hirschhorn L, Nyamuhanga T, et al. Generalized and COVID related anxiety as risk factors for health outcomes among adolescents with HIV during COVID-19 in Tanzania. Res Sq. 2024;:rs.3.rs-3921926. doi: 10.21203/rs.3.rs-3921926/v1 38410463 PMC10896391

[34] Cavanaugh JE, Neath AA. The Akaike information criterion: Background, derivation, properties, application, interpretation, and refinements. WIREs Computational Stats. 2019;11(3). doi: 10.1002/wics.1460

[35] Burnham KP, Anderson DR. Model selection and inference: A practical information-theoretic approach. New York: Springer-Verlag. 1998. doi: 10.1007/978-1-4757-2917-7

[36] Portet S. A primer on model selection using the Akaike Information Criterion. Infect Dis Model. 2020;5:111–28. doi: 10.1016/j.idm.2019.12.010 31956740 PMC6962709

[37] Snipes M, Taylor DC. Model selection and Akaike Information Criteria: An example from wine ratings and prices. Wine Economics and Policy. 2014;3(1):3–9. doi: 10.1016/j.wep.2014.03.001

[38] Sebasigari K, Stover RH. Banana Diseases and Pests in East Africa: Report of a Survey Made in November 1987. Montpellier, France: INIBAP. 1988.

[39] Kebede Y, Baudron FF, Bianchi F, Tittonell P. Unpacking the push-pull system: Assessing the contribution of companion crops along a gradient of landscape complexity. Agric Ecosyst Environ. 2018;268:115–23. doi: 10.1016/j.agee.2018.09.012

[40] Parry HR, Evans AJ, Morgan D. Aphid population response to agricultural landscape change: A spatially explicit, individual-based model. Ecol Model. 2006;199:451–63. doi: 10.1016/j.ecolmodel.2006.01.006

[41] Adjalla CC, Madodé YE, Omondi BA. The banana bunchy top disease enhances vector survival: effect of the banana bunchy top disease on host suitability of the banana aphid Pentalonia nigronervosa. Int J Trop Insect Sci. 2025;45(4):1579–95. doi: 10.1007/s42690-025-01539-y

[42] Watanabe S, Greenwell AM, Bressan A. Localization, concentration, and transmission efficiency of Banana bunchy top virus in four asexual lineages of Pentalonia aphids. Viruses. 2013;5(2):758–76. doi: 10.3390/v5020758 23435241 PMC3640525

[43] Robbertse N, Omondi BA, Millar IM, Krüger K, Jooste AEC. Non-destructive DNA extraction from aphids: the application in virus - vector studies of Banana bunchy top virus (BBTV). Eur J Plant Pathol. 2019;153(2):571–82. doi: 10.1007/s10658-018-1552-2

[44] Zhang Z, Sun X, Luo Z, Gao Y, Chen Z. The manipulation mechanism of “push–pull” habitat management strategy and advances in its application. Acta Ecologica Sinica. 2013;33(2):94–101. doi: 10.1016/j.chnaes.2013.01.005

[45] Murhububa IS, Bragard C, Tougeron K, Hance T. Preference of Pentalonia nigronervosa for infected banana plants tends to reverse after Banana bunchy top virus acquisition. Sci Rep. 2024;14(1):2993. doi: 10.1038/s41598-024-53205-x 38316887 PMC10844331

[46] Chabi M, Dassou AG, Adoukonou-Sagbadja H, Thomas J, Omondi AB. Variation in Symptom Development and Infectivity of Banana Bunchy Top Disease among Four Cultivars of Musa sp. Crops. 2023;3(2):158–69. doi: 10.3390/crops3020016

[47] Ngatat S, Hanna R, Lienou J, Ghogomu RT, Nguidang SPK, Enoh AC, et al. Musa Germplasm A and B Genomic Composition Differentially Affects Their Susceptibility to Banana Bunchy Top Virus and Its Aphid Vector, Pentalonia nigronervosa. Plants. 2022;11(9):1206. doi: 10.3390/plants11091206 35567207 PMC9100355

[48] Cunniffe NJ, Stutt ROJH, van den Bosch F, Gilligan CA. Time-dependent infectivity and flexible latent and infectious periods in compartmental models of plant disease. Phytopathology. 2012;102(4):365–80. doi: 10.1094/PHYTO-12-10-0338 22106830

[49] Ngatat S, Hanna R, Kumar PL, Gray SM, Cilia M, Ghogomu RT, et al. Relative susceptibility of Musa genotypes to banana bunchy top disease in Cameroon and implication for disease management. Crop Protection. 2017;101:116–22. doi: 10.1016/j.cropro.2017.07.018

[50] Niyongere C, Ateka E, Losenge T, Blomme G, Lepoint P. Screening musa genotypes for banana bunchy top disease resistance in Burundi. Acta Hortic. 2011;(897):439–47. doi: 10.17660/actahortic.2011.897.60

[51] Leclerc M, Doré T, Gilligan CA, Lucas P, Filipe JAN. Estimating the Delay between Host Infection and Disease (Incubation Period) and Assessing Its Significance to the Epidemiology of Plant Diseases. PLoS ONE. 2014;9(1):e86568. doi: 10.1371/journal.pone.0086568 PMC3899291 24466153

[52] Mapinda JJ, Hugo AK, Shinzeh JK, Edward S. Modelling the effects of virus-resistant planting material on the transmission dynamics of banana bunchy top disease. Sci Rep. 2025;15(1):20010. doi: 10.1038/s41598-025-94881-7 40481122 PMC12144305

[53] McEwan MA, Almekinders CJ, Andrade-Piedra JJ, Delaquis E, Garrett KA, Kumar L, et al. “Breaking through the 40% adoption ceiling: Mind the seed system gaps.” A perspective on seed systems research for development in One CGIAR. Outlook Agric. 2021;50(1):5–12. doi: 10.1177/0030727021989346 33867584 PMC8022077

[54] Nkengla-Asi L, Omondi AB, Che Simo V, Assam E, Ngatat S, Boonabaana B. Gender dynamics in banana seed systems and impact on banana bunchy top disease recovery in Cameroon. Outlook Agric. 2020;49(3):235–44. doi: 10.1177/0030727020918333

[55] Mendoza NAP, Mendoza JS, Thomas JE, Dela Cueva FM. Alternative hosts of banana bunchy top virus in the Philippines and the first evidence of seed transmission of BBTV. Front Plant Sci. 2024;15:1467331. doi: 10.3389/fpls.2024.1467331 39665103 PMC11633669

[56] Safari Murhububa I, Tougeron K, Bragard C, Fauconnier M-L, Bisimwa Basengere E, Walangululu Masamba J, et al. Banana Tree Infected with Banana Bunchy Top Virus Attracts Pentalonia nigronervosa Aphids Through Increased Volatile Organic Compounds Emission. J Chem Ecol. 2021;47(8–9):755–67. doi: 10.1007/s10886-021-01298-3 34463893

[57] Ingwell LL, Eigenbrode SD, Bosque-Pérez NA. Plant viruses alter insect behavior to enhance their spread. Sci Rep. 2012;2:578. doi: 10.1038/srep00578 22896811 PMC3419366

[58] Roudine S, Le Lann C, Bouvaine S, Le Ralec A, van Baaren J. Can biological control be a strategy to control vector-borne plant viruses?. J Pest Sci. 2023;96:451–70. doi: 10.1007/s10340-022-01587-0

[59] Dormann CF, Calabrese JM, Guillera‐Arroita G, Matechou E, Bahn V, Bartoń K, et al. Model averaging in ecology: A review of Bayesian, information‐theoretic, and tactical approaches for predictive inference. Ecol Monogr. 2018;88:485–504. doi: 10.1002/ecm.1309

[60] Grueber CE, Nakagawa S, Laws RJ, Jamieson IG. Multimodel inference in ecology and evolution: challenges and solutions. J Evol Biol. 2011;24(4):699–711. doi: 10.1111/j.1420-9101.2010.02210.x 21272107

[61] Gutierrez EE, Heming NM. Introducing AIC model averaging in ecological niche modeling: a single-algorithm multi-model strategy to account for uncertainty in suitability predictions. arXiv preprint. 2018. doi: 10.48550/arXiv.1807.04346

[62] Young C. Model uncertainty and the crisis in science. Socius. 2018;4. doi: 2378023117737206

[63] Abiola A, Zandjanakou-Tachin M, Aoudji KNA, Avocevou-Ayisso C, Kumar PL. Adoption of Roguing to Contain Banana Bunchy Top Disease in South-East Bénin: Role of Farmers’ Knowledge and Perception. International Journal of Fruit Science. 2019;20(4):720–36. doi: 10.1080/15538362.2019.1673277

[64] Lepoint P, Sibomana R, Niyongere C, Blomme G. Cultural practices for banana bunchy top disease management: a sustainable option for Burundian smallholders. In: Van den Bergh I, editor. Towards Sustainable Global Production and Improved Uses Proc. Int. ISHS-ProMusa Symposium. on Bananas and Plantains. 2013. 111–8.

[65] Retkute R, Zandjanakou-Tachin M, Omondi BA, Agoi UR, Vodounou YM, Akpla A, et al. Controlling Banana Bunchy Top Disease in Benin: Crop Protection Strategies with Socioeconomic Perspectives. People, Plants and Planet. 2025. doi: 10.1101/2025.07.24.666611

