# Supplementary material for: Drivers of banana re-infection in newly planted gardens in banana bunchy top disease endemic zones: Implications for the recovery of banana production
Source: PLoS One. 2026 Sep 11;21(9):e0357834. doi: 10.1371/journal.pone.0357834 (PMC13567820; doi:10.1371/journal.pone.0357834)
Supplement: Supplementary file 1 [file pone.0357834.s003.pdf]

PLOS ONE

Drivers of Banana Re-infection in Newly Planted Gardens in Banana Bunch top Disease Endemic Zones: Implications for the recovery of banana production

--Manuscript Draft--

**Manuscript Number:**	PONE-D-25-42829R1
**Article Type:**	Research Article
**Full Title:**	Drivers of Banana Re-infection in Newly Planted Gardens in Banana Bunch top Disease Endemic Zones: Implications for the recovery of banana production
**Short Title:**	Epidemiology of Banana bunchy top disease reinfection in recovered gardens
**Corresponding Author:**	Aman Bonaventure Omondi<br>Bioversity International: Alliance of Bioversity International and International Center for Tropical Agriculture<br>Cotonou, BENIN
**Keywords:**	Banana Bunchy Top Disease; integrated pest and disease management; small holder farming; cropping mixtures; spatial epidemiology
**Abstract:**	1. Abstract<br><br>Understanding how crop pests and diseases spread within and between fields can inform the design of effective integrated pest and disease management strategies. This study aimed to identify the key landscape, field design, and management factors influencing the persistence and re-infection of Banana Bunchy Top Disease (BBTD) in endemic smallholder systems. We assessed 121 banana gardens reestablished with virus-free tissue-culture plantlets, three years prior, to determine the combination of practices associated with low BBTD incidence. Positive associations with disease prevalence included high banana density within a 60 m radius, elevated aphid populations, greater varietal diversity, and the use of locally sourced planting material. In contrast, higher altitude, the presence of hedgerows, and frequent rogueing were negatively associated with BBTD. Model selection indicated that aphid prevalence, banana density, rogueing frequency, and seed source appeared in more than half of the best-fit models, underscoring their importance in minimizing reinfection and yield loss. These findings emphasize the value of coordinated, community-based replanting with virus-free material, regular rogueing informed by farmer symptom recognition, and the integration of barrier hedges or mixed cropping to restrict aphid dispersal. Further research should investigate optimal species combinations and landscape configurations to enhance BBTD suppression in smallholder production systems.
**Order of Authors:**	Aman Bonaventure Omondi
	Delano Ronald Togbé
	Renata Retkute
	Innocent Nduwimana
	Niyongere Celestin
	Staver Charles
**Response to Reviewers:**	Reviewer #1: Thank you for your clear and well-written work. I am interested in your manuscript and want to learn more about your research. Please see my questions below.<br>RESPONSE: We appreciate your kind remarks. Please find our detailed responses below.<br>Abstract:<br>The abstract should clearly state the research objectives to guide the reader and frame the study<br>Response: We agree to this observations<br>ACTION: We have added the objective linked to the approach (Lines 3-7): "This study aimed to identify the key landscape, field design, and management factors influencing the persistence and re-infection of Banana Bunchy Top Disease (BBTD) in endemic

smallholder systems. We assessed 121 banana gardens reestablished with virus-free tissue-culture plantlets, three years prior, to determine the combination of practices associated with low BBTD incidence."

Methods:
1. What environmental and management factors contribute to the reinfection of Banana Bunchy Top Disease (BBTD)?
ACTION: We have added details and reference on lines 123-127: "The spread of BBTV through the vector is favoured by environmental factors that support vector aphid population build up and migration, and rapid plant growth and accumulation of BBTV titre to enable vector acquisition. These include favourable temperature, wind and topography (Niyongere et al., 2013). Thus, the greatest BBTD risk has been associated with humid lowlands."

2. Could you please elaborate on the rationale behind selecting farms exclusively replanted with BBTV-free tissue culture plantlets, considering the potential influence of pre-existing disease incidence in the study area on the observed BBTD reinfection rates?
RESPONSE: This is an important question. The BBTD existence in the vicinity provides comparable source risk around the target plots, which is important for eliminating spatial characteristics that would be associated with a new introduced area where BBTD would still be in specific hotspots. The selection of only replanted fields provides a uniform starting point and eliminates local disease history around specific topographies as a confounding factor. This way, plot history becomes more important and factors associated with active management practices can be identified.
Naturally in a BBTD infested landscape, different farms would be at different levels of epidemy development linked to the landscape and management attributes but also to the age of the plantations. By choosing the replanted gardens, we had a unique opportunity to have multiple farms that started at the same time, with comparable management systems, not much cultivar variation and whose differences would bring us closer to understanding landscape and management-linked factors influencing BBTD prevalence.
ACTION: We revised text on lines 132-139: " As all farms shared a known replanting start date with virus-free material, they provided a consistent baseline for assessing the environmental and management factors influencing BBTD reinfection. The presence of BBTD in the surrounding landscape ensured a comparable source of inoculum across sites, minimizing spatial bias that would otherwise occur in newly affected areas where infections remain localized in hotspots. By focusing on replanted fields, we eliminated the confounding effects of local disease history and topographic variation, allowing plot-level history and active management practices to emerge as the main explanatory factors."

3. Based on the provided methodology, what specific measures were implemented to mitigate potential biases arising from subjective visual assessments of BBTD incidence during data collection?
RESPONSE: The data collection team was first trained on the implementation of the protocol in a one-week workshop outside of the data collection area. A uniform protocol with disease classification thresholds was used. BBTD symptoms follow progression from incipient levels (very easy to misdiagnose) to clearer ones at a later stage. Data taking involved cross-auditing a fraction of the gardens between data takers, evaluating the same mats previously classified.
We therefore believe the resulting bias would be random and end up in a general underestimation or overestimation. The research team had been trained in BBTD identification and had been used in the same community as trainers for two years. Our own research in Malawi, Benin, DRC and more recently in Rwanda showed that with minimal field training, farmers become quite accurate in detecting BBTD. A study done in Rwanda to assess performance and retention of BBTD identification knowledge a year after training showed practically the same result.
ACTION: Added after line 148-151: "The data collection team underwent standardized training using a uniform disease classification protocol, implemented cross-auditing of observations between data collectors, and leveraged experienced personnel previously trained and validated in accurate BBTD identification across multiple countries."

Discussion:

1. What specific strategies were employed to ensure that the imputation process did not introduce bias or distort the true relationships within the dataset, particularly given the observed overdispersion and the eventual use of the NBRM?
Renata/Dato/
RESPONSE: Missing categorical variables were imputed using multiple imputation by chained equations (MICE). Multiple imputation generates several predictions for each missing value, allowing subsequent analyses to account for uncertainty in the imputations and produce accurate standard errors. When the observed data provide limited information about the missing values, imputations may vary widely, leading to larger standard errors, whereas highly predictive observed data yield more consistent imputations and smaller, yet still accurate, standard errors (Azur et al., 2011).
ACTION: We have added after line 178: To assess potential bias from imputation, we examined the standard errors of the imputed values.

2. How might the observed heteroscedasticity in the residuals and the small effect sizes of some statistically significant predictors in the best model (M1) be addressed to improve the practical utility and predictive power of future BBTD management strategies?

RESPONSE: We thank the reviewer for this insightful question.
ACTION: We added after line 369-374: "However, our analysis provides valuable insights into the shortcomings of some aspects of data availability. For example, the widest confidence interval ranges for the IRR were observed for the mean banana density at 30 m and 60 m. Designing an experimental protocol that captures greater variability (i.e., distance between rows and columns) could improve the practical utility and predictive power of the models for informing BBTD management strategies."

3. What effect does promptly removing diseased banana plants (roguing) have on BBTV infection rates, and what evidence suggests a need for time-series studies to tease apart the intricate link between roguing frequency, reinfection, and overall disease dynamics?
RESPONSE: Prompt roguing slows down within field infection rates and slows down disease progress. Allen 1987, had indicated the link between disease appearance and infection acquisition by aphids, resulting in disease spread. In a separate study, we showed that consistent roguing could limit disease spread and promote recovery of fields previously under BBTD infestation in field experiments in Malawi, Benin and Burundi (Omondi 2020). Roguing also supported low risk seed availability for recovery within the fields. Roguing however may not prevent primary infection arriving into the field by seed or through immigrating aphids, hence the need for consistency and landscape scale application.
ACTION: We revised text on lines 445-450: "Roguing can significantly reduce BBTV infection rates, lowering incidence to 2-10% when applied consistently and early in the disease expression stage (Omondi et al., 2020). However, the emergence of new infections in both managed and non-managed farms followed a seasonal cycle (Omondi et al., 2020), highlighting the need for time-series studies to clarify how roguing frequency, disease pressure, and environmental factors interact to shape long-term BBTD dynamics.."

4. What are the ecological interactions among BBTV, its aphid vectors, the banana plants, and other possible host plants (including non-banana Zingiberales and vectors like P. caladii), and how do factors like cultivar susceptibility, aphid colonization, virus-driven changes in vector behavior, climate variation, plot size, seed/plant movement, and gender or resource access affect BBTV spread and epidemics?
RESPONSE: Studies have been carried out to asses the host diversity of banana aphids Pentalonia nigronervosa versus P. caladii. These include:
That both can complete their life cycles in various hosts other than banana and that P. caladii has a wider host range including banana; while P. nigronervosa tends to be limited to Musa spp (Foottit 2010; Wessels et al 2015). Both species are competent vectors of BBTV banana, but P. nigronervosa is more efficient. BBTV infection changes the host preference of non-viruliferous P. nigronervosa; but this preference is lost once the aphid acquires the virus (Muhurbaba et a., 2023; 2025). So there if virus manipulation that could accelerate the rate of spread in a landscape where the disease is present. Besides, we have shown recently that aphids growing on diseased plants multiply almost twice as fast as in healthy plants. Together these mean any presence

of BBTV in the plots would strongly increase disease spread due to greater vector population output. However, for our study these factors are included in multiple dimensions including presence of aphids, density of bananas and presence of BBTD infested plants. While these are difficult to test in such a multiple scale, we note that these factors could affect observed model performance, such as significance of banana and of aphid numbers but not necessarily of BBTD infected plants.
ACTION: We added to Lines 389- 391: “The availability of viable BBTV susceptible hosts and the presence of backyard gardens could influence vector dispersal and virus spread dynamics. “
ACTION:
1. We change text on lines 458 from “Ecological interactions between the virus, vector, banana host, and other possible alternative host plants were relevant to this study.” to “Ecological interactions between the virus, vector, banana host, and other possible alternative host plants are highly relevant to understanding BBTD dynamics, but they are difficult to test under natural infection conditions due to their complexity.”
2. We also added after line 463: “Both P. caladii and P. nigronervosa can complete their life cycles on several hosts other than banana, although P. caladii has a broader host range, while P. nigronervosa is largely restricted to Musa spp. (Foottit, 2010; Wessels et al., 2015). Despite both being competent BBTV vectors, P. nigronervosa is the more efficient transmitter.

5. What are the primary drivers of Pentalonia nigronervosa population surges in Burundi-like landscapes? How far do viruliferous aphids disperse across typical smallholder mosaic landscapes, and which habitat features facilitate or hinder movement.

RESPONSE: To date, the actual range of aphid movement has not been measured directly. We have already added the following text the methods which partially covers the question: “The spread of BBTV through the vector is favoured by environmental factors favouring vector aphid population build up and migration, and rapid plant growth and accumulation of BBTV titre to enable vector acquisition. These include favourable temperature, wind and topography (Niyongere et al., 2013). Thus, the greatest BBTD risk BBTD has been associated with humid lowlands.” – Line 123

6. What are the practical implications of model averaging for interpreting BBTV risk in different agroecological zones, and how should field experiments be designed to identify context-specific drivers (e.g., aphid-driven vs. wind- or ant-mediated spread) to inform adaptive BBTD management strategies?
RESPONSE: Controlled experiments on cropping mixtures, non-host barriers and ant control have been suggested, to assess their effect on aphid distribution and disease spread. The possible role of non-host intercrops on natural enemies could also be similarly investigated; and preliminary results in our hands show that such complexity affects natural enemies in different ways. Ant studies would be interesting, especially in assessing the effects of repellents and biorational agents in BBTD control (such as soap and botanical pesticides). To date our experiments, show little effect on aphid presence, but the effects on ants would suggest a confounding relationship on aphids. The field level management options presented here, could influence ants and aphids in diverse ways. Our focus is on their association with resultant disease appearance at this stage. Once the list of important associations is whittled down, systematic field tests could be done to validate their efficacy and come up with the best combination of cost-effective measures for BBTD management. This effort could field experimentation, parameter estimation from field experiments followed by scenario modelling (cf Mapinda et al., 2025).
ACTION: We rephrased the last sentence of the Discussion Line 503: “Field experiments targeting such context-specific factors by manipulating levels of cropping mixtures content, presence of non-host barriers or selective levels of aphid and ant control should be conducted to offer more precise insights and support adaptive management strategies for BBTD.”

7. Is there selection pressure on BBTV or vectors leading to more virulent strains or evasion of control measures?
RESPONSE: This is an interesting question and could require very specific studies. Indeed we know that the non-viruliferous aphids prefer infected plants; which also support faster growth of aphid populations (Adjalla et al., 2025; Muhurbuba et al, 2023;

Powered by Editorial Manager® and ProduXion Manager® from Aries Systems Corporation

	2024). We also know that viruliferous aphids do not discriminate between infected and non-infected plants. Thus, in the presence of infected plants in a garden, there will be a net migration to spread the virus through unequal attraction and then equal dispersals. A possible mechanism of selection for virulence could involve the delay of symptom appearance relative to the period of infectiousness of diseased plants. Allen (1987) established that three leaves would show symptoms before aphids begin migrating out and becoming an infection threat to other plants. This represents a period ranging from 3 weeks in young plantlets in the rainy season (rapid growth) to about six weeks in older plants Ngatat et al., (2017; 2022) and Chabi et al (2023) showed a variation in symptom appearance. Chabi also showed that transmission was always associated with symptom appearance such that banana aphids could not acquire BBTV from asymptomatic plants previously exposed toBBTV. As systemic virus, the duration of inoculation acquisition time and population multiplication on infected plants are important in BBTD transmission (Roudine et al 2023). So, evolution of invasion response could be strains showing symptoms much later when the virus titre can already be acquired and transmitted by aphids. This has not been studied yet but would be interesting.<br>ACTION: We have added after line 470-473:" BBTV infection can alter the host preference of non-viruliferous P. nigronervosa, an effect lost once the aphid acquires the virus (Muhurbaba et al., 2023; 2024), suggesting virus manipulation that could enhance transmission in endemic landscapes."
**Additional Information:**	
**Question**	**Response**
**Financial Disclosure**<br><br>Enter a financial disclosure statement that describes the sources of funding for the work included in this submission. Review the submission guidelines for detailed requirements. View published research articles from *PLOS ONE* for specific examples.<br><br>This statement is required for submission and **will appear in the published article** if the submission is accepted. Please make sure it is accurate.<br><br>**Funded studies**<br>Enter a statement with the following details:<br>• Initials of the authors who received each award<br>• Grant numbers awarded to each author<br>• The full name of each funder<br>• URL of each funder website<br>• Did the sponsors or funders play any role in the study design, data collection and analysis, decision to publish, or preparation of the manuscript?<br><br>Did you receive funding for this work?	Yes

Please add funding details.<br>as follow-up to "**Financial Disclosure**<br><br>Enter a financial disclosure statement that describes the sources of funding for the work included in this submission. Review the submission guidelines for detailed requirements. View published research articles from *PLOS ONE* for specific examples.<br><br>This statement is required for submission and **will appear in the published article** if the submission is accepted. Please make sure it is accurate.<br><br>**Funded studies**<br>Enter a statement with the following details:<br>• Initials of the authors who received each award<br>• Grant numbers awarded to each author<br>• The full name of each funder<br>• URL of each funder website<br>• Did the sponsors or funders play any role in the study design, data collection and analysis, decision to publish, or preparation of the manuscript?<br><br>Did you receive funding for this work?"	Funding for this work was supported by the CIGAR Research Program on Root Tubers and Bananas (RTB) for ABO, AS, EN, CS, and CN at the Alliance of Bioversity International and CIAT and ISABU Burundi. RK, ABO, and CS were also supported by a Gates Foundation Grant on BBTD in Nigeria and Benin [INV010652], while RR was supported by The Gates Foundation Grant [INV070408]. Under these grant conditions of the Foundation, a Creative Commons Attribution 4.0 Generic License has already been assigned to the Author Accepted Manuscript version that might arise from this submission. Further funding at the time of ABO was supported by the Plant Health Initiative of the One-CGIAR, which is grateful for the support of CGIAR Trust Fund contributors (www.cgiar.org/funders).<br>The funders had no role in study design, data collection and analysis, decision to publish, or preparation of the manuscript.
Please select the country of your main research funder (please select carefully as in some cases this is used in fee calculation).<br>as follow-up to "**Financial Disclosure**<br><br>Enter a financial disclosure statement that describes the sources of funding for the work included in this submission. Review the submission guidelines for detailed requirements. View published research articles from *PLOS ONE* for specific examples.<br><br>This statement is required for submission and **will appear in the published article** if the submission is accepted. Please make sure it is accurate.	BENIN - BJ

**Funded studies**<br>Enter a statement with the following details:<br>• Initials of the authors who received each award<br>• Grant numbers awarded to each author<br>• The full name of each funder<br>• URL of each funder website<br>• Did the sponsors or funders play any role in the study design, data collection and analysis, decision to publish, or preparation of the manuscript?<br><br>Did you receive funding for this work?"	
**Competing Interests**<br><br>Use the instructions below to enter a competing interest statement for this submission. On behalf of all authors, disclose any competing interests that could be perceived to bias this work—acknowledging all financial support and any other relevant financial or non-financial competing interests.<br><br>This statement is required for submission and **will appear in the published article** if the submission is accepted. Please make sure it is accurate and that any funding sources listed in your Funding Information later in the submission form are also declared in your Financial Disclosure statement.<br><br>View published research articles from *PLOS ONE* for specific examples.	The authors have declared that no competing interests exist.

**NO authors have competing interests**<br><br>Enter: *The authors have declared that no competing interests exist.*<br><br>**Authors with competing interests**<br><br>Enter competing interest details beginning with this statement:<br><br>*I have read the journal's policy and the authors of this manuscript have the following competing interests: [insert competing interests here]*<br><br>* typeset	
**Ethics Statement**<br><br>Enter an ethics statement for this submission. This statement is required if the study involved:<br><br>• Human participants<br>• Human specimens or tissue<br>• Vertebrate animals or cephalopods<br>• Vertebrate embryos or tissues<br>• Field research<br><br>Write "N/A" if the submission does not require an ethics statement.<br><br>General guidance is provided below. Consult the submission guidelines for detailed instructions. **Make sure that all information entered here is included in the Methods section of the manuscript.**	N/A

**Format for specific study types**<br><br>**Human Subject Research (involving human participants and/or tissue)**<br>• Give the name of the institutional review board or ethics committee that approved the study<br>• Include the approval number and/or a statement indicating approval of this research<br>• Indicate the form of consent obtained (written/oral) or the reason that consent was not obtained (e.g. the data were analyzed anonymously)<br><br>**Animal Research (involving vertebrate animals, embryos or tissues)**<br>• Provide the name of the Institutional Animal Care and Use Committee (IACUC) or other relevant ethics board that reviewed the study protocol, and indicate whether they approved this research or granted a formal waiver of ethical approval<br>• Include an approval number if one was obtained<br>• If the study involved *non-human primates*, add *additional details* about animal welfare and steps taken to ameliorate suffering<br>• If anesthesia, euthanasia, or any kind of animal sacrifice is part of the study, include briefly which substances and/or methods were applied<br><br>**Field Research**<br><br>Include the following details if this study involves the collection of plant, animal, or other materials from a natural setting:<br>• Field permit number<br>• Name of the institution or relevant body that granted permission	
**Data Availability**<br><br>Authors are required to make all data underlying the findings described fully available, without restriction, and from the time of publication. PLOS allows rare exceptions to address legal and ethical concerns. See the PLOS Data Policy and FAQ for detailed information.	Yes - all data are fully available without restriction

A Data Availability Statement describing where the data can be found is required at submission. Your answers to this question constitute the Data Availability Statement and **will be published in the article**, if accepted.<br><br>**Important:** Stating 'data available on request from the author' is not sufficient. If your data are only available upon request, select 'No' for the first question and explain your exceptional situation in the text box.<br><br>Do the authors confirm that all data underlying the findings described in their manuscript are fully available without restriction?	
**Describe where the data may be found in full sentences. If you are copying our sample text, replace any instances of XXX with the appropriate details.**<br><br>• If the data are **held or will be held in a public repository**, include URLs, accession numbers or DOIs. If this information will only be available after acceptance, indicate this by ticking the box below. For example: *All XXX files are available from the XXX database (accession number(s) XXX, XXX.).*<br>• If the data are all contained **within the manuscript and/or Supporting Information files**, enter the following: *All relevant data are within the manuscript and its Supporting Information files.*<br>• If neither of these applies but you are able to provide **details of access elsewhere**, with or without limitations, please do so. For example:<br><br>*Data cannot be shared publicly because of [XXX]. Data are available from the XXX Institutional Data Access / Ethics Committee (contact via XXX) for researchers who meet the criteria for access to confidential data.*<br><br>*The data underlying the results presented in the study are available from (include the name of the third party*	Data has been submitted as Supplementary material with the manuscript

and contact information or URL).<br>• This text is appropriate if the data are owned by a third party and authors do not have permission to share the data.<br><br>* typeset	
Additional data availability information:	

Powered by Editorial Manager® and ProduXion Manager® from Aries Systems Corporation

Clean revised manuscript Click here to access/download;Manuscript;Omondi et al BBTD PLOS ONE_21stOct.docx

Drivers of Banana Re-infection in Newly Planted Gardens in Banana Bunch top Disease Endemic Zones: Implications for the recovery of banana production

Aman Bonaventure Omondi[1], Delano Ronald Togbé[2], Renata Retkute[3], Innocent Nduwimana[4], Niyongere Celestin[5,6], Staver Charles[7,8].

1. *Bioversity International, Alliance of Bioversity International and CIAT, c/o IPGRI Building, IITA Campus, Abomey-Calavi, 08BP 0932, Cotonou, Benin.*
2. *Faculty of Agronomic Sciences, University of Abomey-Calavi, Benin*
3. *Epidemiology and Modeling Group, Department of Plant Sciences, University of Cambridge, Downing Street, Cambridge CB2 3EA, UK*
4. *Bioversity International, The Alliance of Bioversity International and CIAT, co/ IITA Building, Regina Mundi Road, Bujumbura, Burundi.*
5. *Institute of Agronomic Sciences, (ISABU) Rohero 1, Avenue de la Cathédrale, B.P 795 Bujumbura, Burundi,*
6. *CAB International (CABI), Quartier Kabondo Ouest,10, Avenue du Large, PO Box 1995, Bujumbura, Burundi*
7. *Bioversity International, Parc Scientifique Agropolis 1990 bd Lironde, 34980 Montferrier sur Lez, Montpellier, France.*
8. *Faculty of Agricultural Sciences, Universidad Veracruzana, Xalapa, Mexico*

Authors' ORCID numbers
Aman Bonaventure Omondi - 0000-0001-9711-5645
Delano Ronald Togbé - 0000-0002-0080-8021
Renata Retkute: 0000-0002-3877-6440
Innocent Nduwimana - 0000-0001-7110-354X
Niyongere Celestin (n/a)
Charles Staver (CS): 0000-0002-4532-6077

*Author for correspondence: B.A. Omondi (b.a.omondi@cgiar.org).

†Present address: Bioversity International, Alliance of Bioversity International and CIAY, IITA Campus, c/o IPGRI Building, IITA Campus, Abomey Calavi, 08 BP 0932, Cotonou, Benin

1. **Abstract**

Understanding how crop pests and diseases spread within and between fields can inform the design of effective integrated pest and disease management strategies. This study aimed to identify the key landscape, field design, and management factors influencing the persistence and re-infection of Banana Bunchy Top Disease (BBTD) in endemic smallholder systems. We assessed 121 banana gardens reestablished with virus-free tissue-culture plantlets, three years prior, to determine the combination of practices associated with low BBTD incidence. Positive associations with disease prevalence included high banana density within a 60 m radius, elevated aphid populations, greater varietal diversity, and the use of locally sourced planting material. In contrast, higher altitude, the presence of hedgerows, and frequent rogueing were negatively associated with BBTD. Model selection indicated that aphid prevalence, banana density, rogueing frequency, and seed source appeared in more than half of the best-fit models, underscoring their importance in minimizing reinfection and yield loss. These findings emphasize the value of coordinated, community-based replanting with virus-free material, regular rogueing informed by farmer symptom recognition, and the integration of barrier hedges or mixed cropping to restrict aphid dispersal. Further research should investigate optimal species combinations and landscape configurations to enhance BBTD suppression in smallholder production systems.

Keywords: Banana Bunchy Top Disease, integrated pest and disease management, small holder farming, cropping mixtures, spatial epidemiology

2. Introduction

Invasive species represent a major threat to agricultural productivity and the global stability of ecosystems. Their proliferation, often driven by global trade, climate change, and human-mediated landscape alterations, disrupts local ecological balances and exacerbates crop loss (Meyerson & Mooney, 2007; Hulme, 2009). In agroecosystems, invasive pests and pathogens can spread rapidly, especially in landscapes with conducive habitats and susceptible hosts in the absence of natural enemies and without coordinated management. Many pests and diseases rapidly establish in new areas of incursion depending on host availability, dispersal mechanisms (including effective vectors), and spatial connectivity between suitable habitats across the landscape. Understanding how host distribution and landscape connectivity influence pest and disease dynamics is important for managing invasive plant pathogens and insect vectors which have become endemic. At the local scale, the spread of disease is influenced by field-level management practices, such as sanitation, crop diversity, and planting patterns, which affect the inoculum pressure and vector behaviour. Spatial modelling of these agro-environmental factors can help to identify ecological thresholds and intervention points for disease control.

In Sub-Saharan Africa, more than 50 types and cultivars of banana (including plantain, *Musa* spp.) are cultivated (Swennen et al., 1995; Karamura, 1999; Adheka et al., 2018, Fainou et al., 2018; Mbo et al., 2023), a vital staple and cash crop contributing to food security and income for millions of smallholder farmers (FAOSTAT 2023). As a perennial crop, banana supports year-round harvesting, which is especially important for rural households' food and economic security. The banana fruit and various plant parts are consumed in multiple forms depending on the variety. Dessert bananas are eaten fresh as fruits, whereas cooking bananas and plantains serve as starchy staples or snacks. Some varieties are used to produce processed beverages, such as banana beer and juice, while others are considered multipurpose because of their suitability for several uses. This wide range of uses makes banana an

essential crop for food security, nutrition, and income generation, especially in smallholder farming systems across tropical regions. However, yields across Africa remain among the lowest globally, primarily due to a range of biotic stressors, including pests and diseases often transmitted in locally sourced seed and low land productivity with few external inputs (Jacobsen et al., 2019). The case of banana landscapes in Burundi illustrates the challenges of invasive diseases. Farm communities in the Rift Valley lost fields of the previously dominant beer banana variety, 'Kayinja', to Fusarium wilt race 1. They replaced 'Kayinja' with 'Yangambi Km5' only to face the proliferation of Banana Bunchy Top Disease (BBTD) (Simbare et al., 2020) which is the subject of this paper. Another Fusarium strain, Tropical Race 4 (TR4), has reached sub-Saharan Africa but is not yet widespread. Many cultivars across the region are susceptible to TR4 (Munhoz et al 2024). Sustainable management of these invasive species is therefore essential for securing food systems and livelihoods on the continent, especially among smallholder farmers.

Banana bunchy top disease, caused by the banana bunchy top virus (BBTV), is one of the most destructive viral diseases affecting bananas (Stainton et al., 2015). It is transmitted by the banana aphid (*Pentalonia nigronervosa*) and infected planting material. Once established, the disease causes a progressive yield loss. More seriously, the local supply of clean banana suckers diminishes as, progressively, more banana mats become infected by BBTD. Farmers lose access to BBTV-free planting material as local seed supply degenerates (Jacobsen et al 2019) and banana production collapses. Currently, BBTD has spread to at least 18 countries in Africa and is estimated to affect 6–12 million smallholder households (Kumar et al., 2011; 2015). In heavily affected regions such as Malawi and the Democratic Republic of the Congo (DRC), production losses have significantly undermined livelihoods (Kumar et al., 2011). Moreover, BBTD poses a serious threat to banana genetic diversity in two centres of secondary diversity of cultivated bananas: The East African Highlands (home to over 100 AAA-EA types) (Karamura, 1999; Adheka et al., 2018) and West Africa, the centre of plantain diversity (Fainou et al., 2018; Swennen et al., 1995). Such genetic loss may threaten the long-term sources of genetic material for banana improvement against other emergent biotic and

abiotic challenges. Small-holder production systems, dominated by local landraces, are particularly vulnerable because of their dependence on informal seed sourcing and the perennial low-input nature of the production system (Simbare et al., 2020).

In 2014, The Learning Alliance for BBTD control in Africa (https://www.bbtvalliance.org/), an alliance of international agricultural research centres, sub-Saharan national research organizations, and rural communities undertook banana production recovery efforts in BBTD-affected landscapes in Burundi and eight other countries using a community-based collaborative learning and action approach (Lepoint et al 2014, Lepoint et al 2016). This strategy involved landscape-scale destruction of potentially infected plants, enforcement of a banana-free fallow period to eliminate inoculum sources and viruliferous aphids, multiplication of disease-free tissue culture planting material, replanting in well-isolated fields, and collaborative monitoring of replanted gardens (Omondi et al., 2020; 2023). Effective implementation requires strong landscape-level coordination, particularly in crop monitoring, inoculum reduction by consistent roguing, and the adoption of clean seed supply systems (Omondi et al, 2021).

Understanding the risk of reinfection in recovered banana gardens affected by BBTD is an important component of long-term disease management and the recovery of banana production in BBTD-affected landscapes. Maintaining a low and stable reinfection risk allows for the gradual expansion of recovered areas, utilizing existing buffer zones and facilitating the growth of seed production and exchange systems within these zones. The recovery strategy deployed in Burundi offered a case study to monitor reinfection in 121 small farms and two community nurseries for tissue culture plants in four villages. Each farmer's field offered a suite of local factors, although the area itself was characterized by small mixed crop perennial banana gardens and backyard banana mats. At a distance, these landscapes resemble large plantations in terms of banana canopy continuity, but they are a complex mosaic of

diverse banana varieties, associated crops, and landscape features such as hedgerows, fallow fields, rice monocrops, and farming practices across an altitudinal gradient.

Our study aimed to identify the key risk factors associated with BBTD reinfection in replanted banana fields to improve the current banana recovery strategies. By assessing the prevalence of BBTD in farms that were replanted with BBTV-free tissue culture planting materials in 2013 and 2014, we aimed to evaluate the factors associated with disease transmission. A single assessment of the reinfection status of initially clean plots across farms with similar background risk provided a unique opportunity to evaluate the risk pressures of BBTD prevalence while minimizing the influence of temporal variability.

3. Materials and Methods

a. Study Area and Farm Selection

A field study was conducted on 121 farms across four communes— Kagazi, Rusagara, Munyika, and Gitebe—in Cibitoke Province, Burundi. Cibitoke Province is a major banana-growing region and a known hotspot for Banana Bunchy Top Disease (BBTD). It lies along the border between Burundi, Rwanda, and the Democratic Republic of Congo (DRC), spanning altitudes from 800 to 1300 m above sea level. Rusizi Valley, shared among these three countries, has been endemic to BBTD for over three decades. The spread of BBTV through the vector is favoured by environmental factors favouring vector aphid population build up and migration, and rapid plant growth and accumulation of BBTV titre to enable vector acquisition. These include favourable temperature, wind and topography (Niyongere et al., 2013). Thus, the greatest BBTD risk BBTD has been associated with humid lowlands. Pilot sites were established in selected communes in 2014 to evaluate community-based approaches to BBTD recovery (Omondi et al., 2020, 2023). The farms assessed in this study had been replanted using BBTV-free tissue culture (TC) plantlets sourced from a private tissue culture propagation laboratory (Agrobiotec Limited, Bujumbura, Burundi) and hardened in community-based nurseries. While

farmers were trained and encouraged to implement BBTD risk reduction and cultural management practices, implementation varied based on individual capacities and priorities. As all farms shared a known replanting start date with virus-free material, they provided a consistent baseline for assessing the environmental and management factors influencing BBTD reinfection. The presence of BBTD in the surrounding landscape ensured a comparable source of inoculum across sites, minimizing spatial bias that would otherwise occur in newly affected areas where infections remain localized in hotspots. By focusing on replanted fields, we eliminated the confounding effects of local disease history and topographic variation, allowing plot-level history and active management practices to emerge as the main explanatory factors

b. Data Collection

The data were collected using a combination of direct field measurements and structured surveys. A sketch map of each target garden was created to orient the characterization and data acquisition of the field itself and a 90m surrounding observational zone corresponding to the buffer proposed to reduce aphid movement to the field. Thus, each of the 121 gardens planted with tissue culture (TC) banana plantlets in the BBTD recovery effort three months prior, was treated as a focal plot. The buffer was divided into here zones, following Allen (1978), observation that aphid arrival to a field declined with distance from the field up to 90 m, when the likelihood was very low (**Error! Reference source not found.**). The data collection team underwent standardized training using a uniform disease classification protocol, implemented cross-auditing of observations between data collectors, and leveraged experienced personnel previously trained and validated in accurate BBTD identification across multiple countries.

Each of the 121 replanted plot was assessed for crop density and diversity within the replanted gardens, banana cultivar diversity, and BBTD incidence. In each target field, two diagonal transect lines were laid along which plants were sampled at regular intervals to cover all quarters of the plot. Every third

plant was assessed to determine BBTD incidence and aphid density, along two transverse transects, to cover all quarters of the garden. BBTD incidence was recorded by visually counting the symptomatic plants, which served as the primary response variable (Dato et al., 2021). BBTV is a systemic virus (once a single shoot is infected, the virus spreads to all other shoots in the mat (Djailo et al., 2016))). The unit of assessment was the banana mat, which included all shoots connected via the rhizome. The three youngest fully opened leaves per plant/shoot and three shoots of different sizes per mat were examined for BBTD symptoms on the lamina, petiole, and midrib following established protocols (Niyongere et al., 2013; Omondi et al., 2020; 2023). A mat was marked as diseased if any of its shoots exhibited any symptoms. The number of symptomatic mats was used as a proxy for the number of BBTV-infected plants. Plot owners were also interviewed to gather information on the field history, management practices during replanting, cultivar selection, source of planting material, and interaction with neighbouring farms. Social factors (gender of the plot owner and farm size) and management practices (varietal diversity, seed sourcing, rogueing frequency, use of banana-free fallow, and buffer zone maintenance) were assessed. A 90-meter area around the plot, divided into three concentric bands of 30 m each, was scouted for banana and BBTD presence and barriers to vector movement. Data were collected within both the focal plot and its buffer to evaluate the host distribution (banana density, crop composition, and vegetation diversity) and location characteristics (altitude and topography). A summary of all the variables assessed is presented in **Error! Reference source not found.**.

c. Data exploration

The dataset contained missing values that were imputed using the multiple imputation by chained equations (MICE) method from the *mice()* package for categorical variables and medians for continuous variables to enable analysis (Azur et al., 2011). To assess potential bias from imputation, we examined the standard errors of the imputed values. The dependent variable of interest was the reported number of mats with BBTD symptoms per plot, with each representing an infection event. Accordingly, count regression (Poisson and negative binomial) models were the most appropriate for assessing the statistically significant factors associated with banana BBTV infection risk. To select the

best count model, the mean and variance of the number of diseased banana plants were used to test the overdispersion of count variables. This was followed by an analysis of the number of diseased banana plants per plot.

The response variable showed a positively skewed distribution (skewness=1.9) and extremely different values for the unconditional mean (12.52) and variance (110.02), which had a nonlinear relationship, suggesting an overdispersion **(Error! Reference source not found.)**. The log-likelihood ratio test for the significance of overdispersion is computed as follows:

$$Log - likelyhood\ ratio\ = -2\ \times\ (log - likelihood\ of\ Poisson\ regression\ - log - likelihood\ of\ negative\ binomial\ regression)$$

The value of the likelihood-ratio test showed that the data were significantly over-dispersed ($\chi 2$ = 256.72, $p < 0.0001$), thus favouring the negative binomial regression model (NBRM), a generalization of the Poisson model. The NBRM permits sufficient flexibility with only two parameters (the shape parameter and mean). It subsumes the Poisson distribution, while allowing for overdispersion, where the variance may be greater than the mean.

To evaluate potential spatial autocorrelation in the dataset, we conducted a Local Indicators of Spatial Association (LISA) analysis alongside a Global Moran's I test, using infected plant counts from all surveyed locations. A spatial weights matrix was constructed using a 10 km distance threshold to define local neighbourhoods. The Global Moran's I test produced a Moran I statistic of -0.0212 (SD: -0.588; p a ≤ 0.722), indicating no significant global spatial autocorrelation in the infected plant counts. The Local Moran's I statistics and Z-scores were computed for each location to assess clustering or dispersion at a local scale. Locations were classified based on significance thresholds, and all showed no evidence of significant spatial clustering or dispersion. Taken together, these results demonstrate

the absence of meaningful spatial autocorrelation in the dataset. We therefore employed negative binomial models, variance inflation factors (VIFs), and model selection via AIC weighting given the structure of our dataset, considering the absence of significant spatial autocorrelation.

d. Model fitting

To model the factors affecting the number of plants affected by BBTD, a negative binomial generalized linear model (NB-GLM) was used to fit the global model, which included all the variables (**Error! Reference source not found.**, Supplementary Table 1), followed by model selection and model averaging. The global model was constructed to include all hypothesized predictors, ensuring the exploration of a comprehensive model space.

e. Multicollinearity checking

To ensure the stability and interpretability of the regression model, potential linear dependencies between the predictor variables in the global model were assessed using the *alias()* function from the *stats* package (version 4.3.2) in R version 4.4.3 (R Core Team (2025). This feature was used to identify the precise linear relationships within the design matrix. It detects predictors that can be expressed as exact linear combinations of others, thereby indicating multicollinearity problems. Identifying such dependencies ensures that the model parameters are clearly identifiable and interpretable, thereby avoiding estimation problems or misleading conclusions.

Variance inflation factors (VIF) were then calculated using the *vif()* function from the package *car* (version 3.1-2) to assess multicollinearity between the predictors. This function provides generalized variance inflation factor (GVIF) values along with degrees of freedom (df) and adjusted generalized variance inflation factor $\boldsymbol{GVIF}^{\left(\frac{1}{2\times Df}\right)}$ values for each variable. Adjustment was performed to make the GVIF values comparable across the variables with different degrees of freedom. Indeed, for some variables, the associated degrees of freedom are greater than one, indicating that they are categorical

variables with multiple levels. Variables with high (>5) adjusted GVIFs were discarded to ensure that there was no serious multicollinearity that could affect the stability of regression estimates (Siril et al., 2024). **Error! Reference source not found.** summarizes the variables retained after the multicollinearity check.

f. Model selection and averaging: assessment of best combinations of descriptors.

Effective disease management in agricultural systems is typically achieved through a combination of crop management strategies, which vary depending on the knowledge, experience, and resource availability of individual farmers. Understanding how different management practices interact to influence disease dynamics is essential to develop effective and scalable interventions. We aimed to simulate a hypothetical integrated management regime by identifying combinations of explanatory variables that best accounted for the observed variation in BBTD levels. We employed an information-theoretic approach to model selection by comparing all possible subsets of predictor variables derived from a global model using the corrected Akaike Information Criterion (AICc) (Cavanaugh and Neath, 2019). This approach allows for the identification of the most parsimonious models that adequately explain the variation in the response variable while penalizing the model complexity, particularly when working with relatively small sample sizes. Model selection was implemented using the *dredge()* function of the *MuMIn* R package (version 1.47.5), which automated the process of fitting and ranking all potential subset models. This function evaluates each candidate model by computing its AICc score, thus facilitating a comprehensive and unbiased assessment of variable importance. Therefore, we systematically explored the influence of different combinations of management practices on BBTD infection, ultimately guiding the identification of the most promising strategies for integrated disease control.

The Akaike Information Criterion (AIC) is a measure of model quality relative to other models, balancing model fit and complexity. The formula for AIC is:

$$\boldsymbol{AIC = -2lo{:}\,L \quad + 2k}$$

Where:

$\boldsymbol{L}$ = likelihood of data given for each fitted model

$\boldsymbol{k}$ = number of estimated parameters, including intercept.

For small sample sizes, AICc adjusts for potential overfitting by adding a correction term as follows:

$$\boldsymbol{AICc = AIC + \frac{2k(k+1)}{n-k-1}}$$

Where:

$\boldsymbol{n}$ = sample size, and $\boldsymbol{k}$ is defined above.

This adjustment becomes significant when $\boldsymbol{n}$ is small relative to $\boldsymbol{k}$. By penalizing models as the number of parameters approaches the sample size, AICc ensures a more conservative selection that protects against overfitting (Burnham and Anderson, 1998).

Parallel computing was utilized to enhance computational efficiency during model selection and validation. The *dredge()* function is parallelized across multiple cluster workers, allowing faster exploration of the model space. The candidate models generated by the *dredge()* function were ranked according to their AICc values, with the model exhibiting the lowest AICc considered the best-supported model. Model averaging was then performed using *avg()* function to account for model uncertainty. Models within the two AICc units of the best model were considered to have substantial support. This allowed for robust model comparison while balancing parsimony and predictive accuracy (Portet, 2020). The subset of models for averaging was selected by computing, for each model, $\Delta\boldsymbol{AICc}$ which is the difference in AICc with respect to the AICc of the best candidate model.

$$\Delta \boldsymbol{AICc = AICc_i - \; AIC_{min}}$$

Using a subset of models with $\Delta AICc$ < 2, the average coefficients and their standard errors were calculated. The $\Delta AICc$ values were also used to calculate the Akaike weight w_i of each model and the relative importance of the predictor variables appearing in the final model, using the following formula:

$$w_i = \frac{exp\left(-\frac{1}{2}\Delta AICc_i\right)}{\sum_{r=1}^{R} exp\left(-\frac{1}{2}\Delta AICc_r\right)}$$

where R is the number of models and r is the model being considered.

Note that if one of the models must be the best model, then w_i is the weight of evidence that a model is in fact the best model for the data and that all the models in the candidate subset have w_i that add up to 1. Finally, variable importance was assessed by calculating the relative importance values derived from the model-averaged coefficients (**Error! Reference source not found.**).

g. Evaluation of model performance

Although the AIC is widely used to identify the most parsimonious model among a set of candidates, it does not inherently measure how well a selected model fits observed data. Therefore, after selecting the best model based on AIC, we performed additional evaluations to assess its predictive adequacy. We also calculated the root-mean-square error (RMSE) and mean absolute error (MAE) as quantitative measures of prediction error. These diagnostics helped to assess whether the model captured the underlying data structure without systematic bias.

4. Results

The full model space, derived from all possible combinations of predictors in the global model, comprised 2,097,152 candidate models. Among these, Model 631,309 had the lowest AICc value (836.82), indicating that it was the most parsimonious model for the dataset. This model was used for the initial inference, although it does not account for model uncertainty because it is based on a single

best-fitting model. To address the model uncertainty, we employed model averaging. The averaged model coefficients (Table 4) are weighted across a subset of models with substantial support (ΔAICc < 2), providing more robust parameter estimates by integrating model uncertainty. This subset included 30 additional models that substantially contributed to the identification of the important predictors (Table 6). For ease of reference, these models were re-indexed from M1 to M31, with M1 representing the top-ranked model.

Model M1, which received the highest support among the subsets (accounting for 6% of the cumulative Akaike weight), identified several significant predictors of the number of BBTD-affected plants at the 5% significance level. These included plot size (m^2), type of planting material (tissue culture plantlets versus suckers), mean banana density 30-60m, presence of vegetation edges towards the east, village/location, and whether farmers used alternative seed sources not provided by the project. These variables relate globally to clean seed systems and manipulation of the abundance of vector or their behaviour (movement into gardens). Therefore, these variables should be included in future regression or modeling efforts aimed at understanding or managing BBTD.

Model M2, the second-best model, included all predictors in M1, except for banana density within a 30–60-meter radius, resulting in a model with fewer parameters. Models M2–M31 varied in the combination of included variables, often omitting some significant predictors or including others, but all had higher AICc values than M1. These differences primarily stem from the number of parameters estimated and reflected in the varying degrees of freedom. Approximately half of the models in the subset had between 9 and 11 degrees of freedom, with additional parameters contributing little explanatory power while increasing model complexity (Snipes and Taylor, 2014). This added complexity reduces the model quality by increasing the number of coefficients to be estimated without improving the fit. **Error! Reference source not found.** illustrates the relative importance of each variable across the retained models. Higher values indicate a greater likelihood of a given variable appearing in the best supported models. Overall, the best-performing models for BBTD management

aligned with the four main crop management strategies are listed in descending order of representation: (1) context-specific factors, such as garden location and plot size (28%), (2) seed system characteristics (26%), (3) banana density around the farm (16%) and vegetation barriers (10%), and (4) vector density (10%) and disease inoculum management (3%).

The best model (Table 4) included statistically significant predictors; however, several exhibited small effect sizes or wide confidence intervals, suggesting limited practical relevance. Residual plots revealed heteroscedasticity-nonconstant variance across residuals, indicating that the selected model did not fully capture all patterns in the data. This non-random structure in the residuals suggests that the model may overlook the nonlinear relationships or interactions among the variables. This also implies that the dataset may not be sufficiently robust to define the precise maximum effect sizes. Additionally, variations in the effectiveness of BBTD control measures may be influenced by context-specific environmental factors, such as altitude, temperature, and seasonal conditions, which were not fully accounted for in the model. Mean Absolute Error (MAE) and Root Mean Square Error (RMSE) are both metrics that quantify the difference between predicted values and observed values in the regression. MAE = 6.325 indicates that, on average, the predictions are off by approximately 6.33 units. The fact that the RMSE (8.757) was higher than the MAE indicates that there were some larger errors in the prediction.

5. Discussion

This study examined the field management and landscape-level factors associated with Banana Bunchy Top Disease (BBTD) infection in an endemic zone in Eastern Africa. We assessed variables related to field conditions within and surrounding smallholder banana gardens, cropping systems, vector abundance, disease prevalence, seed sourcing, and crop management practices and evaluated their association with BBTD occurrence in target plots. The project planting materials supplied to farmers

were certified plantlets from a tissue culture laboratory acclimatized in an insect-screened nursery within the village. Once planted, subsequent management decisions were independently made by farmers trained in BBTD management. This starting point–comparable production environments with differing management trajectories - allowed us to assess ecologically grounded management strategies. To the best of our knowledge, this is the first landscape-scale assessment of smallholder practices and their influence on BBTD dynamics from a known starting point. This offers insights into adaptable approaches for disease mitigation in similar agroecological settings. However, our analysis provides valuable insights into the shortcomings of some aspects of data availability. For example, the widest confidence interval ranges for the IRR were observed for the mean banana density at 30 m and 60 m. Designing an experimental protocol that captures greater variability (i.e., distance between rows and columns) could improve the practical utility and predictive power of the models for informing BBTD management strategies.

Our findings indicate that farm, buffer and landscape level diversity and landscape-scale farm crop management practices play an important role in BBTD control. Lower infection levels were associated with factors such as reduced banana density in surrounding fields, lower vector abundance, presence of vegetation barriers, seed source reliability, and more frequent field management. Notably, BBTD levels in neighboring gardens were not among the strongest predictors of infection in the target plots. This emphasizes the importance of broader community-scale management of isolated field-level interventions.

Landscape level complexity provides a relevant opportunity for the management of BBTD on a topographic scale. Banana canopy density around the recovered plot (connectivity between target plot and neighbouring plots) were associated with an increased BBTD risk in the target plot. These variables could influence the spread of vector aphids in the BBTD between mats and gardens (Kebede et al., 2018). Behavioural manipulation of insect pests and vectors through the intervention of non-host species is a powerful ecological management approach (Zhang et al., 2013). The availability of viable

BBTV susceptible hosts and the presence of backyard gardens could influence vector dispersal and virus spread dynamics. Over the past 50 years, banana-free periods and adequate buffer zones have been the most considered BBTD management options (Allen, 1978). We observed that hedges around banana gardens were associated with lower levels of infection, suggesting that vector immigration from external inoculum sources played a role in reinfection, contradicting the assumptions of Smith et al. (1998). Banana density in the buffer zone within 60 metres of the target garden was also important for predicting reinfection. Both non-host hedges and buffer zones minimize banana aphid migration into the target plot, thereby, reducing primary infection. The recommendation of large buffer zones to protect production gardens from BBTD reintroduction is not practical in smallholder systems with a high density of farmers, as it requires large land sizes for implementation (Omondi et al., 2023). A landscape-level community strategy incorporating vegetation barriers and a buffer of at least 40-60m which shifts outward as recovery advances may be more practical. Banana aphids have a restricted host spectrum and are directly affected by host distribution and BBTV (Watanabe et al., 2013, Wessels et al., 2019). Planning cropping mixtures at the landscape level would fit well with community approaches to BBTD management, especially when the choice of the most effective hedge species was identified. Therefore, selecting effective hedgerow species and incorporating vegetation barriers could enhance community-level BBTD control.

The role of infected asymptomatic banana mats remains unclear in our study, as only diseased plants were included. The distinction between infection, infectivity, and symptom expression, which is well documented in cavendish systems (Allen, 1978), was not made in this study. Incubation time, and hence the onset of symptom expression, varies across cultivars and may also be influenced by the physiological conditions of the infected plant. These factors could affect the detection and management of BBTD (Ngatat et al., 2022; Chabi et al, 2023). Asymptomatic banana mats could influence the model if such plants are significantly infectious (Cunniffe et al., 2012). The relationship between infection, infectiveness, and symptoms has been studied in BBTD, mostly in cavendish systems (Allen, 1978).

A delay between the appearance of the first symptom and infectivity was found in Cavendish and could also vary among banana cultivars (Ngatat et al., 2022). Although our study did not distinguish between infected, diseased, and infectious plants in the fields studied, Chabi et al., (2023) had found infectiousness of plants to be closely related to symptom appearance. As disease management is not uniform among the farm households studied, realistic estimates are difficult to obtain. However, all asymptomatic plants sampled were negative for BBTV when tested using ELISA. Similarly, disease progress has been shown to vary among cultivars (Ngatat et al., 2017), seasons (Niyongere et al., 2011) and can vary according to the altitude and age of crops (Leclerc et al., 2014).

Seed-sourcing practices are key determinants of BBTD risk and remain the most important route for BBTD introduction into new landscapes (Stainton et a., 2015). Farmer interest in new cultivars and cultivar diversity is an important route for the movement of seed of undeclared quality over longer distances. In this study, for example. five tissue-cultured cultivars were distributed under the project, with each farmer receiving up to three cultivars. and Additional cultivars were found in many gardens, indicating that farmers sourced extra seeds from elsewhere either to boost total plants planted or to include cultivars not multiplied by the project. Farmers who sourced planting material from informal or local sources were more likely to experience reinfection, highlighting the vulnerability of informal seed systems to seed-borne pathogens such as BBTV. Despite these risks, such informal sources remain important to farmers as they are the only route for the provision of locally preferred cultivars unavailable in formal seed systems (Simbare et al., 2020). These sourcing decisions often reflect farmers' cultivar preferences, perceived disease risks, and the use of relatively low-risk seed options as they lack mechanisms for verifying seed health (Nduwimana et al., 2022). Managing seed health, accompanied with measures to limit reinfection in the field is a key component of BBTD management (Mapinda et al., 2025). Strengthening clean seed systems in BBTD-affected areas poses logistical and social challenges, including limited availability of preferred varieties and gender-based barriers to access (McEwan et al., 2021). Practical solutions will require innovative, adaptable systems that align with farmers' demands, while ensuring seed health standards.

The removal of diseased bananas within and around the target plot was negatively associated with the infection rate in the target plot. Roguing can significantly reduce BBTV infection rates, lowering incidence to 2-10% when applied consistently and early in the disease expression stage (Omondi et al., 2020). However, the emergence of new infections in both managed and non-managed farms followed a seasonal cycle (Omondi et al., 2020), highlighting the need for time-series studies to clarify how roguing frequency, disease pressure, and environmental factors interact to shape long-term BBTD dynamics. The longer the diseased plants remain in the field, the higher is the probability of BBTV transmission to new plants in the field or landscape. Although our study did not directly assess roguing frequency owing to its cross-sectional design, its indirect effects may be inferred through plant mortality or farmer-reported roguing intensity. The relationship between roguing and infection is complex: higher reinfection rates may lead to increased roguing, whereas effective roguing may reduce infection. Time-series studies are needed to disentangle these dynamics and better quantify roguing efficacy.

Ecological interactions between the virus, vector, banana host, and other possible alternative host plants were relevant to this study. We did not assess the role of alternative hosts of BBTV or banana aphids in BBTD. To date, BBTV has not been shown to infect other Zingiberales species grown in the region, but the virus has been reported in other species, while the non-banana vector *P. caladii* has also been observed in bananas (Wessels et al., 2016; Mendoza et al., 2024) and is known to be a competent vector of BBTV (Watanabe et al., 2013). Both *P. caladii* and *P. nigronervosa* can complete their life cycles on several hosts other than banana, although *P. caladii* has a broader host range, while *P. nigronervosa* is largely restricted to *Musa* spp. (Foottit, 2010; Wessels et al., 2015). Despite both being competent BBTV vectors, *P. nigronervosa* is the more efficient transmitter. In bananas, host susceptibility and symptom expression likely contribute to disease dynamics, with evidence suggesting cultivar-level variations in BBTV accumulation and symptom severity (Niyongere et al., 2011; Ngatat et al., 2017;

2022; Chabi et al., 2023). Aphid colonization and reproduction may also vary by cultivar, influencing both vector population and transmission efficiency. Additionally, BBTV infection can alter the host preference of non-viruliferous *P. nigronervosa*, an effect lost once the aphid acquires the virus (Murhubaba et al., 2023; 2024), suggesting virus manipulation that could enhance transmission in endemic landscapes (Ingwell et al., 2012). Although this study assumed broadly similar weather conditions across geographically proximate study sites, future work should explicitly examine the influence of climatic variation as well as other factors, such as plot size, socioeconomic status, and seed exchange networks, which are known to intersect with gender and resource access (Nkengla et al., 2020; Nduwimana et al., 2022). A better understanding of these interactions could provide insight into epidemic development and improve predictive models of BBTD spread.

Our findings provide insight into the practical aspects of BBTD management. First, seed systems determine the introduction of BBTV into new gardens and landscapes and should be managed especially using indexed planting material on the one hand; and an understanding of important landraces to include in clean seed multiplication systems. Our observation on the protection of seed health agrees with modelling projections of Mapinda et al., (2025). Landscape complexity seems to function through both limiting vector dispersal and augmentative biological control through generalist predators. Generalist natural enemies may play an important role in keeping aphid populations low and preventing population build-up, which triggers dispersal. They also accelerate nymphal mortality, reducing the time spent on the host plant to acquire and inoculate the virus (Roudine et al., 2023). Studies on the specific dimensions of these correlates are important to determine their potential to improve a farmer-friendly package for BBTD management.

The findings from the model assessment against field data highlight the error risk when interpreting outputs from a single “best” model, even a model selected using AIC. Given the observed model limitations and presence of residual variance patterns (Figure 4), we advocate model averaging as a more reliable strategy. The averaged model (Table 3), which integrates the estimates from multiple

models weighted by their AIC support, provides a broader and more stable inference framework. Also, both the full and conditional model-averaged coefficients incorporate uncertainty across the model space, reducing the risk of overconfidence in any single model estimate. This approach is especially valuable in ecological and epidemiological studies where model uncertainty is substantial (Young, 2018). Moreover, the context-dependent nature of the BBTD spread suggests that some predictors may be more relevant in specific agroecological zones than others. For example, if aphid migration is the dominant transmission route in a given area, as opposed to ant-assisted or wind-mediated dispersal, control strategies should be tailored accordingly. Field experiments targeting such context-specific factors by manipulating levels of cropping mixtures content, presence of non-host barriers or selective levels of aphid and ant control should be conducted to offer more precise insights and support adaptive management strategies for BBTD.

6. Conclusion

Overall, our results highlight the interaction of landscape characteristics and field practices in shaping the BBTD dynamics. Three key factors in BBTD re-introduction into clean fields were revealed landscape complexity, farmers' seed movement, and host density, which can be consistently manipulated to achieve sustainable control. The most important variables appearing in the 31 best models reveal the importance of seed systems, control of vector movement, and a general reduction of infection pressure within and around the recovered plot in the sustainable management of BBTD. The success of these approaches may vary significantly according to latitude (or location) and weather conditions. Thus, common farmer cropping practices, such as intercropping and strip cropping, can be used to retard BBTD spread. Similarly, seed acquisition practices and direct removal of BBTD-affected plants are essential and available to farmers. We recommend that research extends these findings by using dynamic longitudinal data. Additionally, a gender-sensitive evaluation of identified technologies and practices, considering both social and environmental contexts, is essential for developing inclusive and sustainably integrated BBTD management strategies. To the best of our knowledge, this is the first

study to evaluate the spatial implications of farmer choices on BBTD within local smallholder farmer gardens. Importantly, this underscores the need to consider contextual factors in epidemiological modelling and disease control efforts in Africa.

Acknowledgments

We are grateful to the 121 farmer partners of the Banana Bunchy Top Disease Management Project in Cibitoke, Province, Burundi, whose plots yielded the datasets for their cooperation and maintenance of the pilot study sites. We also thank the leadership of the communes for assisting with mobilization at the beginning of the project. We are grateful for the support from the Ministry of Agriculture, Department of Agricultural Extension, and Burundi Institute of Agricultural Sciences (Institut des Sciences Agronomiques du Burundi, ISABU) for coordinating the BBTD recovery and seed system projects in Burundi. We also thank Abraham Shemezimana, and Emile Nitunga for support in data collection; Marius Aza and Geoffrey Dato for their support in initial data analysis and Dr. Svetlana Gaidashova, Rwanda Agriculture Board for her useful comments, which helped improve the quality of this manuscript. This study was funded by the CGIAR Cross-cutting Research Program on Root Tubers and Bananas under Cluster 3.4 Banana virus diseases.

Ethical Statement

This study involved the observation of farmers' fields for cropping, landscape, and disease assessment. It did not involve human research; therefore, this Ethical Review was not deemed applicable.

Funding Statement

Funding for this work was supported by the CIGAR Research Program on Root Tubers and Bananas (RTB) for ABO, AS, EN, CS, and CN at the Alliance of Bioversity International and CIAT and ISABU Burundi. RK, ABO, and CS were also supported by a Gates Foundation Grant on BBTD in Nigeria and Benin [INV010652], while RR was supported by The Gates Foundation Grant

[INV070408]. Under these grant conditions of the Foundation, a Creative Commons Attribution 4.0 Generic License has already been assigned to the Author Accepted Manuscript version that might arise from this submission. Further funding at the time of ABO was supported by the Plant Health Initiative of the One-CGIAR, which is grateful for the support of CGIAR Trust Fund contributors (www.cgiar.org/funders).

Data Accessibility

The datasets supporting this article have been uploaded as a part of the Supplementary Material (Table S1).

Competing Interests

We have no competing interests.'

Authors' Contributions

Conceived the study and designed study tools: ABO, CS, CN; Collected data: IN, ABO; Data analysis and interpretation: ABO, DRT, RR; Writing original draft: ABO, DRT; IN, Editing and Critically revising the draft: RR, CN, CS, DRT IN.

Approving for submission – all authors

Data availability: The original data from this study have been submitted as supplementary material (Table S1), with the descriptors in Table 1.

References

Adheka JG, Dhed'a DB, Karamura, D., Blomme, G., Swennen, R., & de Langhe, E. (2018). The morphological diversity of plantain in the Democratic Republic of Congo. *Sci. Hortic.*, **234**, 126-133. Doi: 10.17660/ActaHortic.2018.1225.36

Allen RN, 1978. Epidemiological factors influencing the success of rogueing for the control of bunchy top disease of bananas in New South Wales. *Aust. J. Agric. Res.* **29**, 535–544. Doi: 10.1071/AR9780535

Azur MJ, Stuart EA, Frangakis C, and Leaf PJ, (2011). Multiple imputation by chained equations: what is it and how does it work? *MPR*, **20**(1), 40–49. DOI: 10.1002/mpr.329

Burnham KP, and Anderson DR, (1998) Model Selection and Inference: A Practical Information-Theoretic Approach. Springer-Verlag, New York. DOI: 10.1007/978-1-4757-2917-7

Cavanaugh JE, Neath AA, (2019) The Akaike information criterion: Background, derivation, properties, application, interpretation, and refinements. *Wires Comps. Stats*.; 11: e1460. Doi: 10.1002/ wics.1460.

Chabi M, Dassou AG, Adoukonou-Sagbadja H, Thomas J, Omondi AB. (2023). Variation in symptom development and infectivity of banana bunchy top disease among four cultivars of *Musa* sp. *Crops*, **3**(2):158-169. Doi: 10.3390/crops3020016.

Cunniffe NJ, Stutt RJOH, van den Bosch F and Gilligan CA, (2012). Time-dependent infectivity and flexible latent and infectious periods in compartmental models of plant disease. *Phytopathol.* **102**, 365–380. Doi: 10.1094/PHYTO-12-10-0338.

Dato, KMG, Dégbègni MR, Atchadé MN, Hounkonnou MN, and Omondi BA, (2021). Spatial parameters associated with the risk of banana bunchy top disease in smallholder systems. *PloS ONE*, **16**(12), e0260976. DOI: 10.1371/journal.pone.0260976.

Djailo BD, Lokana J, Ngama F, Nkosi BI, Blomme G (2016). Systemicity of banana bunchy top viral infection in the Kisangani region of the Democratic Republic of Congo. *Afr. J. Agric. Res* **11**, 527-32. DOI: 10.5897/ajar2015.10641.

Fainou M, Ewédjè E-E, Kifouli A, Gustave L, Djedatin G, Affokpon A, Baba-Moussa F, and Fatiou T, (2018). Diversity of local varieties of banana and plantain cultivated in Benin. *IJBC*. 10. 497-509. DOI: 10.5897/IJBC2018.1232.

FAOSTAT (2023) FAO statistical database. https://www.fao.org/faostat/en/#data/QC. Accessed 01, June 2023

Hulme P. (2009). Trade, transport and trouble: managing invasive species pathways in an era of globalization, *J Appl. Ecol*., 46, 10 – 18. DOI: 10.1111/j.1365-2664.2008.01600.x.

Ingwell LL, Eigenbrode SD, Bosque-Perez NA (2012). Plant viruses alter insect behaviour to enhance their spread. *Sci Rep*., 2, 578. Doi: 10.1038/srep00578.

Jacobsen K, Omondi BA, Almekinders C, Alvarez E, Blomme G, Dita M, Iskra-Caruana M-L, Ocimati W, Tinzaara W, Kumar PL, and Staver C. (2019) Seed Degeneration of Banana Planting Materials: Strategies for Improved Farmer Access to Healthy Seed. *Plant Pathol.* 68, 207–228. https://doi.org/10.1111/ppa.12958.

Karamura DA, (1999). Numerical taxonomic studies of the East African highland bananas (Musa spp. AAA-East Africa) in Uganda. PhD Thesis, University of Reading, INIBAP, Montpellier, France. 192p. https://hdl.handle.net/10568/104967.

Kebede Y, Baudron F, F., Bianchi F, and Tittonell P, (2018). Unpacking the push-pull system: Assessing the contribution of companion crops along a gradient of landscape complexity. *Agric. Ecosyst. Environ.*, **268**, 115-123. doi: 10.1016/j.agee.2018.09.012.

Kumar PL, Hanna R, Alabi OJ, Vangu GH, Naidu RA (2011). Banana bunchy top virus in sub-Saharan Africa: Investigations on virus distribution and diversity. *Virus Res.* **159**, 171–82. DOI: 10.1016/j.virusres.2011.04.021.

Kumar PL, Selvarajan R, Iskra-Caruana ML, Chabannes M, Hanna R, (2015). Biology, Etiology, and Control of Virus Diseases of Banana and Plantain. *Adv Virus Res*. **91**, 229-69. DOI: 10.1016/bs.aivir.2014.10.006.

Leclerc M, Dore´ T, Gilligan CA, Lucas P, Filipe JAN (2014) Estimating the Delay between Host Infection and Disease (Incubation Period) and Assessing Its significance to the Epidemiology of Plant Diseases. *PLoS ONE,* 9(1), e86568. Doi: 10.1371/journal.pone.0086568.

Lepoint PCE, Staver C, Ajambo S, Rietveld AM, Kumar LP, Hanna R, and Caruana M-L. (2014). Workshop report: strategic planning workshop on recovering Banana production in BBTD affected areas: community and farm household approaches. https://cgspace.cgiar.org/server/api/core/bitstreams/c158a76e-0f5d-4256-abc6-6fd1a0dc39d8/content

Lepoint, P. (2016) Recovering banana production in BBTD affected areas: Strengthening cross-site learning tools in epidemiology, gender and social relations, and participatory experimentation approaches. CGIAR Research Program on Roots, Tubers and Bananas (RTB). RTB End of Project Workshop Report 2016. DOI: 10.13140/RG.2.1.1039.6323

Mapinda JJ., Hugo AK, Shinzeh JK Edward S (2025). Modelling the effects of virus-resistant planting material on the transmission dynamics of banana bunchy top disease. *Sci Rep* **15**, 20010. https://doi.org/10.1038/s41598-025-94881-7

Mbo LF, Tchinda N, Lauriane A, Cros, D, Martin G, Ndiang Z, Houegban J, Bille N, Bell JM, and Achigan-Dako, E. (2023). Analysis of genetic diversity and agronomic variation in banana sub-populations for genomic selection under drought stress in Southern Benin. *Gene*. 859. 147210. 10.1016/j.gene.2023.147210.

McEwan MA, Almekinders CMJ, Jorge JL Andrade-Piedra JJL, Delaquis E, Garrett KA, Kumar LP, Mayanja S, Omondi BA, Rajendran S, and Thiele G (2021). Breaking through the 40% adoption ceiling: Mind the seed system gaps. A perspective on seed systems research for development in One CGIAR. *Outlook Agric*. 50 (1), 5–12 doi.org/10.1177/0030727021989346

Mendoza NAP, Mendoza JS, Thomas JE, Dela Cueva FM (2024). Alternative hosts of banana bunchy top virus in the Philippines and the first evidence of seed transmission of BBTV. *Front Plant Sci*. **15**:1467331. doi: 10.3389/fpls.2024.1467331

Meyerson LA and Mooney HA (2007) Invasive alien species in an era of globalization. *Front. Ecol. and Environ*. **5**, 199-208. DOI: 10.1890/1540-9295(2007)5[199:IASIAE]2.0.CO;2

Munhoz T, Vargas J, Teixeira L, Staver C, and Dita M, (2024). Fusarium Tropical Race 4 in Latin America and the Caribbean: status and global research advances towards disease management. Frontiers in *Plant Sci.***15**, 1397617.

Murhububa SI, Tougeron K, Bragard C, Fauconnier ML, Basengere BE, Masamba JW, Hance T (2021) Banana tree infected with banana bunchy top virus attracts *Pentalonia nigronervosa* aphids through increased volatile organic compounds emission. *J Chem Ecol*., **47**:755-767. Doi: 10.1007/s10886-021-01285-0.

Murhububa IS, Bragard C, Tougeron K, Hance T (2024) Preference of *Pentalonia nigronervosa* for infected banana plants tends to reverse after *Banana bunchy top virus* acquisition. *Sci Rep* **14**, 2993 (2024). https://doi.org/10.1038/s41598-024-53205-x

Nduwimana I, Sylla S, Xing Y, Simbare A, Niyongere C, Garett K, and Omondi BA, (2022) Banana seed exchange networks in Burundi - linking formal and informal systems. *Outlook Agric*., 15 p. DOI: 10.1177/00307270221103288.

Ngatat S, Hanna R, Lienou J, Ghogomu RT, Nguidang SPK, Enoh AC, Ndemba B, Korie, S, Fotso Kuate A, Nanga Nanga, S, Fiaboe KKM, and Kumar PL (2022). *Musa* germplasm A and B genomic composition differentially affects their susceptibility to Banana Bunchy Top Virus and its aphid vector, *Pentalonia nigronervosa*. *Plants*, **11**(9), 1206. https://doi.org/10.3390/plants11091206.

Ngatat S, Hanna, R, Kumar, PL, et al., 2017. Relative susceptibility of *Musa* genotypes to banana bunchy top disease in Cameroon and implication for disease management, *Crop Prot.* **101**, 116 – 122. DOI: 10.1016/j.cropro.2017.07.018.

Niyongere C, Ateka EA, Losenge T, Lepoint P, Blomme G, (2011). Screening *Musa* genotypes for banana bunchy top disease resistance in Burundi. *Acta Hortic*., **897**, 439–47. DOI: 10.17660/ActaHortic.2011.897.60.

Niyongere C, Losenge T, Ateka EM, Ntukamazina N, Ndayiragije P, Simbare A, Cimpaye P, Nintije P, Lepoint P, Blomme G (2013) Understanding banana bunchy top disease epidemiology in Burundi for an enhanced and integrated management approach: Epidemiology of BBTD in Burundi. *Plant Pathol*, **62**, 562–570. Doi:10.1111/j.1365-3059.2012.02676.x.

Nkengla-Asi L, Omondi BA, Che Simo V, Assam E, Ngatat S, and Boonabaana B, (2020). Gender dynamics in banana seed systems and impact on banana bunchy top disease recovery in Cameroon. *Outlook Agric.* **49** (3), 235 244 doi: 10.1177/0030727020918333.

Omondi BA, Caruna M-L, and Staver C. (2021). Leveraging ecological processes and knowledge to recover banana production in BBTD-affected areas in Sub-Saharan Africa: Guidelines and new scientific challenges. In: Atta-Krah, K., Chotte, J. L., Gascuel, C., Gitz, V., Hainzelin, E., Hubert, B., ... & Sinclair, F. (eds). Agroecological transformation for sustainable food systems. Insight on France-CGIAR research. Les dossiers d'Agropolis International, 26. Agropolis International, Montpellier, France. pp. 66-67. doi: 10.23708/fdi:010082500

Omondi BA, Soko MM, Chabi M, Nduwimana I, Adjalla CC, Athindehou F, Amoussou RR, Dato GK, Tachin MZ, Niyongere C, and Staver C, (2023). Tools for the management of the banana bunchy top disease in small holder systems. *Acta Hortic*. **1367**, 233-242 DOI: 10.17660/ActaHortic.2023.1367.27

Omondi, B. A., Soko, M. M., Nduwimana, I., Delano, R. T., Niyongere, C., Simbare, A., and Staver, C. (2020). The effectiveness of consistent roguing in managing banana bunchy top disease in smallholder production in Africa. *Plant Pathol*, **69**, 1754-1766. doi: 10.1111/ ppa.13253.

Pemsl D, Staver C, Creamer B, Abdoulaye T, Alene A, and& Rusike J, (2014). Results of a global online expert survey: Major constraints, opportunities and trends for banana production and marketing and priorities for future RTB banana research. RTB Working Paper 2014-2, https://hdl.handle.net/10568/83418

Portet, S (2020) A primer on model selection using the Akaike Information Criterion, *Infect. Dis. Model.*, **5**, 111-128. Doi: 10.1016/j.idm.2019.12.010.

R Core Team (2025). *R: A Language and Environment for Statistical Computing*. R Foundation for Statistical Computing, Vienna, Austria. https://www.R-project.org.

Roudine S, Le Lann C, Bouvaine S, Le Ralec A, van Baaren J, (2023) Can biological control be a strategy to control vector-borne plant viruses? *J Pest Sci.* **96**, 451–470 https://doi.org/10.1007/s10340-022-01587-0

Simbare A, Sane CAB, Nduwimana I, Niyongere C, and Omondi BA, (2020). Diminishing Farm Diversity of East African Highland Bananas in Banana Bunchy Top Disease Outbreak Areas of Burundi—The Effect of Both Disease and Control Approaches. *Sustainability* **12**, 7467. DOI: 10.3390/su12187467.

Siril H, Gitagno D, Kaaya S, Caputo M, Hirschhorn L, Nyamuhanga T, Mtei R, Festo C, Hawkins C. (2024) Generalized and COVID related anxiety as risk factors for health outcomes among adolescents with HIV during COVID-19 in Tanzania. *Res Sq* [Preprint]. 2024 Feb 16:rs.3.rs-3921926. DOI : 10.21203/rs.3.rs-3921926/v1. PMID: 38410463; PMCID: PMC10896391.

Smith MC, Holt J, Kenyon L. and Foot C (1998) Quantitative epidemiology of Banana Bunchy Top Virus, Disease and its control, *Plant Pathol*, **47**, 177–187. DOI: 10.1046/j.1365-3059.1998.00230.x.

Snipes, M. and Taylor, D. C. (2014): Model selection and Akaike Information Criteria: An example from wine ratings and prices, *Wine Econ. Policy*, **3**, (1), 3-9. Doi: 10.1016/j.wep.2014.03.001

Stainton, D, Martin, DP, Muhire, et al. (2015). The global distribution of Banana bunchy top virus reveals little evidence for frequent recent, human-mediated long distance dispersal events. *Virus Evol*. **1**, 1-16. DOI: 10.1093/ve/vev009.

Staver, C. (2019). Ecological intensification of banana production systems. In Achieving sustainable cultivation of tropical fruits (pp. 261-322). Burleigh Dodds Science Publishing.

Swennen R, Vuylsteke D, and Ortiz R, (1995). Phenotypic diversity and patterns of variation in West and Central African plantains (*Musa* spp., AAB group Musaceae). *Econ Bot*, **49**, 320-327. Doi: 10.1007/BF02862352.

Watanabe S, Greenwell AM, Bressan A, (2013). Localization, concentration, and transmission efficiency of banana bunchy top virus in four asexual lineages of *Pentalonia* aphids. *Viruses* **5**, 758–775. Accessed on 26 June 2015. DOI: 10.3390/v5020758.

Wessels N, Omondi AB, Kruger K and Jooste E (2019) Non-destructive DNA extraction from aphids: the application in virus - vector studies of Banana bunchy top virus (BBTV) *Eur. J Plant Pathol*, **153**, 571–582. DOI: 10.1007/s10658-018-1552-2.

Young C, (2018). Model uncertainty and the crisis in science. *Socius*, 4, DOI: 2378023117737206.

Zhang Z, Sun X, Luo Z, Gao Y, and Chen Z, (2013), The manipulation mechanism of "push–pull" habitat management strategy and advances in its application, *Acta Ecol. Sin.*, **33** (2): 94-101, DOI: 10.1016/j.chnaes.2013.01.005.

Tables

Table 1. Complete list of variables and codification used before variable selection[1].

Code	Variables	Range	Levels
PSm2	Plot size in m^2	4 - 15624	null
DNIM	Distance of Nearest BBTD infected banana mat (meters)	0.275 - 197	null
BD30m	Mean of Banana density 30m (Mats/m^2)	0.002 – 0.378	null
BD60m	Mean of Banana density 60m (#Mats/m^2)	0.002 – 0.180	null
BD90m	Mean of Banana density 90m (#Mats/m^2)	0.002 – 5.501	null
RPY	rogueing per year farmer declaration	0 – 72	null
BBTD[2]	Number of plants affected by BBTD	1 - 57	null
AAP	Aphids' presence on asymptomatic mother plant	null	Yes (1), No (0)
ASP	Aphids' presence on symptomatic mother plants	null	Yes (1), No (0)
CSAM	Size of colony on Asymptomatic mats	null	Small simple colony, Large simple colonies (Mixed colonies (apterous and alate))
EPe	Edge presence East	null	Yes (1), No (0)
Eps	Edge presence South	null	Yes (1), No (0)
EPw	Edge presence West	null	Yes (1), No (0)
EPn	Edge presence North	null	Yes (1), No (0)
SeedS	Seed sources	null	1-Did not receive other **seed**s, 2-received other seeds
Var	Varieties	null	one variety (1), two varieties (2), three varieties (3), four varieties (4), more than 5 varieties (5)
OG	Plot owner gender	null	Male (1), female (2)
OPM	Other planting material on the plot	null	Yes (1), No (0)
CC	Associated crop cycle	null	Perennial (1), Seasonal (2), both (3)
MFN	Never planting material received from neighbours' or unknown sources	null	Yes (1), No (0)
MT	Type of planting material	null	Suckers (1), TC (2), Suckers + TC (3)
SF	Frequency of scouting banana infected	null	Less than monthly (1); Once a month (2); Twice a month (3); Weekly (4); Twice a week (5); Thrice a week (6); Four times a week (7); > four times a week (8)
BFP	Banana-free period	null	Yes (1), No (0)
Loc	Location	null	Munyika (1), Rusagara (2), Mugina (3), Rugombo (4)

[1] See also Supplementary Table 1
[2] Dependent variable

Table 2. List of variables included in the analysis after the collinearity test

**Variables.**	**GVIF**	**Df**	**GVIF^(1/(2*Df))**
PSm2	3.12	1	1.76
DNIM	1.47	1	1.21
BD30m	2.44	1	1.56
BD60m	2.08	1	1.44
BD90m	1.19	1	1.09
RPY	1.84	1	1.36
AAP	1.43	1	1.19
ASP	1.34	1	1.16
CSAM	3.29	2	1.35
EpE	1.91	1	1.38
EpS	2.15	1	1.47
EpW	2.63	1	1.62
EpN	1.93	1	1.39
SeedS	1.66	1	1.29
Var	7.19	4	1.28
CC	1.90	2	1.17
MFN	1.52	1	1.23
MT	3.01	2	1.32
SF	10.65	7	1.18
BFP	1.44	1	1.20
Loc	6.67	3	1.37

GVIF: Generalized variance inflation factor; GVIF^(1/(2*Df)): Adjusted generalized variance inflation factor; Df: degrees of freedom

Variable names: **AAP** - Aphids' presence on asymptomatic mother plant, **ASP** - Aphids' presence on symptomatic mother plants, **BD30m** - Mean of Banana density 30m (#Mats/m^2) **BD60m** - Mean of Banana density 60m (#Mats/m^2) **EpE** - Edge presence South; **EpS** - Edge presence South; **EpW**, Edge presence West; **Loc** - Location/Commune/Village; **MFN** - Never planting material received or collected from neighbours unknown ; **MT** - Type of planting material; **PSm2** (Plot size in m^2) ; **RPY** - rogueing per year farmer declaration ; **SeedS** - Seed sources

Table 3. Model-Averaged Coefficients (Full and conditional averaging)

Table 4. Model-Averaged Coefficients (Full and conditional averaging)

	Full average model [a]				Conditional average model [b]			
	**Estimate**	**SE**	**IRR**	**p-value**	**Estimate**	**SE**	**IRR**	**p-value**
(Intercept)	1.832	0.306	6.246	0.000	1.830	0.306	6.234	0.000
BD30m	2.394	2.847	10.957	0.402	4.590	2.340	98.494	0.052
BD60m	3.676	3.450	39.488	0.289	5.320	2.910	204.384	0.071
EPe	0.320	0.144	1.377	0.028	0.320	0.144	1.377	0.028
Loc = Kagazi	-0.042	0.217	0.959	0.849	-0.042	0.217	0.959	0.849
Loc = Munyika	-0.623	0.210	0.536	0.003	-0.623	0.210	0.536	0.003
Loc = Rusagara	0.154	0.177	1.166	0.389	0.154	0.177	1.166	0.389
MT = TC	-1.378	1.066	0.252	0.197	-1.920	0.738	0.147	0.010
MT = Suckers + TC	0.029	0.122	1.030	0.813	0.041	0.142	1.041	0.778
PSm2	1.46e-04	3.66e-05	1.000	0.001	1.35e-4	3.86e-05	1.000	0.001
SeedS	0.364	0.141	1.439	0.010	0.364	0.141	1.439	0.010
RPY	0.001	0.003	1.001	0.680	0.006	0.004	1.006	0.146
AAP	0.166	0.233	1.181	0.478	0.351	0.223	1.420	0.120
ASP	-0.068	0.170	0.934	0.690	-0.318	0.235	0.728	0.181
MFN	-0.038	0.147	0.962	0.796	-0.362	0.295	0.696	0.225
Epw	-0.009	0.054	0.991	0.866	-0.175	0.162	0.839	0.284
Eps	-0.004	0.036	0.996	0.918	-0.151	0.173	0.860	0.389

IRR = Incidence Rate Ratios

[a] Includes all candidate models in the averaging process, regardless of whether a particular predictor is included in each model.

[b] Only includes models in which the predictor is present

AAP - Aphids' presence on asymptomatic mother plant, ASP - Aphids' presence on symptomatic mother plants, BD30m - Mean of Banana density 30m (#Mats/m^2) BD60m - Mean of Banana density 60m (#Mats/m^2) Epe - Edge presence South; Eps - Edge presence South; EPw, Edge presence West; Loc - Location/Commune/Village; MFN - Never planting material received or collected from neighbours unknown ; MT - Type of planting material; PSm2 (Plot size in m^2) ; RPY - rogueing per year farmer declaration ; SeedS - Seed sources

Table 5. Variable factors associated with changes in BBTD incidence

Predictors	Coefficients	IRR	CI	p-value
(Intercept)	1.819	6.17	4.26 – 8.99	<0.001
BD30m	4.468	87.17	0.79 – 10084.68	0.050
BD60m	4.990	146.91	0.50 – 50055.42	0.087
EPe	0.326	1.39	1.06 – 1.82	0.022
Loc = Kagazi	0.000	1.00	0.66 – 1.54	0.999
Loc = Munyika	-0.611	0.54	0.36 – 0.81	0.003
Loc = Rusagara	0.220	1.25	0.89 – 1.74	0.190
MT = TC	-2.160	0.12	0.03 – 0.49	0.002
MT = Suckers + TC	0.035	1.04	0.79 – 1.36	0.805
PSm2	1.46e-04	1.00	1.00 – 1.00	<0.001
SeedS = other seeds sources	0.319	1.38	1.06 – 1.79	0.017

IRR = Incidence Rate Ratios; CI = Confidence interval

AAP - Aphids' presence on asymptomatic mother plant, **ASP** - Aphids' presence on symptomatic mother plants, **BD30m** - Mean of Banana density 30m (#Mats/m^2) **BD60m** - Mean of Banana density 60m (#Mats/m^2) **EpE** - Edge presence South; **EpS** - Edge presence South; **EpW**, Edge presence West; **Loc** - Location/Commune/Village; **MFN** - Never planting material received or collected from neighbours unknown ; **MT** - Type of planting material; **PSm2** (Plot size in m^2) ; **RPY** - rogueing per year farmer declaration ; **SeedS** - Seed sources

Table 6. Summary of AICc results for models relating the BBTD control and the variables

**Models**		**df**	**L**	**AICc**	**ΔAICc**	w_i
**M1**	3+4+5+8+10+11+13	12	-404.97	836.82	0.00	0.06
**M2**	3+5+8+10+11+13	11	-406.43	837.29	0.46	0.05
**M3**	4+5+8+10+11+12+13	12	-405.25	837.39	0.57	0.04
**M4**	1+3+4+5+8+10+11+13	13	-404.00	837.39	0.57	0.04
**M5**	4+5+8+11+13	9	-408.96	837.53	0.71	0.04
**M6**	1+4+5+8+11+13	10	-407.77	837.54	0.72	0.04
**M7**	2+3+5+8+10+11+13	12	-405.34	837.58	0.75	0.04
**M8**	1+4+5+8+10+11+13	12	-405.45	837.79	0.97	0.04
**M9**	4+5+8+10+11+13	11	-406.69	837.80	0.98	0.04
**M10**	1+2+3+5+8+10+11+13	13	-404.20	837.80	0.98	0.04
**M11**	1+3+5+8+10+11+13	12	-405.49	837.88	1.05	0.04
**M12**	2+3+4+5+8+10+11+13	13	-404.25	837.90	1.08	0.03
**M13**	1+4+5+8+10+11+12+13	13	-404.32	838.03	1.21	0.03
**M14**	3+4+5+8+10+11+12+13	13	-404.34	838.08	1.25	0.03
**M15**	1+2+3+4+5+8+10+11+13	14	-403.11	838.18	1.35	0.03
**M16**	3+4+5+8+9+10+11+13	13	-404.40	838.21	1.39	0.03
**M17**	3+4+5+7+8+10+11+13	13	-404.41	838.22	1.40	0.03
**M18**	3+5+8+10+11+12+13	12	-405.69	838.27	1.45	0.03
**M19**	5+8+11+12+13	9	-409.33	838.28	1.46	0.03
**M20**	1+3+4+5+8+9+10+11+13	14	-403.21	838.39	1.56	0.03
**M21**	4+5+8+11+12+13	10	-408.22	838.44	1.62	0.03
**M22**	1+2+4+5+8+11+13	11	-407.04	838.50	1.68	0.03
**M23**	1+4+5+8+9+11+13	11	-407.05	838.52	1.70	0.03
**M24**	3+4+5+6+8+10+11+13	13	-404.59	838.58	1.75	0.02
**M25**	1+5+8+11+13	9	-409.50	838.62	1.80	0.02
**M26**	1+2+4+5+8+10+11+13	13	-404.63	838.65	1.83	0.02
**M27**	1+4+5+8+9+10+11+13	13	-404.64	838.68	1.86	0.02
**M28**	1+2+5+8+11+13	10	-408.35	838.70	1.87	0.02
**M29**	5+8+11+13	8	-410.73	838.75	1.93	0.02
**M30**	1+5+8+11+12+13	10	-408.38	838.76	1.93	0.02
**M31**	1+3+4+5+7+8+10+11+13	14	-403.40	838.77	1.94	0.02

w_i = Akaike weight of each model; L = Loglikelihood; AICc = Akaike Information Criterion corrected
ΔAICc = differences in AICc with respect to the AICc of the best candidate model, Df = degree of freedom
1 = Aphids' presence on asymptomatic mother plant (AAP), 2 = Aphids' presence on symptomatic mother plants (ASP), 3 = Mean of Banana density 30m (#Mats/m²) (BD30m,) 4 = Mean of Banana density 60m (#Mats/m²) (BD60m) 5 = Edge presence South (EPe); 6 = EPs, Edge presence South 7 = EPw, Edge presence West; 8 = Loc, Location/Commune/Village; 9 = MFN, Never planting material received or collected from neighbours unknown ; 10 = MT, Type of planting material ;11 = Plot size in m² (PSm2) ;12 = rogueing per year farmer declaration (RPY) 13 = Seed sources (SeedS)

Figure legends

Figure 1: Sketch of the plot assessment plan showing the target plot (green) and the areas assessed for vegetation characterization.

Figure 2: Histogram showing the density of the number of BBTD infected banana plants (for the count regression model)

Figure 3: Relative importance of selected variables based on the frequency of inclusion in the best combination of models.

Figure 4: Observed vs. Predicted values and residual diagnostics for best model fit. The symbol size corresponds to field size, filled colours represent location and symbol shape indicate seed system. These variables were chosen based on Figure 4, as they are the top three most important across all models.

Figure

Click here to access/download;Figure;Figure 1 Target plot and buffer rings.png

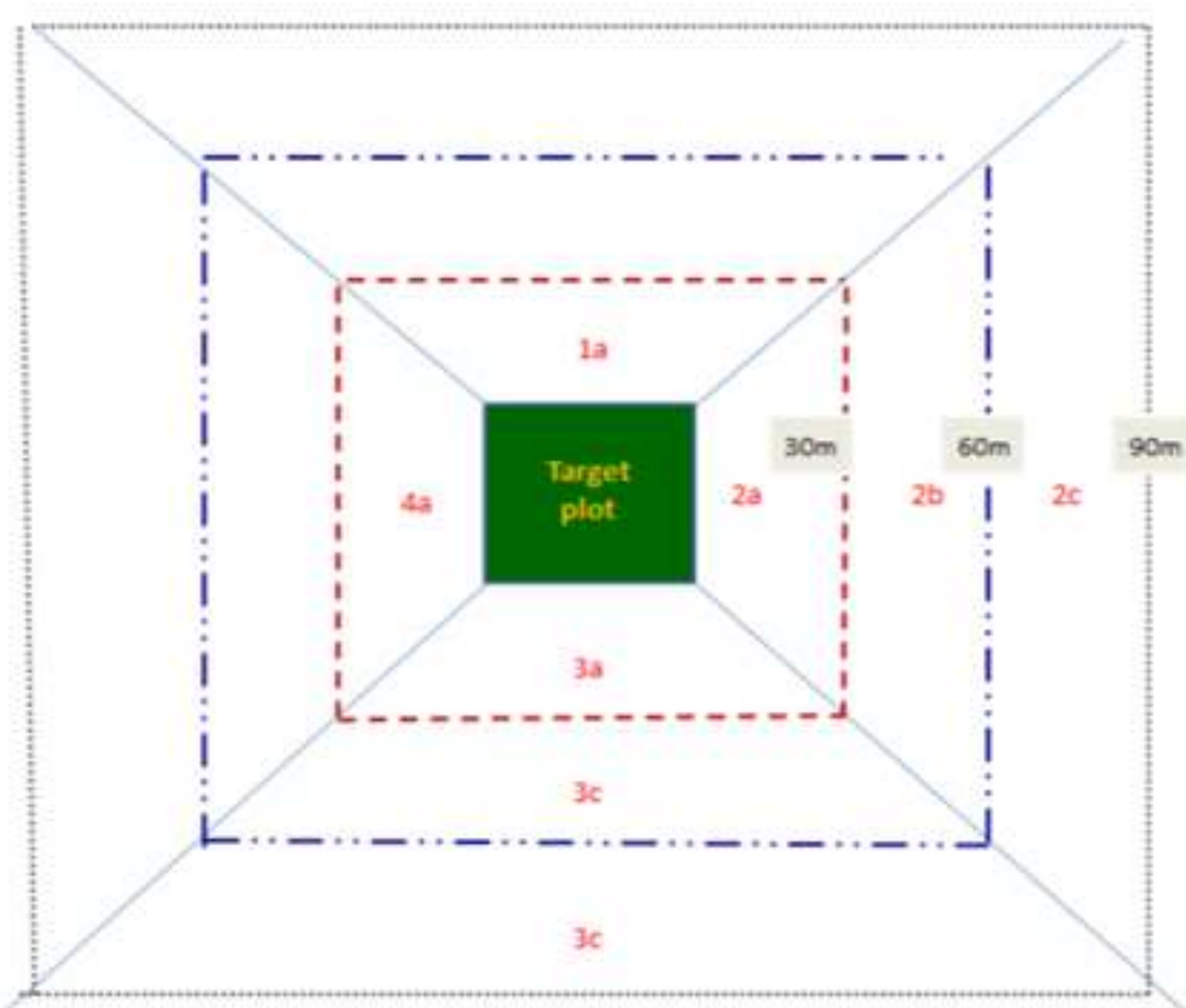

Figure

Click here to access/download;Figure;Figure 2 data evaluation skewness.png

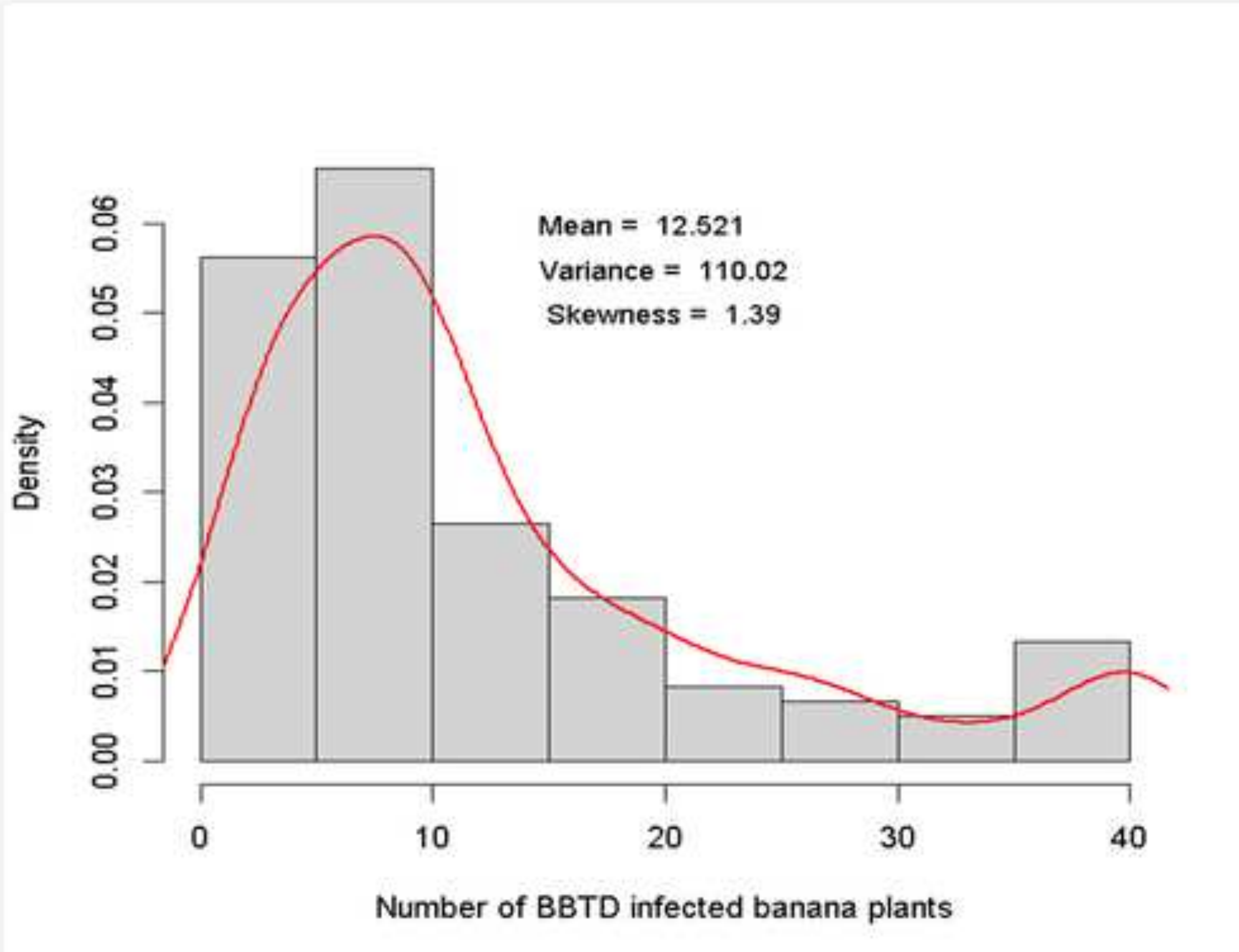

Figure

Click here to access/download;Figure;Figure 3 - relative importance of variable combinations.png

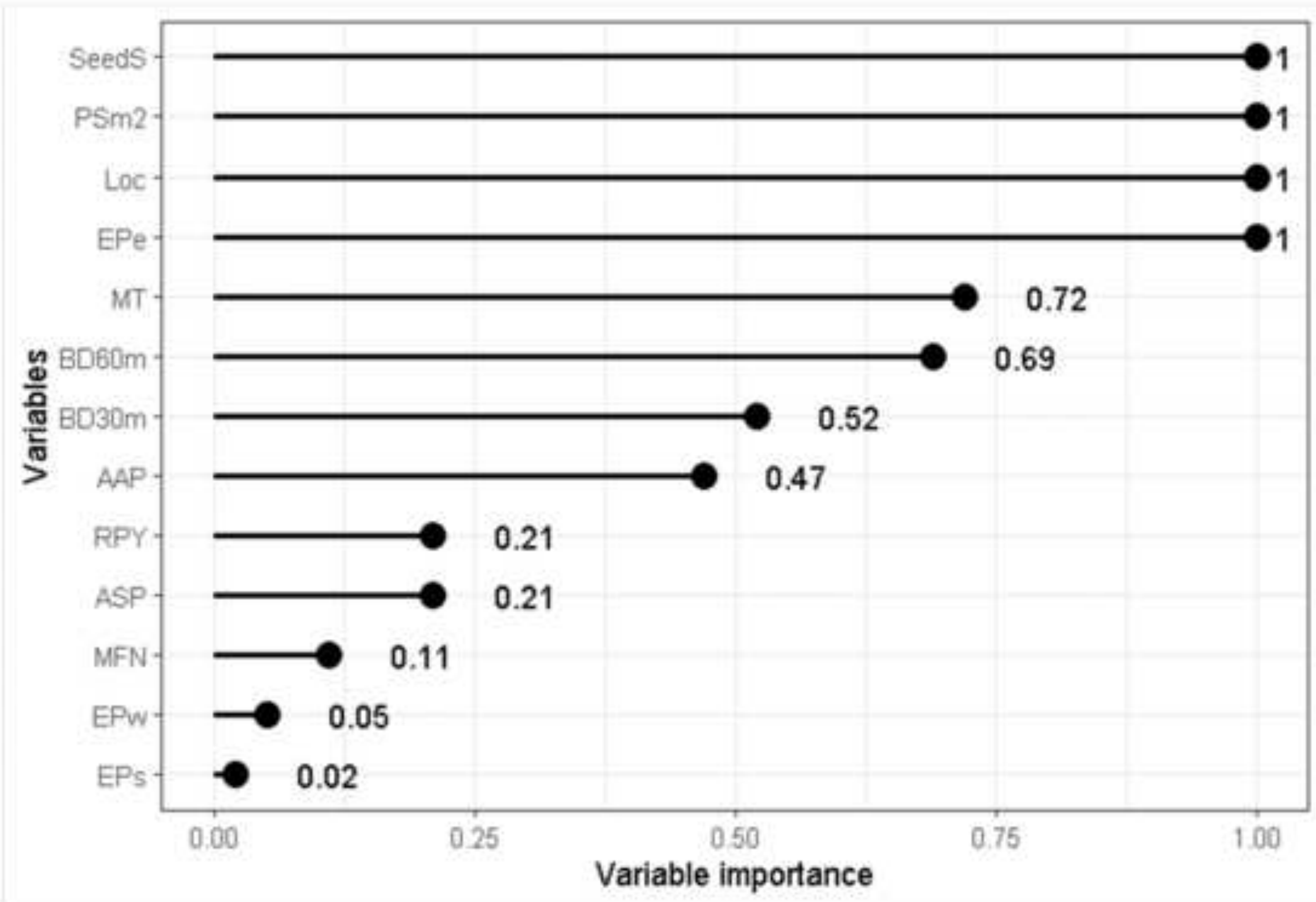

Figure

Click here to access/download;Figure;Figure 4 model assessment.png

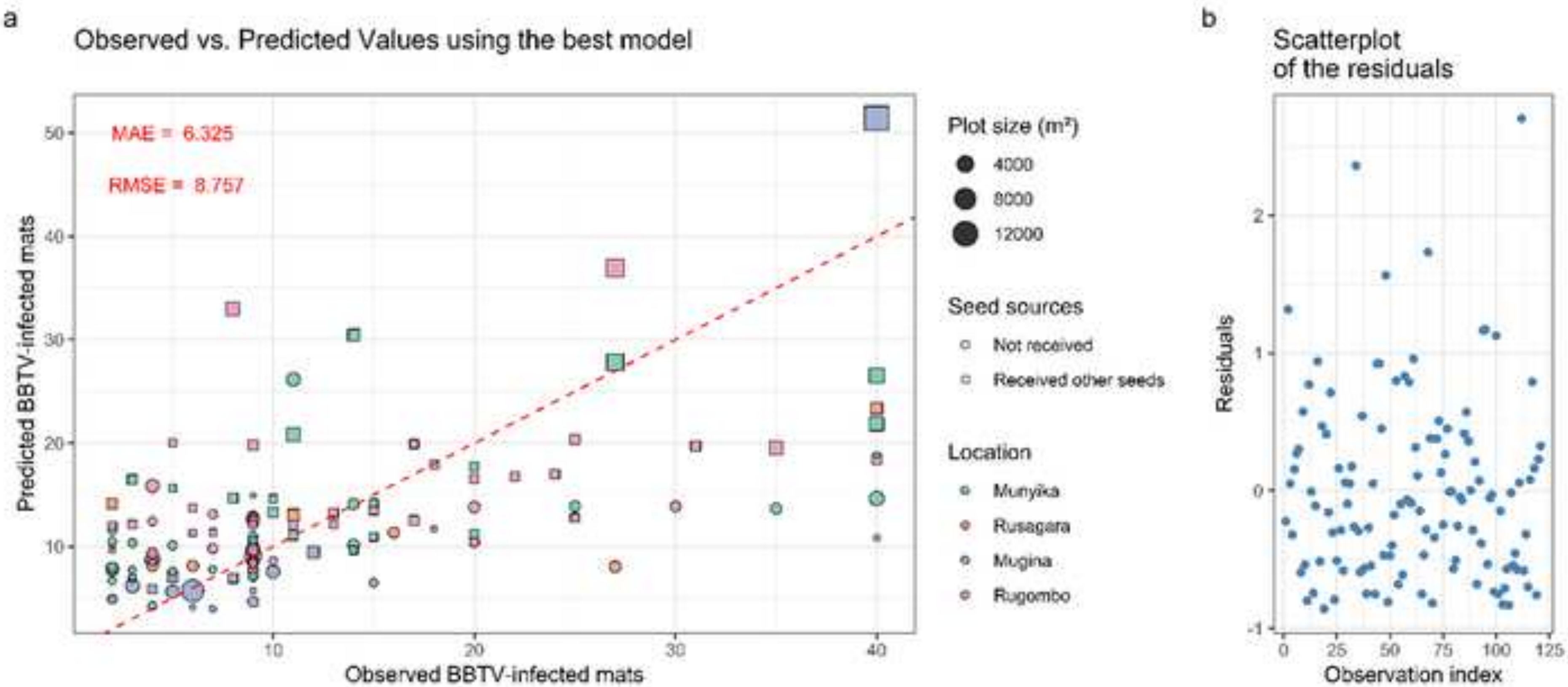

Click here to access/download
Supporting Information
Copy of BBTD_dataset_ok.xlsx

Drivers of Banana Re-infection in Newly Planted Gardens in Banana Bunch top Disease Endemic Zones: Implications for the recovery of banana production

Aman Bonaventure Omondi[1], Delano Ronald Togbé[2], Renata Retkute[3], Innocent Nduwimana[4], Niyongere Celestin[5,6], Staver Charles[7,8].

1. *Bioversity International, Alliance of Bioversity International and CIAT, c/o IPGRI Building, IITA Campus, Abomey-Calavi, 08BP 0932, Cotonou, Benin.*
2. *Faculty of Agronomic Sciences, University of Abomey-Calavi, Benin*
3. *Epidemiology and Modeling Group, Department of Plant Sciences, University of Cambridge, Downing Street, Cambridge CB2 3EA, UK*
4. *Bioversity International, The Alliance of Bioversity International and CIAT, co/ IITA Building, Regina Mundi Road, Bujumbura, Burundi.*
5. *Institute of Agronomic Sciences, (ISABU) Rohero 1, Avenue de la Cathédrale, B.P 795 Bujumbura, Burundi,*
6. *CAB International (CABI), Quartier Kabondo Ouest,10, Avenue du Large, PO Box 1995, Bujumbura, Burundi*
7. *Bioversity International, Parc Scientifique Agropolis 1990 bd Lironde, 34980 Montferrier sur Lez, Montpellier, France.*
8. *Faculty of Agricultural Sciences, Universidad Veracruzana, Xalapa, Mexico*

Authors' ORCID numbers
Aman Bonaventure Omondi - 0000-0001-9711-5645
Delano Ronald Togbé - 0000-0002-0080-8021
Renata Retkute: 0000-0002-3877-6440
Innocent Nduwimana - 0000-0001-7110-354X
Niyongere Celestin (n/a)
Charles Staver (CS): 0000-0002-4532-6077

*Author for correspondence: B.A. Omondi (b.a.omondi@cgiar.org).

†Present address: Bioversity International, Alliance of Bioversity International and CIAY, IITA Campus, c/o IPGRI Building, IITA Campus, Abomey Calavi, 08 BP 0932, Cotonou, Benin

1. **Abstract**

Understanding how crop pests and diseases spread within and between fields can inform the design of effective integrated pest and disease management strategies. This study aimed to identify the key landscape, field design, and management factors influencing the persistence and re-infection of Banana Bunchy Top Disease (BBTD) in endemic smallholder systems. We assessed 121 banana gardens reestablished with virus-free tissue-culture plantlets, three years prior, to determine the combination of practices associated with low BBTD incidence. Positive associations with disease prevalence included high banana density within a 60 m radius, elevated aphid populations, greater varietal diversity, and the use of locally sourced planting material. In contrast, higher altitude, the presence of hedgerows, and frequent rogueing were negatively associated with BBTD. Model selection indicated that aphid prevalence, banana density, rogueing frequency, and seed source appeared in more than half of the best-fit models, underscoring their importance in minimizing reinfection and yield loss. These findings emphasize the value of coordinated, community-based replanting with virus-free material, regular rogueing informed by farmer symptom recognition, and the integration of barrier hedges or mixed cropping to restrict aphid dispersal. Further research should investigate optimal species combinations and landscape configurations to enhance BBTD suppression in smallholder production systems.

Keywords: Banana Bunchy Top Disease, integrated pest and disease management, small holder farming, cropping mixtures, spatial epidemiology

2. Introduction

Invasive species represent a major threat to agricultural productivity and the global stability of ecosystems. Their proliferation, often driven by global trade, climate change, and human-mediated landscape alterations, disrupts local ecological balances and exacerbates crop loss (Meyerson & Mooney, 2007; Hulme, 2009). In agroecosystems, invasive pests and pathogens can spread rapidly, especially in landscapes with conducive habitats and susceptible hosts in the absence of natural enemies and without coordinated management. Many pests and diseases rapidly establish in new areas of incursion depending on host availability, dispersal mechanisms (including effective vectors), and spatial connectivity between suitable habitats across the landscape. Understanding how host distribution and landscape connectivity influence pest and disease dynamics is important for managing invasive plant pathogens and insect vectors which have become endemic. At the local scale, the spread of disease is influenced by field-level management practices, such as sanitation, crop diversity, and planting patterns, which affect the inoculum pressure and vector behaviour. Spatial modelling of these agro-environmental factors can help to identify ecological thresholds and intervention points for disease control.

In Sub-Saharan Africa, more than 50 types and cultivars of banana (including plantain, *Musa* spp.) are cultivated (Swennen et al., 1995; Karamura, 1999; Adheka et al., 2018, Fainou et al., 2018; Mbo et al., 2023), a vital staple and cash crop contributing to food security and income for millions of smallholder farmers (FAOSTAT 2023). As a perennial crop, banana supports year-round harvesting, which is especially important for rural households’ food and economic security. The banana fruit and various plant parts are consumed in multiple forms depending on the variety. Dessert bananas are eaten fresh as fruits, whereas cooking bananas and plantains serve as starchy staples or snacks. Some varieties are used to produce processed beverages, such as banana beer and juice, while others are considered multipurpose because of their suitability for several uses. This wide range of uses makes banana an

essential crop for food security, nutrition, and income generation, especially in smallholder farming systems across tropical regions. However, yields across Africa remain among the lowest globally, primarily due to a range of biotic stressors, including pests and diseases often transmitted in locally sourced seed and low land productivity with few external inputs (Jacobsen et al., 2019). The case of banana landscapes in Burundi illustrates the challenges of invasive diseases. Farm communities in the Rift Valley lost fields of the previously dominant beer banana variety, 'Kayinja', to Fusarium wilt race 1. They replaced 'Kayinja' with 'Yangambi Km5' only to face the proliferation of Banana Bunchy Top Disease (BBTD) (Simbare et al., 2020) which is the subject of this paper. Another Fusarium strain, Tropical Race 4 (TR4), has reached sub-Saharan Africa but is not yet widespread. Many cultivars across the region are susceptible to TR4 (Munhoz et al 2024). Sustainable management of these invasive species is therefore essential for securing food systems and livelihoods on the continent, especially among smallholder farmers.

Banana bunchy top disease, caused by the banana bunchy top virus (BBTV), is one of the most destructive viral diseases affecting bananas (Stainton et al., 2015). It is transmitted by the banana aphid (*Pentalonia nigronervosa*) and infected planting material. Once established, the disease causes a progressive yield loss. More seriously, the local supply of clean banana suckers diminishes as, progressively, more banana mats become infected by BBTD. Farmers lose access to BBTV-free planting material as local seed supply degenerates (Jacobsen et al 2019) and banana production collapses. Currently, BBTD has spread to at least 18 countries in Africa and is estimated to affect 6–12 million smallholder households (Kumar et al., 2011; 2015). In heavily affected regions such as Malawi and the Democratic Republic of the Congo (DRC), production losses have significantly undermined livelihoods (Kumar et al., 2011). Moreover, BBTD poses a serious threat to banana genetic diversity in two centres of secondary diversity of cultivated bananas: The East African Highlands (home to over 100 AAA-EA types) (Karamura, 1999; Adheka et al., 2018) and West Africa, the centre of plantain diversity (Fainou et al., 2018; Swennen et al., 1995). Such genetic loss may threaten the long-term sources of genetic material for banana improvement against other emergent biotic and

abiotic challenges. Small-holder production systems, dominated by local landraces, are particularly vulnerable because of their dependence on informal seed sourcing and the perennial low-input nature of the production system (Simbare et al., 2020).

In 2014, The Learning Alliance for BBTD control in Africa (https://www.bbtvalliance.org/), an alliance of international agricultural research centres, sub-Saharan national research organizations, and rural communities undertook banana production recovery efforts in BBTD-affected landscapes in Burundi and eight other countries using a community-based collaborative learning and action approach (Lepoint et al 2014, Lepoint et al 2016). This strategy involved landscape-scale destruction of potentially infected plants, enforcement of a banana-free fallow period to eliminate inoculum sources and viruliferous aphids, multiplication of disease-free tissue culture planting material, replanting in well-isolated fields, and collaborative monitoring of replanted gardens (Omondi et al., 2020; 2023). Effective implementation requires strong landscape-level coordination, particularly in crop monitoring, inoculum reduction by consistent roguing, and the adoption of clean seed supply systems (Omondi et al, 2021).

Understanding the risk of reinfection in recovered banana gardens affected by BBTD is an important component of long-term disease management and the recovery of banana production in BBTD-affected landscapes. Maintaining a low and stable reinfection risk allows for the gradual expansion of recovered areas, utilizing existing buffer zones and facilitating the growth of seed production and exchange systems within these zones. The recovery strategy deployed in Burundi offered a case study to monitor reinfection in 121 small farms and two community nurseries for tissue culture plants in four villages. Each farmer's field offered a suite of local factors, although the area itself was characterized by small mixed crop perennial banana gardens and backyard banana mats. At a distance, these landscapes resemble large plantations in terms of banana canopy continuity, but they are a complex mosaic of

diverse banana varieties, associated crops, and landscape features such as hedgerows, fallow fields, rice monocrops, and farming practices across an altitudinal gradient.

Our study aimed to identify the key risk factors associated with BBTD reinfection in replanted banana fields to improve the current banana recovery strategies. By assessing the prevalence of BBTD in farms that were replanted with BBTV-free tissue culture planting materials in 2013 and 2014, we aimed to evaluate the factors associated with disease transmission. A single assessment of the reinfection status of initially clean plots across farms with similar background risk provided a unique opportunity to evaluate the risk pressures of BBTD prevalence while minimizing the influence of temporal variability.

3. Materials and Methods

a. Study Area and Farm Selection

A field study was conducted on 121 farms across four communes— Kagazi, Rusagara, Munyika, and Gitebe—in Cibitoke Province, Burundi. Cibitoke Province is a major banana-growing region and a known hotspot for Banana Bunchy Top Disease (BBTD). It lies along the border between Burundi, Rwanda, and the Democratic Republic of Congo (DRC), spanning altitudes from 800 to 1300 m above sea level. Rusizi Valley, shared among these three countries, has been endemic to BBTD for over three decades. The spread of BBTV through the vector is favoured by environmental factors favouring vector aphid population build up and migration, and rapid plant growth and accumulation of BBTV titre to enable vector acquisition. These include favourable temperature, wind and topography (Niyongere et al., 2013). Thus, the greatest BBTD risk BBTD has been associated with humid lowlands. Pilot sites were established in selected communes in 2014 to evaluate community-based approaches to BBTD recovery (Omondi et al., 2020, 2023). The farms assessed in this study had been replanted using BBTV-free tissue culture (TC) plantlets sourced from a private tissue culture propagation laboratory (Agrobiotec Limited, Bujumbura, Burundi) and hardened in community-based nurseries. While

farmers were trained and encouraged to implement BBTD risk reduction and cultural management practices, implementation varied based on individual capacities and priorities. As all farms shared a known replanting start date with virus-free material, they provided a consistent baseline for assessing the environmental and management factors influencing BBTD reinfection. The presence of BBTD in the surrounding landscape ensured a comparable source of inoculum across sites, minimizing spatial bias that would otherwise occur in newly affected areas where infections remain localized in hotspots. By focusing on replanted fields, we eliminated the confounding effects of local disease history and topographic variation, allowing plot-level history and active management practices to emerge as the main explanatory factors

b. Data Collection

The data were collected using a combination of direct field measurements and structured surveys. A sketch map of each target garden was created to orient the characterization and data acquisition of the field itself and a 90m surrounding observational zone corresponding to the buffer proposed to reduce aphid movement to the field. Thus, each of the 121 gardens planted with tissue culture (TC) banana plantlets in the BBTD recovery effort three months prior, was treated as a focal plot. The buffer was divided into here zones, following Allen (1978), observation that aphid arrival to a field declined with distance from the field up to 90 m, when the likelihood was very low (**Error! Reference source not found.**). The data collection team underwent standardized training using a uniform disease classification protocol, implemented cross-auditing of observations between data collectors, and leveraged experienced personnel previously trained and validated in accurate BBTD identification across multiple countries.

Each of the 121 replanted plot was assessed for crop density and diversity within the replanted gardens, banana cultivar diversity, and BBTD incidence. In each target field, two diagonal transect lines were laid along which plants were sampled at regular intervals to cover all quarters of the plot. Every third

plant was assessed to determine BBTD incidence and aphid density, along two transverse transects, to cover all quarters of the garden. BBTD incidence was recorded by visually counting the symptomatic plants, which served as the primary response variable (Dato et al., 2021). BBTV is a systemic virus (once a single shoot is infected, the virus spreads to all other shoots in the mat (Djailo et al., 2016)). The unit of assessment was the banana mat, which included all shoots connected via the rhizome. The three youngest fully opened leaves per plant/shoot and three shoots of different sizes per mat were examined for BBTD symptoms on the lamina, petiole, and midrib following established protocols (Niyongere et al., 2013; Omondi et al., 2020; 2023). A mat was marked as diseased if any of its shoots exhibited any symptoms. The number of symptomatic mats was used as a proxy for the number of BBTV-infected plants. Plot owners were also interviewed to gather information on the field history, management practices during replanting, cultivar selection, source of planting material, and interaction with neighbouring farms. Social factors (gender of the plot owner and farm size) and management practices (varietal diversity, seed sourcing, rogueing frequency, use of banana-free fallow, and buffer zone maintenance) were assessed. A 90-meter area around the plot, divided into three concentric bands of 30 m each, was scouted for banana and BBTD presence and barriers to vector movement. Data were collected within both the focal plot and its buffer to evaluate the host distribution (banana density, crop composition, and vegetation diversity) and location characteristics (altitude and topography). A summary of all the variables assessed is presented in **Error! Reference source not found.**.

c. Data exploration

The dataset contained missing values that were imputed using the multiple imputation by chained equations (MICE) method from the *mice()* package for categorical variables and medians for continuous variables to enable analysis (Azur et al., 2011). To assess potential bias from imputation, we examined the standard errors of the imputed values. The dependent variable of interest was the reported number of mats with BBTD symptoms per plot, with each representing an infection event. Accordingly, count regression (Poisson and negative binomial) models were the most appropriate for assessing the statistically significant factors associated with banana BBTV infection risk. To select the

best count model, the mean and variance of the number of diseased banana plants were used to test the overdispersion of count variables. This was followed by an analysis of the number of diseased banana plants per plot.

The response variable showed a positively skewed distribution (skewness=1.9) and extremely different values for the unconditional mean (12.52) and variance (110.02), which had a nonlinear relationship, suggesting an overdispersion (**Error! Reference source not found.**). The log-likelihood ratio test for the significance of overdispersion is computed as follows:

$$Log - likelyhood\ ratio\ =\ -2\ \times\ (log - likelihood\ of\ Poisson\ regression\ - log - likelihood\ of\ negative\ binomial\ regression)$$

The value of the likelihood-ratio test showed that the data were significantly over-dispersed ($\chi2$ = 256.72, $p < 0.0001$), thus favouring the negative binomial regression model (NBRM), a generalization of the Poisson model. The NBRM permits sufficient flexibility with only two parameters (the shape parameter and mean). It subsumes the Poisson distribution, while allowing for overdispersion, where the variance may be greater than the mean.

To evaluate potential spatial autocorrelation in the dataset, we conducted a Local Indicators of Spatial Association (LISA) analysis alongside a Global Moran’s I test, using infected plant counts from all surveyed locations. A spatial weights matrix was constructed using a 10 km distance threshold to define local neighbourhoods. The Global Moran's I test produced a Moran I statistic of -0.0212 (SD: -0.588; p a ≤ 0.722), indicating no significant global spatial autocorrelation in the infected plant counts. The Local Moran's I statistics and Z-scores were computed for each location to assess clustering or dispersion at a local scale. Locations were classified based on significance thresholds, and all showed no evidence of significant spatial clustering or dispersion. Taken together, these results demonstrate

the absence of meaningful spatial autocorrelation in the dataset. We therefore employed negative binomial models, variance inflation factors (VIFs), and model selection via AIC weighting given the structure of our dataset, considering the absence of significant spatial autocorrelation.

d. Model fitting

To model the factors affecting the number of plants affected by BBTD, a negative binomial generalized linear model (NB-GLM) was used to fit the global model, which included all the variables (**Error! Reference source not found.**, Supplementary Table 1), followed by model selection and model averaging. The global model was constructed to include all hypothesized predictors, ensuring the exploration of a comprehensive model space.

e. Multicollinearity checking

To ensure the stability and interpretability of the regression model, potential linear dependencies between the predictor variables in the global model were assessed using the *alias()* function from the *stats* package (version 4.3.2) in R version 4.4.3 (R Core Team (2025). This feature was used to identify the precise linear relationships within the design matrix. It detects predictors that can be expressed as exact linear combinations of others, thereby indicating multicollinearity problems. Identifying such dependencies ensures that the model parameters are clearly identifiable and interpretable, thereby avoiding estimation problems or misleading conclusions.

Variance inflation factors (VIF) were then calculated using the *vif()* function from the package *car* (version 3.1-2) to assess multicollinearity between the predictors. This function provides generalized variance inflation factor (GVIF) values along with degrees of freedom (df) and adjusted generalized variance inflation factor $\boldsymbol{GVIF}^{\left(\frac{1}{2\times Df}\right)}$ values for each variable. Adjustment was performed to make the GVIF values comparable across the variables with different degrees of freedom. Indeed, for some variables, the associated degrees of freedom are greater than one, indicating that they are categorical

variables with multiple levels. Variables with high (>5) adjusted GVIFs were discarded to ensure that there was no serious multicollinearity that could affect the stability of regression estimates (Siril et al., 2024). **Error! Reference source not found**. summarizes the variables retained after the multicollinearity check.

f. Model selection and averaging: assessment of best combinations of descriptors.

Effective disease management in agricultural systems is typically achieved through a combination of crop management strategies, which vary depending on the knowledge, experience, and resource availability of individual farmers. Understanding how different management practices interact to influence disease dynamics is essential to develop effective and scalable interventions. We aimed to simulate a hypothetical integrated management regime by identifying combinations of explanatory variables that best accounted for the observed variation in BBTD levels. We employed an information-theoretic approach to model selection by comparing all possible subsets of predictor variables derived from a global model using the corrected Akaike Information Criterion (AICc) (Cavanaugh and Neath, 2019). This approach allows for the identification of the most parsimonious models that adequately explain the variation in the response variable while penalizing the model complexity, particularly when working with relatively small sample sizes. Model selection was implemented using the *dredge()* function of the *MuMIn* R package (version 1.47.5), which automated the process of fitting and ranking all potential subset models. This function evaluates each candidate model by computing its AICc score, thus facilitating a comprehensive and unbiased assessment of variable importance. Therefore, we systematically explored the influence of different combinations of management practices on BBTD infection, ultimately guiding the identification of the most promising strategies for integrated disease control.

The Akaike Information Criterion (AIC) is a measure of model quality relative to other models, balancing model fit and complexity. The formula for AIC is:

$$AIC = -2lo{:}L \quad + 2k$$

Where:

L = likelihood of data given for each fitted model

k = number of estimated parameters, including intercept.

For small sample sizes, AICc adjusts for potential overfitting by adding a correction term as follows:

$$AICc = AIC + \frac{2k(k+1)}{n-k-1}$$

Where:

n = sample size, and k is defined above.

This adjustment becomes significant when n is small relative to k. By penalizing models as the number of parameters approaches the sample size, AICc ensures a more conservative selection that protects against overfitting (Burnham and Anderson, 1998).

Parallel computing was utilized to enhance computational efficiency during model selection and validation. The *dredge()* function is parallelized across multiple cluster workers, allowing faster exploration of the model space. The candidate models generated by the *dredge()* function were ranked according to their AICc values, with the model exhibiting the lowest AICc considered the best-supported model. Model averaging was then performed using *avg()* function to account for model uncertainty. Models within the two AICc units of the best model were considered to have substantial support. This allowed for robust model comparison while balancing parsimony and predictive accuracy (Portet, 2020). The subset of models for averaging was selected by computing, for each model, $\Delta AICc$ which is the difference in AICc with respect to the AICc of the best candidate model.

$$\Delta AICc = AICc_i - AIC_{min}$$

Using a subset of models with $\Delta AICc$ < 2, the average coefficients and their standard errors were calculated. The $\Delta AICc$ values were also used to calculate the Akaike weight w_i of each model and the relative importance of the predictor variables appearing in the final model, using the following formula:

$$w_i = \frac{exp\left(-\frac{1}{2}\Delta AICc_i\right)}{\sum_{r=1}^{R} exp\left(-\frac{1}{2}\Delta AICc_r\right)}$$

where R is the number of models and r is the model being considered.

Note that if one of the models must be the best model, then w_i is the weight of evidence that a model is in fact the best model for the data and that all the models in the candidate subset have $w_i$that add up to 1. Finally, variable importance was assessed by calculating the relative importance values derived from the model-averaged coefficients (**Error! Reference source not found.**).

g. Evaluation of model performance

Although the AIC is widely used to identify the most parsimonious model among a set of candidates, it does not inherently measure how well a selected model fits observed data. Therefore, after selecting the best model based on AIC, we performed additional evaluations to assess its predictive adequacy. We also calculated the root-mean-square error (RMSE) and mean absolute error (MAE) as quantitative measures of prediction error. These diagnostics helped to assess whether the model captured the underlying data structure without systematic bias.

4. Results

The full model space, derived from all possible combinations of predictors in the global model, comprised 2,097,152 candidate models. Among these, Model 631,309 had the lowest AICc value (836.82), indicating that it was the most parsimonious model for the dataset. This model was used for the initial inference, although it does not account for model uncertainty because it is based on a single

best-fitting model. To address the model uncertainty, we employed model averaging. The averaged model coefficients (Table 4) are weighted across a subset of models with substantial support (ΔAICc < 2), providing more robust parameter estimates by integrating model uncertainty. This subset included 30 additional models that substantially contributed to the identification of the important predictors (Table 6). For ease of reference, these models were re-indexed from M1 to M31, with M1 representing the top-ranked model.

Model M1, which received the highest support among the subsets (accounting for 6% of the cumulative Akaike weight), identified several significant predictors of the number of BBTD-affected plants at the 5% significance level. These included plot size (m^2), type of planting material (tissue culture plantlets versus suckers), mean banana density 30-60m, presence of vegetation edges towards the east, village/location, and whether farmers used alternative seed sources not provided by the project. These variables relate globally to clean seed systems and manipulation of the abundance of vector or their behaviour (movement into gardens). Therefore, these variables should be included in future regression or modeling efforts aimed at understanding or managing BBTD.

Model M2, the second-best model, included all predictors in M1, except for banana density within a 30–60-meter radius, resulting in a model with fewer parameters. Models M2–M31 varied in the combination of included variables, often omitting some significant predictors or including others, but all had higher AICc values than M1. These differences primarily stem from the number of parameters estimated and reflected in the varying degrees of freedom. Approximately half of the models in the subset had between 9 and 11 degrees of freedom, with additional parameters contributing little explanatory power while increasing model complexity (Snipes and Taylor, 2014). This added complexity reduces the model quality by increasing the number of coefficients to be estimated without improving the fit. **Error! Reference source not found.** illustrates the relative importance of each variable across the retained models. Higher values indicate a greater likelihood of a given variable appearing in the best supported models. Overall, the best-performing models for BBTD management

aligned with the four main crop management strategies are listed in descending order of representation: (1) context-specific factors, such as garden location and plot size (28%), (2) seed system characteristics (26%), (3) banana density around the farm (16%) and vegetation barriers (10%), and (4) vector density (10%) and disease inoculum management (3%).

The best model (Table 4) included statistically significant predictors; however, several exhibited small effect sizes or wide confidence intervals, suggesting limited practical relevance. Residual plots revealed heteroscedasticity-nonconstant variance across residuals, indicating that the selected model did not fully capture all patterns in the data. This non-random structure in the residuals suggests that the model may overlook the nonlinear relationships or interactions among the variables. This also implies that the dataset may not be sufficiently robust to define the precise maximum effect sizes. Additionally, variations in the effectiveness of BBTD control measures may be influenced by context-specific environmental factors, such as altitude, temperature, and seasonal conditions, which were not fully accounted for in the model. Mean Absolute Error (MAE) and Root Mean Square Error (RMSE) are both metrics that quantify the difference between predicted values and observed values in the regression. MAE = 6.325 indicates that, on average, the predictions are off by approximately 6.33 units. The fact that the RMSE (8.757) was higher than the MAE indicates that there were some larger errors in the prediction.

5. Discussion

This study examined the field management and landscape-level factors associated with Banana Bunchy Top Disease (BBTD) infection in an endemic zone in Eastern Africa. We assessed variables related to field conditions within and surrounding smallholder banana gardens, cropping systems, vector abundance, disease prevalence, seed sourcing, and crop management practices and evaluated their association with BBTD occurrence in target plots. The project planting materials supplied to farmers

were certified plantlets from a tissue culture laboratory acclimatized in an insect-screened nursery within the village. Once planted, subsequent management decisions were independently made by farmers trained in BBTD management. This starting point–comparable production environments with differing management trajectories - allowed us to assess ecologically grounded management strategies. To the best of our knowledge, this is the first landscape-scale assessment of smallholder practices and their influence on BBTD dynamics from a known starting point. This offers insights into adaptable approaches for disease mitigation in similar agroecological settings. However, our analysis provides valuable insights into the shortcomings of some aspects of data availability. For example, the widest confidence interval ranges for the IRR were observed for the mean banana density at 30 m and 60 m. Designing an experimental protocol that captures greater variability (i.e., distance between rows and columns) could improve the practical utility and predictive power of the models for informing BBTD management strategies.

Our findings indicate that farm, buffer and landscape level diversity and landscape-scale farm crop management practices play an important role in BBTD control. Lower infection levels were associated with factors such as reduced banana density in surrounding fields, lower vector abundance, presence of vegetation barriers, seed source reliability, and more frequent field management. Notably, BBTD levels in neighboring gardens were not among the strongest predictors of infection in the target plots. This emphasizes the importance of broader community-scale management of isolated field-level interventions.

Landscape level complexity provides a relevant opportunity for the management of BBTD on a topographic scale. Banana canopy density around the recovered plot (connectivity between target plot and neighbouring plots) were associated with an increased BBTD risk in the target plot. These variables could influence the spread of vector aphids in the BBTD between mats and gardens (Kebede et al., 2018). Behavioural manipulation of insect pests and vectors through the intervention of non-host species is a powerful ecological management approach (Zhang et al., 2013). The availability of viable

BBTV susceptible hosts and the presence of backyard gardens could influence vector dispersal and virus spread dynamics. Over the past 50 years, banana-free periods and adequate buffer zones have been the most considered BBTD management options (Allen, 1978). We observed that hedges around banana gardens were associated with lower levels of infection, suggesting that vector immigration from external inoculum sources played a role in reinfection, contradicting the assumptions of Smith et al. (1998). Banana density in the buffer zone within 60 metres of the target garden was also important for predicting reinfection. Both non-host hedges and buffer zones minimize banana aphid migration into the target plot, thereby, reducing primary infection. The recommendation of large buffer zones to protect production gardens from BBTD reintroduction is not practical in smallholder systems with a high density of farmers, as it requires large land sizes for implementation (Omondi et al., 2023). A landscape-level community strategy incorporating vegetation barriers and a buffer of at least 40-60m which shifts outward as recovery advances may be more practical. Banana aphids have a restricted host spectrum and are directly affected by host distribution and BBTV (Watanabe et al., 2013, Wessels et al., 2019). Planning cropping mixtures at the landscape level would fit well with community approaches to BBTD management, especially when the choice of the most effective hedge species was identified. Therefore, selecting effective hedgerow species and incorporating vegetation barriers could enhance community-level BBTD control.

The role of infected asymptomatic banana mats remains unclear in our study, as only diseased plants were included. The distinction between infection, infectivity, and symptom expression, which is well documented in cavendish systems (Allen, 1978), was not made in this study. Incubation time, and hence the onset of symptom expression, varies across cultivars and may also be influenced by the physiological conditions of the infected plant. These factors could affect the detection and management of BBTD (Ngatat et al., 2022; Chabi et al, 2023). Asymptomatic banana mats could influence the model if such plants are significantly infectious (Cunniffe et al., 2012). The relationship between infection, infectiveness, and symptoms has been studied in BBTD, mostly in cavendish systems (Allen, 1978).

A delay between the appearance of the first symptom and infectivity was found in Cavendish and could also vary among banana cultivars (Ngatat et al., 2022). Although our study did not distinguish between infected, diseased, and infectious plants in the fields studied, Chabi et al., (2023) had found infectiousness of plants to be closely related to symptom appearance. As disease management is not uniform among the farm households studied, realistic estimates are difficult to obtain. However, all asymptomatic plants sampled were negative for BBTV when tested using ELISA. Similarly, disease progress has been shown to vary among cultivars (Ngatat et al., 2017), seasons (Niyongere et al., 2011) and can vary according to the altitude and age of crops (Leclerc et al., 2014).

Seed-sourcing practices are key determinants of BBTD risk and remain the most important route for BBTD introduction into new landscapes (Stainton et a., 2015). Farmer interest in new cultivars and cultivar diversity is an important route for the movement of seed of undeclared quality over longer distances. In this study, for example. five tissue-cultured cultivars were distributed under the project, with each farmer receiving up to three cultivars. and Additional cultivars were found in many gardens, indicating that farmers sourced extra seeds from elsewhere either to boost total plants planted or to include cultivars not multiplied by the project. Farmers who sourced planting material from informal or local sources were more likely to experience reinfection, highlighting the vulnerability of informal seed systems to seed-borne pathogens such as BBTV. Despite these risks, such informal sources remain important to farmers as they are the only route for the provision of locally preferred cultivars unavailable in formal seed systems (Simbare et al., 2020). These sourcing decisions often reflect farmers' cultivar preferences, perceived disease risks, and the use of relatively low-risk seed options as they lack mechanisms for verifying seed health (Nduwimana et al., 2022). Managing seed health, accompanied with measures to limit reinfection in the field is a key component of BBTD management (Mapinda et al., 2025). Strengthening clean seed systems in BBTD-affected areas poses logistical and social challenges, including limited availability of preferred varieties and gender-based barriers to access (McEwan et al., 2021). Practical solutions will require innovative, adaptable systems that align with farmers' demands, while ensuring seed health standards.

The removal of diseased bananas within and around the target plot was negatively associated with the infection rate in the target plot. Roguing can significantly reduce BBTV infection rates, lowering incidence to 2-10% when applied consistently and early in the disease expression stage (Omondi et al., 2020). However, the emergence of new infections in both managed and non-managed farms followed a seasonal cycle (Omondi et al., 2020), highlighting the need for time-series studies to clarify how roguing frequency, disease pressure, and environmental factors interact to shape long-term BBTD dynamics. The longer the diseased plants remain in the field, the higher is the probability of BBTV transmission to new plants in the field or landscape. Although our study did not directly assess roguing frequency owing to its cross-sectional design, its indirect effects may be inferred through plant mortality or farmer-reported roguing intensity. The relationship between roguing and infection is complex: higher reinfection rates may lead to increased roguing, whereas effective roguing may reduce infection. Time-series studies are needed to disentangle these dynamics and better quantify roguing efficacy.

Ecological interactions between the virus, vector, banana host, and other possible alternative host plants were relevant to this study. We did not assess the role of alternative hosts of BBTV or banana aphids in BBTD. To date, BBTV has not been shown to infect other Zingiberales species grown in the region, but the virus has been reported in other species, while the non-banana vector *P. caladii* has also been observed in bananas (Wessels et al., 2016; Mendoza et al., 2024) and is known to be a competent vector of BBTV (Watanabe et al., 2013). Both *P. caladii* and *P. nigronervosa* can complete their life cycles on several hosts other than banana, although *P. caladii* has a broader host range, while *P. nigronervosa* is largely restricted to *Musa* spp. (Foottit, 2010; Wessels et al., 2015). Despite both being competent BBTV vectors, *P. nigronervosa* is the more efficient transmitter. In bananas, host susceptibility and symptom expression likely contribute to disease dynamics, with evidence suggesting cultivar-level variations in BBTV accumulation and symptom severity (Niyongere et al., 2011; Ngatat et al., 2017;

2022; Chabi et al., 2023). Aphid colonization and reproduction may also vary by cultivar, influencing both vector population and transmission efficiency. Additionally, BBTV infection can alter the host preference of non-viruliferous *P. nigronervosa*, an effect lost once the aphid acquires the virus (Murhubaba et al., 2023; 2024), suggesting virus manipulation that could enhance transmission in endemic landscapes (Ingwell et al., 2012). Although this study assumed broadly similar weather conditions across geographically proximate study sites, future work should explicitly examine the influence of climatic variation as well as other factors, such as plot size, socioeconomic status, and seed exchange networks, which are known to intersect with gender and resource access (Nkengla et al., 2020; Nduwimana et al., 2022). A better understanding of these interactions could provide insight into epidemic development and improve predictive models of BBTD spread.

Our findings provide insight into the practical aspects of BBTD management. First, seed systems determine the introduction of BBTV into new gardens and landscapes and should be managed especially using indexed planting material on the one hand; and an understanding of important landraces to include in clean seed multiplication systems. Our observation on the protection of seed health agrees with modelling projections of Mapinda et al., (2025). Landscape complexity seems to function through both limiting vector dispersal and augmentative biological control through generalist predators. Generalist natural enemies may play an important role in keeping aphid populations low and preventing population build-up, which triggers dispersal. They also accelerate nymphal mortality, reducing the time spent on the host plant to acquire and inoculate the virus (Roudine et al., 2023). Studies on the specific dimensions of these correlates are important to determine their potential to improve a farmer-friendly package for BBTD management.

The findings from the model assessment against field data highlight the error risk when interpreting outputs from a single “best” model, even a model selected using AIC. Given the observed model limitations and presence of residual variance patterns (Figure 4), we advocate model averaging as a more reliable strategy. The averaged model (Table 3), which integrates the estimates from multiple

models weighted by their AIC support, provides a broader and more stable inference framework. Also, both the full and conditional model-averaged coefficients incorporate uncertainty across the model space, reducing the risk of overconfidence in any single model estimate. This approach is especially valuable in ecological and epidemiological studies where model uncertainty is substantial (Young, 2018). Moreover, the context-dependent nature of the BBTD spread suggests that some predictors may be more relevant in specific agroecological zones than others. For example, if aphid migration is the dominant transmission route in a given area, as opposed to ant-assisted or wind-mediated dispersal, control strategies should be tailored accordingly. Field experiments targeting such context-specific factors by manipulating levels of cropping mixtures content, presence of non-host barriers or selective levels of aphid and ant control should be conducted to offer more precise insights and support adaptive management strategies for BBTD.

6. Conclusion

Overall, our results highlight the interaction of landscape characteristics and field practices in shaping the BBTD dynamics. Three key factors in BBTD re-introduction into clean fields were revealed landscape complexity, farmers' seed movement, and host density, which can be consistently manipulated to achieve sustainable control. The most important variables appearing in the 31 best models reveal the importance of seed systems, control of vector movement, and a general reduction of infection pressure within and around the recovered plot in the sustainable management of BBTD. The success of these approaches may vary significantly according to latitude (or location) and weather conditions. Thus, common farmer cropping practices, such as intercropping and strip cropping, can be used to retard BBTD spread. Similarly, seed acquisition practices and direct removal of BBTD-affected plants are essential and available to farmers. We recommend that research extends these findings by using dynamic longitudinal data. Additionally, a gender-sensitive evaluation of identified technologies and practices, considering both social and environmental contexts, is essential for developing inclusive and sustainably integrated BBTD management strategies. To the best of our knowledge, this is the first

study to evaluate the spatial implications of farmer choices on BBTD within local smallholder farmer gardens. Importantly, this underscores the need to consider contextual factors in epidemiological modelling and disease control efforts in Africa.

Acknowledgments

We are grateful to the 121 farmer partners of the Banana Bunchy Top Disease Management Project in Cibitoke, Province, Burundi, whose plots yielded the datasets for their cooperation and maintenance of the pilot study sites. We also thank the leadership of the communes for assisting with mobilization at the beginning of the project. We are grateful for the support from the Ministry of Agriculture, Department of Agricultural Extension, and Burundi Institute of Agricultural Sciences (Institut des Sciences Agronomiques du Burundi, ISABU) for coordinating the BBTD recovery and seed system projects in Burundi. We also thank Abraham Shemezimana, and Emile Nitunga for support in data collection; Marius Aza and Geoffrey Dato for their support in initial data analysis and Dr. Svetlana Gaidashova, Rwanda Agriculture Board for her useful comments, which helped improve the quality of this manuscript. This study was funded by the CGIAR Cross-cutting Research Program on Root Tubers and Bananas under Cluster 3.4 Banana virus diseases.

Ethical Statement

This study involved the observation of farmers' fields for cropping, landscape, and disease assessment. It did not involve human research; therefore, this Ethical Review was not deemed applicable.

Funding Statement

Funding for this work was supported by the CIGAR Research Program on Root Tubers and Bananas (RTB) for ABO, AS, EN, CS, and CN at the Alliance of Bioversity International and CIAT and ISABU Burundi. RK, ABO, and CS were also supported by a Gates Foundation Grant on BBTD in Nigeria and Benin [INV010652], while RR was supported by The Gates Foundation Grant

[INV070408]. Under these grant conditions of the Foundation, a Creative Commons Attribution 4.0 Generic License has already been assigned to the Author Accepted Manuscript version that might arise from this submission. Further funding at the time of ABO was supported by the Plant Health Initiative of the One-CGIAR, which is grateful for the support of CGIAR Trust Fund contributors (www.cgiar.org/funders).

Data Accessibility

The datasets supporting this article have been uploaded as a part of the Supplementary Material (Table S1).

Competing Interests

We have no competing interests.'

Authors' Contributions

Conceived the study and designed study tools: ABO, CS, CN; Collected data: IN, ABO; Data analysis and interpretation: ABO, DRT, RR; Writing original draft: ABO, DRT; IN, Editing and Critically revising the draft: RR, CN, CS, DRT IN.

Approving for submission – all authors

Data availability: The original data from this study have been submitted as supplementary material (Table S1), with the descriptors in Table 1.

References

Adheka JG, Dhed’a DB, Karamura, D., Blomme, G., Swennen, R., & de Langhe, E. (2018). The morphological diversity of plantain in the Democratic Republic of Congo. *Sci. Hortic.*, **234**, 126-133. Doi: 10.17660/ActaHortic.2018.1225.36

Allen RN, 1978. Epidemiological factors influencing the success of rogueing for the control of bunchy top disease of bananas in New South Wales. *Aust. J. Agric. Res.* **29**, 535–544. Doi: 10.1071/AR9780535

Azur MJ, Stuart EA, Frangakis C, and Leaf PJ, (2011). Multiple imputation by chained equations: what is it and how does it work? *MPR*, **20**(1), 40–49. DOI: 10.1002/mpr.329

Burnham KP, and Anderson DR, (1998) Model Selection and Inference: A Practical Information-Theoretic Approach. Springer-Verlag, New York. DOI: 10.1007/978-1-4757-2917-7

Cavanaugh JE, Neath AA, (2019) The Akaike information criterion: Background, derivation, properties, application, interpretation, and refinements. *Wires Comps. Stats*.; 11: e1460. Doi: 10.1002/ wics.1460.

Chabi M, Dassou AG, Adoukonou-Sagbadja H, Thomas J, Omondi AB. (2023). Variation in symptom development and infectivity of banana bunchy top disease among four cultivars of *Musa* sp. *Crops*, **3**(2):158-169. Doi: 10.3390/crops3020016.

Cunniffe NJ, Stutt RJOH, van den Bosch F and Gilligan CA, (2012). Time-dependent infectivity and flexible latent and infectious periods in compartmental models of plant disease. *Phytopathol.* **102**, 365–380. Doi: 10.1094/PHYTO-12-10-0338.

Dato, KMG, Dégbègni MR, Atchadé MN, Hounkonnou MN, and Omondi BA, (2021). Spatial parameters associated with the risk of banana bunchy top disease in smallholder systems. *PloS ONE*, **16**(12), e0260976. DOI: 10.1371/journal.pone.0260976.

Djailo BD, Lokana J, Ngama F, Nkosi BI, Blomme G (2016). Systemicity of banana bunchy top viral infection in the Kisangani region of the Democratic Republic of Congo. *Afr. J. Agric. Res* **11**, 527-32. DOI: 10.5897/ajar2015.10641.

Fainou M, Ewédjè E-E, Kifouli A, Gustave L, Djedatin G, Affokpon A, Baba-Moussa F, and Fatiou T, (2018). Diversity of local varieties of banana and plantain cultivated in Benin. *IJBC*. 10. 497-509. DOI: 10.5897/IJBC2018.1232.

FAOSTAT (2023) FAO statistical database. https://www.fao.org/faostat/en/#data/QC. Accessed 01, June 2023

Hulme P. (2009). Trade, transport and trouble: managing invasive species pathways in an era of globalization, *J Appl. Ecol*., 46, 10 – 18. DOI: 10.1111/j.1365-2664.2008.01600.x.

Ingwell LL, Eigenbrode SD, Bosque-Perez NA (2012). Plant viruses alter insect behaviour to enhance their spread. *Sci Rep*., 2, 578. Doi: 10.1038/srep00578.

Jacobsen K, Omondi BA, Almekinders C, Alvarez E, Blomme G, Dita M, Iskra-Caruana M-L, Ocimati W, Tinzaara W, Kumar PL, and Staver C. (2019) Seed Degeneration of Banana Planting Materials: Strategies for Improved Farmer Access to Healthy Seed. *Plant Pathol.* 68, 207–228. https://doi.org/10.1111/ppa.12958.

Karamura DA, (1999). Numerical taxonomic studies of the East African highland bananas (Musa spp. AAA-East Africa) in Uganda. PhD Thesis, University of Reading, INIBAP, Montpellier, France. 192p. https://hdl.handle.net/10568/104967.

Kebede Y, Baudron F, F., Bianchi F, and Tittonell P, (2018). Unpacking the push-pull system: Assessing the contribution of companion crops along a gradient of landscape complexity. *Agric. Ecosyst. Environ.*, **268**, 115-123. doi: 10.1016/j.agee.2018.09.012.

Kumar PL, Hanna R, Alabi OJ, Vangu GH, Naidu RA (2011). Banana bunchy top virus in sub-Saharan Africa: Investigations on virus distribution and diversity. *Virus Res.* **159**, 171–82. DOI: 10.1016/j.virusres.2011.04.021.

Kumar PL, Selvarajan R, Iskra-Caruana ML, Chabannes M, Hanna R, (2015). Biology, Etiology, and Control of Virus Diseases of Banana and Plantain. *Adv Virus Res*. **91**, 229-69. DOI: 10.1016/bs.aivir.2014.10.006.

Leclerc M, Dore´ T, Gilligan CA, Lucas P, Filipe JAN (2014) Estimating the Delay between Host Infection and Disease (Incubation Period) and Assessing Its significance to the Epidemiology of Plant Diseases. *PLoS ONE,* 9(1), e86568. Doi: 10.1371/journal.pone.0086568.

Lepoint PCE, Staver C, Ajambo S, Rietveld AM, Kumar LP, Hanna R, and Caruana M-L. (2014). Workshop report: strategic planning workshop on recovering Banana production in BBTD affected areas: community and farm household approaches. https://cgspace.cgiar.org/server/api/core/bitstreams/c158a76e-0f5d-4256-abc6-6fd1a0dc39d8/content

Lepoint, P. (2016) Recovering banana production in BBTD affected areas: Strengthening cross-site learning tools in epidemiology, gender and social relations, and participatory experimentation approaches. CGIAR Research Program on Roots, Tubers and Bananas (RTB). RTB End of Project Workshop Report 2016. DOI: 10.13140/RG.2.1.1039.6323

Mapinda JJ., Hugo AK, Shinzeh JK Edward S (2025). Modelling the effects of virus-resistant planting material on the transmission dynamics of banana bunchy top disease. *Sci Rep* **15**, 20010. https://doi.org/10.1038/s41598-025-94881-7

Mbo LF, Tchinda N, Lauriane A, Cros, D, Martin G, Ndiang Z, Houegban J, Bille N, Bell JM, and Achigan-Dako, E. (2023). Analysis of genetic diversity and agronomic variation in banana sub-populations for genomic selection under drought stress in Southern Benin. *Gene*. 859. 147210. 10.1016/j.gene.2023.147210.

McEwan MA, Almekinders CMJ, Jorge JL Andrade-Piedra JJL, Delaquis E, Garrett KA, Kumar LP, Mayanja S, Omondi BA, Rajendran S, and Thiele G (2021). Breaking through the 40% adoption ceiling: Mind the seed system gaps. A perspective on seed systems research for development in One CGIAR. *Outlook Agric*. 50 (1), 5–12 doi.org/10.1177/0030727021989346

Mendoza NAP, Mendoza JS, Thomas JE, Dela Cueva FM (2024). Alternative hosts of banana bunchy top virus in the Philippines and the first evidence of seed transmission of BBTV. *Front Plant Sci*. **15**:1467331. doi: 10.3389/fpls.2024.1467331

Meyerson LA and Mooney HA (2007) Invasive alien species in an era of globalization. *Front. Ecol. and Environ*. **5**, 199-208. DOI: 10.1890/1540-9295(2007)5[199:IASIAE]2.0.CO;2

Munhoz T, Vargas J, Teixeira L, Staver C, and Dita M, (2024). Fusarium Tropical Race 4 in Latin America and the Caribbean: status and global research advances towards disease management. Frontiers in *Plant Sci.***15**, 1397617.

Murhububa SI, Tougeron K, Bragard C, Fauconnier ML, Basengere BE, Masamba JW, Hance T (2021) Banana tree infected with banana bunchy top virus attracts *Pentalonia nigronervosa* aphids through increased volatile organic compounds emission. *J Chem Ecol*., **47**:755-767. Doi: 10.1007/s10886-021-01285-0.

Murhububa IS, Bragard C, Tougeron K, Hance T (2024) Preference of *Pentalonia nigronervosa* for infected banana plants tends to reverse after *Banana bunchy top virus* acquisition. *Sci Rep* **14**, 2993 (2024). https://doi.org/10.1038/s41598-024-53205-x

Nduwimana I, Sylla S, Xing Y, Simbare A, Niyongere C, Garett K, and Omondi BA, (2022) Banana seed exchange networks in Burundi - linking formal and informal systems. *Outlook Agric*., 15 p. DOI: 10.1177/00307270221103288.

Ngatat S, Hanna R, Lienou J, Ghogomu RT, Nguidang SPK, Enoh AC, Ndemba B, Korie, S, Fotso Kuate A, Nanga Nanga, S, Fiaboe KKM, and Kumar PL (2022). *Musa* germplasm A and B genomic composition differentially affects their susceptibility to Banana Bunchy Top Virus and its aphid vector, *Pentalonia nigronervosa*. *Plants*, **11**(9), 1206. https://doi.org/10.3390/plants11091206.

Ngatat S, Hanna, R, Kumar, PL, et al., 2017. Relative susceptibility of *Musa* genotypes to banana bunchy top disease in Cameroon and implication for disease management, *Crop Prot.* **101**, 116 – 122. DOI: 10.1016/j.cropro.2017.07.018.

Niyongere C, Ateka EA, Losenge T, Lepoint P, Blomme G, (2011). Screening *Musa* genotypes for banana bunchy top disease resistance in Burundi. *Acta Hortic*., **897**, 439–47. DOI: 10.17660/ActaHortic.2011.897.60.

Niyongere C, Losenge T, Ateka EM, Ntukamazina N, Ndayiragije P, Simbare A, Cimpaye P, Nintije P, Lepoint P, Blomme G (2013) Understanding banana bunchy top disease epidemiology in Burundi for an enhanced and integrated management approach: Epidemiology of BBTD in Burundi. *Plant Pathol*, **62**, 562–570. Doi:10.1111/j.1365-3059.2012.02676.x.

Nkengla-Asi L, Omondi BA, Che Simo V, Assam E, Ngatat S, and Boonabaana B, (2020). Gender dynamics in banana seed systems and impact on banana bunchy top disease recovery in Cameroon. *Outlook Agric.* **49** (3), 235 244 doi: 10.1177/0030727020918333.

Omondi BA, Caruna M-L, and Staver C. (2021). Leveraging ecological processes and knowledge to recover banana production in BBTD-affected areas in Sub-Saharan Africa: Guidelines and new scientific challenges. In: Atta-Krah, K., Chotte, J. L., Gascuel, C., Gitz, V., Hainzelin, E., Hubert, B., ... & Sinclair, F. (eds). Agroecological transformation for sustainable food systems. Insight on France-CGIAR research. Les dossiers d'Agropolis International, 26. Agropolis International, Montpellier, France. pp. 66-67. doi: 10.23708/fdi:010082500

Omondi BA, Soko MM, Chabi M, Nduwimana I, Adjalla CC, Athindehou F, Amoussou RR, Dato GK, Tachin MZ, Niyongere C, and Staver C, (2023). Tools for the management of the banana bunchy top disease in small holder systems. *Acta Hortic*. **1367**, 233-242 DOI: 10.17660/ActaHortic.2023.1367.27

Omondi, B. A., Soko, M. M., Nduwimana, I., Delano, R. T., Niyongere, C., Simbare, A., and Staver, C. (2020). The effectiveness of consistent roguing in managing banana bunchy top disease in smallholder production in Africa. *Plant Pathol*, **69**, 1754-1766. doi: 10.1111/ ppa.13253.

Pemsl D, Staver C, Creamer B, Abdoulaye T, Alene A, and& Rusike J, (2014). Results of a global online expert survey: Major constraints, opportunities and trends for banana production and marketing and priorities for future RTB banana research. RTB Working Paper 2014-2, https://hdl.handle.net/10568/83418

Portet, S (2020) A primer on model selection using the Akaike Information Criterion, *Infect. Dis. Model.*, **5**, 111-128. Doi: 10.1016/j.idm.2019.12.010.

R Core Team (2025). *R: A Language and Environment for Statistical Computing*. R Foundation for Statistical Computing, Vienna, Austria. https://www.R-project.org.

Roudine S, Le Lann C, Bouvaine S, Le Ralec A, van Baaren J, (2023) Can biological control be a strategy to control vector-borne plant viruses? *J Pest Sci.* **96**, 451–470 https://doi.org/10.1007/s10340-022-01587-0

Simbare A, Sane CAB, Nduwimana I, Niyongere C, and Omondi BA, (2020). Diminishing Farm Diversity of East African Highland Bananas in Banana Bunchy Top Disease Outbreak Areas of Burundi—The Effect of Both Disease and Control Approaches. *Sustainability* **12**, 7467. DOI: 10.3390/su12187467.

Siril H, Gitagno D, Kaaya S, Caputo M, Hirschhorn L, Nyamuhanga T, Mtei R, Festo C, Hawkins C. (2024) Generalized and COVID related anxiety as risk factors for health outcomes among adolescents with HIV during COVID-19 in Tanzania. *Res Sq* [Preprint]. 2024 Feb 16:rs.3.rs-3921926. DOI : 10.21203/rs.3.rs-3921926/v1. PMID: 38410463; PMCID: PMC10896391.

Smith MC, Holt J, Kenyon L. and Foot C (1998) Quantitative epidemiology of Banana Bunchy Top Virus, Disease and its control, *Plant Pathol*, **47**, 177–187. DOI: 10.1046/j.1365-3059.1998.00230.x.

Snipes, M. and Taylor, D. C. (2014): Model selection and Akaike Information Criteria: An example from wine ratings and prices, *Wine Econ. Policy*, **3**, (1), 3-9. Doi: 10.1016/j.wep.2014.03.001

Stainton, D, Martin, DP, Muhire, et al. (2015). The global distribution of Banana bunchy top virus reveals little evidence for frequent recent, human-mediated long distance dispersal events. *Virus Evol*. **1**, 1-16. DOI: 10.1093/ve/vev009.

Staver, C. (2019). Ecological intensification of banana production systems. In Achieving sustainable cultivation of tropical fruits (pp. 261-322). Burleigh Dodds Science Publishing.

Swennen R, Vuylsteke D, and Ortiz R, (1995). Phenotypic diversity and patterns of variation in West and Central African plantains (*Musa* spp., AAB group Musaceae). *Econ Bot*, **49**, 320-327. Doi: 10.1007/BF02862352.

Watanabe S, Greenwell AM, Bressan A, (2013). Localization, concentration, and transmission efficiency of banana bunchy top virus in four asexual lineages of *Pentalonia* aphids. *Viruses* **5**, 758–775. Accessed on 26 June 2015. DOI: 10.3390/v5020758.

Wessels N, Omondi AB, Kruger K and Jooste E (2019) Non-destructive DNA extraction from aphids: the application in virus - vector studies of Banana bunchy top virus (BBTV) *Eur. J Plant Pathol*, **153**, 571–582. DOI: 10.1007/s10658-018-1552-2.

Young C, (2018). Model uncertainty and the crisis in science. *Socius*, 4, DOI: 2378023117737206.

Zhang Z, Sun X, Luo Z, Gao Y, and Chen Z, (2013), The manipulation mechanism of "push–pull" habitat management strategy and advances in its application, *Acta Ecol. Sin.*, **33** (2): 94-101, DOI: 10.1016/j.chnaes.2013.01.005.

Tables

Table 1. Complete list of variables and codification used before variable selection[1].

Code	Variables	Range	Levels
PSm2	Plot size in m^2	4 - 15624	null
DNIM	Distance of Nearest BBTD infected banana mat (meters)	0.275 - 197	null
BD30m	Mean of Banana density 30m (Mats/m^2)	0.002 – 0.378	null
BD60m	Mean of Banana density 60m (#Mats/m^2)	0.002 – 0.180	null
BD90m	Mean of Banana density 90m (#Mats/m^2)	0.002 – 5.501	null
RPY	rogueing per year farmer declaration	0 – 72	null
BBTD[2]	Number of plants affected by BBTD	1 - 57	null
AAP	Aphids' presence on asymptomatic mother plant	null	Yes (1), No (0)
ASP	Aphids' presence on symptomatic mother plants	null	Yes (1), No (0)
CSAM	Size of colony on Asymptomatic mats	null	Small simple colony, Large simple colonies (Mixed colonies (apterous and alate))
EPe	Edge presence East	null	Yes (1), No (0)
Eps	Edge presence South	null	Yes (1), No (0)
EPw	Edge presence West	null	Yes (1), No (0)
EPn	Edge presence North	null	Yes (1), No (0)
SeedS	Seed sources	null	1-Did not receive other **seed**s, 2-received other seeds
Var	Varieties	null	one variety (1), two varieties (2), three varieties (3), four varieties (4), more than 5 varieties (5)
OG	Plot owner gender	null	Male (1), female (2)
OPM	Other planting material on the plot	null	Yes (1), No (0)
CC	Associated crop cycle	null	Perennial (1), Seasonal (2), both (3)
MFN	Never planting material received from neighbours' or unknown sources	null	Yes (1), No (0)
MT	Type of planting material	null	Suckers (1), TC (2), Suckers + TC (3)
SF	Frequency of scouting banana infected	null	Less than monthly (1); Once a month (2); Twice a month (3); Weekly (4); Twice a week (5); Thrice a week (6); Four times a week (7); > four times a week (8)
BFP	Banana-free period	null	Yes (1), No (0)
Loc	Location	null	Munyika (1), Rusagara (2), Mugina (3), Rugombo (4)

[1] See also Supplementary Table 1
[2] Dependent variable

Table 2. List of variables included in the analysis after the collinearity test

**Variables.**	**GVIF**	**Df**	**GVIF^(1/(2*Df))**
PSm2	3.12	1	1.76
DNIM	1.47	1	1.21
BD30m	2.44	1	1.56
BD60m	2.08	1	1.44
BD90m	1.19	1	1.09
RPY	1.84	1	1.36
AAP	1.43	1	1.19
ASP	1.34	1	1.16
CSAM	3.29	2	1.35
EpE	1.91	1	1.38
EpS	2.15	1	1.47
EpW	2.63	1	1.62
EpN	1.93	1	1.39
SeedS	1.66	1	1.29
Var	7.19	4	1.28
CC	1.90	2	1.17
MFN	1.52	1	1.23
MT	3.01	2	1.32
SF	10.65	7	1.18
BFP	1.44	1	1.20
Loc	6.67	3	1.37

GVIF: Generalized variance inflation factor; GVIF^(1/(2*Df)): Adjusted generalized variance inflation factor; Df: degrees of freedom

Variable names: **AAP** - Aphids' presence on asymptomatic mother plant, **ASP** - Aphids' presence on symptomatic mother plants, **BD30m** - Mean of Banana density 30m (#Mats/m²) **BD60m** - Mean of Banana density 60m (#Mats/m²) **EpE** - Edge presence South; **EpS** - Edge presence South; **EpW**, Edge presence West; **Loc** - Location/Commune/Village; **MFN** - Never planting material received or collected from neighbours unknown ; **MT** - Type of planting material; **PSm2** (Plot size in m²) ; **RPY** - rogueing per year farmer declaration ; **SeedS** - Seed sources

Table 3. Model-Averaged Coefficients (Full and conditional averaging)

Table 4. Model-Averaged Coefficients (Full and conditional averaging)

	**Full average model** [a]				**Conditional average model** [b]			
	**Estimate**	**SE**	**IRR**	**p-value**	**Estimate**	**SE**	**IRR**	**p-value**
(Intercept)	1.832	0.306	6.246	0.000	1.830	0.306	6.234	0.000
BD30m	2.394	2.847	10.957	0.402	4.590	2.340	98.494	0.052
BD60m	3.676	3.450	39.488	0.289	5.320	2.910	204.384	0.071
EPe	0.320	0.144	1.377	0.028	0.320	0.144	1.377	0.028
Loc = Kagazi	-0.042	0.217	0.959	0.849	-0.042	0.217	0.959	0.849
Loc = Munyika	-0.623	0.210	0.536	0.003	-0.623	0.210	0.536	0.003
Loc = Rusagara	0.154	0.177	1.166	0.389	0.154	0.177	1.166	0.389
MT = TC	-1.378	1.066	0.252	0.197	-1.920	0.738	0.147	0.010
MT = Suckers + TC	0.029	0.122	1.030	0.813	0.041	0.142	1.041	0.778
PSm2	1.46e-04	3.66e-05	1.000	0.001	1.35e-4	3.86e-05	1.000	0.001
SeedS	0.364	0.141	1.439	0.010	0.364	0.141	1.439	0.010
RPY	0.001	0.003	1.001	0.680	0.006	0.004	1.006	0.146
AAP	0.166	0.233	1.181	0.478	0.351	0.223	1.420	0.120
ASP	-0.068	0.170	0.934	0.690	-0.318	0.235	0.728	0.181
MFN	-0.038	0.147	0.962	0.796	-0.362	0.295	0.696	0.225
Epw	-0.009	0.054	0.991	0.866	-0.175	0.162	0.839	0.284
Eps	-0.004	0.036	0.996	0.918	-0.151	0.173	0.860	0.389

IRR = Incidence Rate Ratios

[a] Includes all candidate models in the averaging process, regardless of whether a particular predictor is included in each model.

[b] Only includes models in which the predictor is present

AAP - Aphids' presence on asymptomatic mother plant, ASP - Aphids' presence on symptomatic mother plants, BD30m - Mean of Banana density 30m (#Mats/m^2) BD60m - Mean of Banana density 60m (#Mats/m^2) Epe - Edge presence South; Eps - Edge presence South; EPw, Edge presence West; Loc - Location/Commune/Village; MFN - Never planting material received or collected from neighbours unknown ; MT - Type of planting material; PSm2 (Plot size in m^2) ; RPY - rogueing per year farmer declaration ; SeedS - Seed sources

Table 5. Variable factors associated with changes in BBTD incidence

Predictors	Coefficients	IRR	CI	p-value
(Intercept)	1.819	6.17	4.26 – 8.99	<0.001
BD30m	4.468	87.17	0.79 – 10084.68	0.050
BD60m	4.990	146.91	0.50 – 50055.42	0.087
EPe	0.326	1.39	1.06 – 1.82	0.022
Loc = Kagazi	0.000	1.00	0.66 – 1.54	0.999
Loc = Munyika	-0.611	0.54	0.36 – 0.81	0.003
Loc = Rusagara	0.220	1.25	0.89 – 1.74	0.190
MT = TC	-2.160	0.12	0.03 – 0.49	0.002
MT = Suckers + TC	0.035	1.04	0.79 – 1.36	0.805
PSm2	1.46e-04	1.00	1.00 – 1.00	<0.001
SeedS = other seeds sources	0.319	1.38	1.06 – 1.79	0.017

IRR = Incidence Rate Ratios; CI = Confidence interval

AAP - Aphids' presence on asymptomatic mother plant, **ASP** - Aphids' presence on symptomatic mother plants, **BD30m** - Mean of Banana density 30m (#Mats/m^2) **BD60m** - Mean of Banana density 60m (#Mats/m^2) **EpE** - Edge presence South; **EpS** - Edge presence South; **EpW**, Edge presence West; **Loc** - Location/Commune/Village; **MFN** - Never planting material received or collected from neighbours unknown ; **MT** - Type of planting material; **PSm2** (Plot size in m^2) ; **RPY** - rogueing per year farmer declaration ; **SeedS** - Seed sources

Table 6. Summary of AICc results for models relating the BBTD control and the variables

**Models**		**df**	**L**	**AICc**	**ΔAICc**	w_i
**M1**	3+4+5+8+10+11+13	12	-404.97	836.82	0.00	0.06
**M2**	3+5+8+10+11+13	11	-406.43	837.29	0.46	0.05
**M3**	4+5+8+10+11+12+13	12	-405.25	837.39	0.57	0.04
**M4**	1+3+4+5+8+10+11+13	13	-404.00	837.39	0.57	0.04
**M5**	4+5+8+11+13	9	-408.96	837.53	0.71	0.04
**M6**	1+4+5+8+11+13	10	-407.77	837.54	0.72	0.04
**M7**	2+3+5+8+10+11+13	12	-405.34	837.58	0.75	0.04
**M8**	1+4+5+8+10+11+13	12	-405.45	837.79	0.97	0.04
**M9**	4+5+8+10+11+13	11	-406.69	837.80	0.98	0.04
**M10**	1+2+3+5+8+10+11+13	13	-404.20	837.80	0.98	0.04
**M11**	1+3+5+8+10+11+13	12	-405.49	837.88	1.05	0.04
**M12**	2+3+4+5+8+10+11+13	13	-404.25	837.90	1.08	0.03
**M13**	1+4+5+8+10+11+12+13	13	-404.32	838.03	1.21	0.03
**M14**	3+4+5+8+10+11+12+13	13	-404.34	838.08	1.25	0.03
**M15**	1+2+3+4+5+8+10+11+13	14	-403.11	838.18	1.35	0.03
**M16**	3+4+5+8+9+10+11+13	13	-404.40	838.21	1.39	0.03
**M17**	3+4+5+7+8+10+11+13	13	-404.41	838.22	1.40	0.03
**M18**	3+5+8+10+11+12+13	12	-405.69	838.27	1.45	0.03
**M19**	5+8+11+12+13	9	-409.33	838.28	1.46	0.03
**M20**	1+3+4+5+8+9+10+11+13	14	-403.21	838.39	1.56	0.03
**M21**	4+5+8+11+12+13	10	-408.22	838.44	1.62	0.03
**M22**	1+2+4+5+8+11+13	11	-407.04	838.50	1.68	0.03
**M23**	1+4+5+8+9+11+13	11	-407.05	838.52	1.70	0.03
**M24**	3+4+5+6+8+10+11+13	13	-404.59	838.58	1.75	0.02
**M25**	1+5+8+11+13	9	-409.50	838.62	1.80	0.02
**M26**	1+2+4+5+8+10+11+13	13	-404.63	838.65	1.83	0.02
**M27**	1+4+5+8+9+10+11+13	13	-404.64	838.68	1.86	0.02
**M28**	1+2+5+8+11+13	10	-408.35	838.70	1.87	0.02
**M29**	5+8+11+13	8	-410.73	838.75	1.93	0.02
**M30**	1+5+8+11+12+13	10	-408.38	838.76	1.93	0.02
**M31**	1+3+4+5+7+8+10+11+13	14	-403.40	838.77	1.94	0.02

$\boldsymbol{w_i}$ = Akaike weight of each model; L = Loglikelihood; AICc = Akaike Information Criterion corrected
ΔAICc = differences in AICc with respect to the AICc of the best candidate model, Df = degree of freedom
1 = Aphids' presence on asymptomatic mother plant (AAP), 2 = Aphids' presence on symptomatic mother plants (ASP), 3 = Mean of Banana density 30m (#Mats/m^2) (BD30m,) 4 = Mean of Banana density 60m (#Mats/m^2) (BD60m) 5 = Edge presence South (EPe); 6 = EPs, Edge presence South 7 = EPw, Edge presence West; 8 = Loc, Location/Commune/Village; 9 = MFN, Never planting material received or collected from neighbours unknown ; 10 = MT, Type of planting material ;11 = Plot size in m^2 (PSm2) ;12 = rogueing per year farmer declaration (RPY) 13 = Seed sources (SeedS)

Figure legends

Figure 1: Sketch of the plot assessment plan showing the target plot (green) and the areas assessed for vegetation characterization.

Figure 2: Histogram showing the density of the number of BBTD infected banana plants (for the count regression model)

Figure 3: Relative importance of selected variables based on the frequency of inclusion in the best combination of models.

Figure 4: Observed vs. Predicted values and residual diagnostics for best model fit. The symbol size corresponds to field size, filled colours represent location and symbol shape indicate seed system. These variables were chosen based on Figure 4, as they are the top three most important across all models.

Reviewer #1: Thank you for your clear and well-written work. I am interested in your manuscript and want to learn more about your research. Please see my questions below.
RESPONSE: We appreciate your kind remarks. Please find our detailed responses below.

Abstract:
The abstract should clearly state the research objectives to guide the reader and frame the study

Response: We agree to this observations

ACTION: We have added the objective linked to the approach **(Lines 3-7)**: "This study aimed to identify the key landscape, field design, and management factors influencing the persistence and re-infection of Banana Bunchy Top Disease (BBTD) in endemic smallholder systems. We assessed 121 banana gardens reestablished with virus-free tissue-culture plantlets, three years prior, to determine the combination of practices associated with low BBTD incidence."

Methods:
1. What environmental and management factors contribute to the reinfection of Banana Bunchy Top Disease (BBTD)?

ACTION: We have added details and reference on lines **123-127**: "The spread of BBTV through the vector is favoured by environmental factors that support vector aphid population build up and migration, and rapid plant growth and accumulation of BBTV titre to enable vector acquisition. These include favourable temperature, wind and topography (Niyongere et al., 2013). Thus, the greatest BBTD risk has been associated with humid lowlands."

2. Could you please elaborate on the rationale behind selecting farms exclusively replanted with BBTV-free tissue culture plantlets, considering the potential influence of pre-existing disease incidence in the study area on the observed BBTD reinfection rates?

RESPONSE: This is an important question. The BBTD existence in the vicinity provides comparable source risk around the target plots, which is important for eliminating spatial characteristics that would be associated with a new introduced area where BBTD would still be in specific hotspots. The selection of only replanted fields provides a uniform starting point and eliminates local disease history around specific topographies as a confounding factor. This way, plot history becomes more important and factors associated with active management practices can be identified.

Naturally in a BBTD infested landscape, different farms would be at different levels of epidemy development linked to the landscape and management attributes but also to the age of the plantations. By choosing the replanted gardens, we had a unique opportunity to have multiple farms that started at the same time, with comparable management systems, not much cultivar variation and whose differences would bring us closer to understanding landscape and management-linked factors influencing BBTD prevalence.

ACTION: We revised text on lines **132-139**: " As all farms shared a known replanting start date with virus-free material, they provided a consistent baseline for assessing the environmental and management factors influencing BBTD reinfection. The presence of BBTD in the surrounding landscape ensured a comparable source of inoculum across sites, minimizing spatial bias that would otherwise occur in newly affected areas where infections remain localized in hotspots. By focusing on replanted fields, we eliminated the confounding effects of local disease history and topographic variation, allowing plot-level history and active management practices to emerge as the main explanatory factors."

3. Based on the provided methodology, what specific measures were implemented to mitigate potential biases arising from subjective visual assessments of BBTD incidence during data collection?

RESPONSE: The data collection team was first trained on the implementation of the protocol in a one-week workshop outside of the data collection area. A uniform protocol with disease classification thresholds was used. BBTD symptoms follow progression from incipient levels (very easy to misdiagnose) to clearer ones at a later stage. Data taking involved cross-auditing a fraction of the gardens between data takers, evaluating the same mats previously classified.

We therefore believe the resulting bias would be random and end up in a general underestimation or overestimation. The research team had been trained in BBTD identification and had been used in the same community as trainers for two years. Our own research in Malawi, Benin, DRC and more recently in Rwanda showed that with minimal field training, farmers become quite accurate in detecting BBTD. A study done in Rwanda to assess performance and retention of BBTD identification knowledge a year after training showed practically the same result.

ACTION: Added after line **148-151:** "The data collection team underwent standardized training using a uniform disease classification protocol, implemented cross-auditing of observations between data collectors, and leveraged experienced personnel previously trained and validated in accurate BBTD identification across multiple countries."

Discussion:

1. What specific strategies were employed to ensure that the imputation process did not introduce bias or distort the true relationships within the dataset, particularly given the observed overdispersion and the eventual use of the NBRM?

Renata/Dato/

RESPONSE: Missing categorical variables were imputed using multiple imputation by chained equations (MICE). Multiple imputation generates several predictions for each missing value, allowing subsequent analyses to account for uncertainty in the imputations and produce accurate standard errors. When the observed data provide limited information about the missing values, imputations may vary widely, leading to larger standard errors, whereas highly predictive observed data yield more consistent imputations and smaller, yet still accurate, standard errors (Azur et al., 2011).

ACTION: We have added after line **178**: To assess potential bias from imputation, we examined the standard errors of the imputed values.

2. How might the observed heteroscedasticity in the residuals and the small effect sizes of some statistically significant predictors in the best model (M1) be addressed to improve the practical utility and predictive power of future BBTD management strategies?

RESPONSE**:** We thank the reviewer for this insightful question.

ACTION: We added after line **369-374**: "However, our analysis provides valuable insights into the shortcomings of some aspects of data availability. For example, the widest confidence interval ranges for the IRR were observed for the mean banana density at 30 m and 60 m. Designing an experimental protocol that captures greater variability (i.e., distance between rows and columns) could improve the practical utility and predictive power of the models for informing BBTD management strategies."

3. What effect does promptly removing diseased banana plants (roguing) have on BBTV infection rates, and what evidence suggests a need for time-series studies to tease apart the intricate link between roguing frequency, reinfection, and overall disease dynamics?

RESPONSE: Prompt roguing slows down within field infection rates and slows down disease progress. Allen 1987, had indicated the link between disease appearance and infection acquisition by aphids, resulting in disease spread. In a separate study, we showed that consistent roguing could limit disease spread and promote recovery of fields previously under BBTD infestation in field experiments in Malawi, Benin and Burundi (Omondi 2020). Roguing also supported low risk seed availability for recovery within the fields. Roguing however may not prevent primary infection arriving into the field by seed

or through immigrating aphids, hence the need for consistency and landscape scale application.

ACTION: We revised text on **lines 445-450**: "Roguing can significantly reduce BBTV infection rates, lowering incidence to 2-10% when applied consistently and early in the disease expression stage (Omondi et al., 2020). However, the emergence of new infections in both managed and non-managed farms followed a seasonal cycle (Omondi et al., 2020), highlighting the need for time-series studies to clarify how roguing frequency, disease pressure, and environmental factors interact to shape long-term BBTD dynamics.."

4. What are the ecological interactions among BBTV, its aphid vectors, the banana plants, and other possible host plants (including non-banana Zingiberales and vectors like P. caladii), and how do factors like cultivar susceptibility, aphid colonization, virus-driven changes in vector behavior, climate variation, plot size, seed/plant movement, and gender or resource access affect BBTV spread and epidemics?

RESPONSE: Studies have been carried out to asses the host diversity of banana aphids *Pentalonia nigronervosa* versus *P. caladii.* These include:

That both can complete their life cycles in various hosts other than banana and that *P. caladii* has a wider host range including banana; while *P. nigronervosa* tends to be limited to *Musa spp* (Foottit 2010; Wessels et al 2015). Both species are competent vectors of BBTV banana, but *P. nigronervosa* is more efficient. BBTV infection changes the host preference of non-viruliferous *P. nigronervosa*; but this preference is lost once the aphid acquires the virus (Muhurbaba et a., 2023; 2025). So there if virus manipulation that could accelerate the rate of spread in a landscape where the disease is present. Besides, we have shown recently that aphids growing on diseased plants multiply almost twice as fast as in healthy plants. Together these mean any presence of BBTV in the plots would strongly increase disease spread due to greater vector population output. However, for our study these factors are included in multiple dimensions including presence of aphids, density of bananas and presence of BBTD infested plants. While these are difficult to test in such a multiple scale, we note that these factors could affect observed model performance, such as significance of banana and of aphid numbers but not necessarily of BBTD infected plants.

ACTION: We added to **Lines 389- 391**: "The availability of viable BBTV susceptible hosts and the presence of backyard gardens could influence vector dispersal and virus spread dynamics. "

ACTION:

1. We change text on **lines 458** from “Ecological interactions between the virus, vector, banana host, and other possible alternative host plants were relevant to this study.” to “Ecological interactions between the virus, vector, banana host, and other possible alternative host plants are highly relevant to understanding BBTD dynamics, but they are difficult to test under natural infection conditions due to their complexity.”

2. We also added after line **463**: “Both *P. caladii* and *P. nigronervosa* can complete their life cycles on several hosts other than banana, although *P. caladii* has a broader host range, while *P. nigronervosa* is largely restricted to *Musa* spp. (Foottit, 2010; Wessels et al., 2015). Despite both being competent BBTV vectors, *P. nigronervosa* is the more efficient transmitter.

5. What are the primary drivers of *Pentalonia nigronervosa* population surges in Burundi-like landscapes? How far do viruliferous aphids disperse across typical smallholder mosaic landscapes, and which habitat features facilitate or hinder movement.

RESPONSE: To date, the actual range of aphid movement has not been measured directly. We have already added the following text the methods which partially covers the question: “The spread of BBTV through the vector is favoured by environmental factors favouring vector aphid population build up and migration, and rapid plant growth and accumulation of BBTV titre to enable vector acquisition. These include favourable temperature, wind and topography (Niyongere et al., 2013). Thus, the greatest BBTD risk BBTD has been associated with humid lowlands.” – **Line 123**

6. What are the practical implications of model averaging for interpreting BBTV risk in different agroecological zones, and how should field experiments be designed to identify context-specific drivers (e.g., aphid-driven vs. wind- or ant-mediated spread) to inform adaptive BBTD management strategies?

RESPONSE: Controlled experiments on cropping mixtures, non-host barriers and ant control have been suggested, to assess their effect on aphid distribution and disease spread. The possible role of non-host intercrops on natural enemies could also be similarly investigated; and preliminary results in our hands show that such complexity affects natural enemies in different ways. Ant studies would be interesting, especially in assessing the effects of repellents and biorational agents in BBTD control (such as soap and botanical pesticides). To date our experiments, show little effect on aphid presence, but the effects on ants would suggest a confounding relationship on aphids.

The field level management options presented here, could influence ants and aphids in diverse ways. Our focus is on their association with resultant disease appearance at this

stage. Once the list of important associations is whittled down, systematic field tests could be done to validate their efficacy and come up with the best combination of cost-effective measures for BBTD management. This effort could field experimentation, parameter estimation from field experiments followed by scenario modelling (cf Mapinda et al., 2025).

ACTION: We rephrased the last sentence of the Discussion **Line 503**: "Field experiments targeting such context-specific factors by manipulating levels of cropping mixtures content, presence of non-host barriers or selective levels of aphid and ant control should be conducted to offer more precise insights and support adaptive management strategies for BBTD."

7. Is there selection pressure on BBTV or vectors leading to more virulent strains or evasion of control measures?

RESPONSE: This is an interesting question and could require very specific studies. Indeed we know that the non-viruliferous aphids prefer infected plants; which also support faster growth of aphid populations (Adjalla et al., 2025; Muhurbuba et al, 2023; 2024). We also know that viruliferous aphids do not discriminate between infected and non-infected plants. Thus, in the presence of infected plants in a garden, there will be a net migration to spread the virus through unequal attraction and then equal dispersals.

A possible mechanism of selection for virulence could involve the delay of symptom appearance relative to the period of infectiousness of diseased plants. Allen (1987) established that three leaves would show symptoms before aphids begin migrating out and becoming an infection threat to other plants. This represents a period ranging from 3 weeks in young plantlets in the rainy season (rapid growth) to about six weeks in older plants Ngatat et al., (2017; 2022) and Chabi et al (2023) showed a variation in symptom appearance. Chabi also showed that transmission was always associated with symptom appearance such that banana aphids could not acquire BBTV from asymptomatic plants previously exposed toBBTV. As systemic virus, the duration of inoculation acquisition time and population multiplication on infected plants are important in BBTD transmission (Roudine et al 2023). So, evolution of invasion response could be strains showing symptoms much later when the virus titre can already be acquired and transmitted by aphids. This has not been studied yet but would be interesting.

ACTION: We have added after **line 470-473**:" BBTV infection can alter the host preference of non-viruliferous *P. nigronervosa*, an effect lost once the aphid acquires the virus (Muhurbaba et al., 2023; 2024), suggesting virus manipulation that could enhance transmission in endemic landscapes."
