# Supplementary material for: Drivers of banana re-infection in newly planted gardens in banana bunchy top disease endemic zones: Implications for the recovery of banana production
Source: PLoS One. 2026 Sep 11;21(9):e0357834. doi: 10.1371/journal.pone.0357834 (PMC13567820; doi:10.1371/journal.pone.0357834)
Supplement: Supplementary file 2 [file pone.0357834.s004.docx]

Reviewer #1:  Thank you for your clear and well-written work. I am interested in your manuscript and want to learn more about your research. Please see my questions below.
RESPONSE: We appreciate your kind remarks~~.~~ Please find our detailed responses below.

Abstract:
The abstract should clearly state the research objectives to guide the reader and frame the study

**Response**: We agree to this observations

ACTION: We have added the objective linked to the approach **(Lines 3-7**): “This study aimed to identify the key landscape, field design, and management factors influencing the persistence and re-infection of Banana Bunchy Top Disease (BBTD) in endemic smallholder systems. We assessed 121 banana gardens reestablished with virus-free tissue-culture plantlets, three years prior, to determine the combination of practices associated with low BBTD incidence.”

Methods:
1. What environmental and management factors contribute to the reinfection of Banana Bunchy Top Disease (BBTD)?

ACTION: We have added details and reference on lines **123-127**: “The spread of BBTV through the vector is favoured by environmental factors that support vector aphid population build up and migration, and rapid plant growth and accumulation of BBTV titre to enable vector acquisition. These include favourable temperature, wind and topography (Niyongere et al., 2013). Thus, the greatest BBTD risk has been associated with humid lowlands.”

2. Could you please elaborate on the rationale behind selecting farms exclusively replanted with BBTV-free tissue culture plantlets, considering the potential influence of pre-existing disease incidence in the study area on the observed BBTD reinfection rates?

RESPONSE: This is an important question. The BBTD existence in the vicinity provides comparable source risk around the target plots, which is important for eliminating spatial characteristics that would be associated with a new introduced area where BBTD would still be in specific hotspots. The selection of only replanted fields provides a uniform starting point and eliminates local disease history around specific topographies as a confounding factor. This way, plot history becomes more important and factors associated with active management practices can be identified.

Naturally in a BBTD infested landscape, different farms would be at different levels of epidemy development linked to the landscape and management attributes but also to the age of the plantations. By choosing the replanted gardens, we had a unique opportunity to have multiple farms that started at the same time, with comparable management systems, not much cultivar variation and whose differences would bring us closer to understanding landscape and management-linked factors influencing BBTD prevalence.

ACTION: We revised text on lines **132-139**: “ As all farms shared a known replanting start date with virus-free material, they provided a consistent baseline for assessing the environmental and management factors influencing BBTD reinfection. The presence of BBTD in the surrounding landscape ensured a comparable source of inoculum across sites, minimizing spatial bias that would otherwise occur in newly affected areas where infections remain localized in hotspots. By focusing on replanted fields, we eliminated the confounding effects of local disease history and topographic variation, allowing plot-level history and active management practices to emerge as the main explanatory factors.”

3. Based on the provided methodology, what specific measures were implemented to mitigate potential biases arising from subjective visual assessments of BBTD incidence during data collection?

RESPONSE: The data collection team was first trained on the implementation of the protocol in a one-week workshop outside of the data collection area. A uniform protocol with disease classification thresholds was used. BBTD symptoms follow progression from incipient levels (very easy to misdiagnose) to clearer ones at a later stage. Data taking involved cross-auditing a fraction of the gardens between data takers, evaluating the same mats previously classified.

We therefore believe the resulting bias would be random and end up in a general underestimation or overestimation. The research team had been trained in BBTD identification and had been used in the same community as trainers for two years. Our own research in Malawi, Benin, DRC and more recently in Rwanda showed that with minimal field training, farmers become quite accurate in detecting BBTD. A study done in Rwanda to assess performance and retention of BBTD identification knowledge a year after training showed practically the same result.

ACTION: Added after line **148-151:** “The data collection team underwent standardized training using a uniform disease classification protocol, implemented cross-auditing of observations between data collectors, and leveraged experienced personnel previously trained and validated in accurate BBTD identification across multiple countries.”

Discussion:
1. What specific strategies were employed to ensure that the imputation process did not introduce bias or distort the true relationships within the dataset, particularly given the observed overdispersion and the eventual use of the NBRM?

Renata/Dato/

RESPONSE: Missing categorical variables were imputed using multiple imputation by chained equations (MICE). Multiple imputation generates several predictions for each missing value, allowing subsequent analyses to account for uncertainty in the imputations and produce accurate standard errors. When the observed data provide limited information about the missing values, imputations may vary widely, leading to larger standard errors, whereas highly predictive observed data yield more consistent imputations and smaller, yet still accurate, standard errors (Azur et al., 2011).

ACTION: We have added after line **178**: To assess potential bias from imputation, we examined the standard errors of the imputed values.

2. How might the observed heteroscedasticity in the residuals and the small effect sizes of some statistically significant predictors in the best model (M1) be addressed to improve the practical utility and predictive power of future BBTD management strategies?

RESPONSE**:** We thank the reviewer for this insightful question.

ACTION: We added after line **369-374**: “However, our analysis provides valuable insights into the shortcomings of some aspects of data availability. For example, the widest confidence interval ranges for the IRR were observed for the mean banana density at 30 m and 60 m. Designing an experimental protocol that captures greater variability (i.e., distance between rows and columns) could improve the practical utility and predictive power of the models for informing BBTD management strategies.”

3. What effect does promptly removing diseased banana plants (roguing) have on BBTV infection rates, and what evidence suggests a need for time-series studies to tease apart the intricate link between roguing frequency, reinfection, and overall disease dynamics?

RESPONSE: Prompt roguing slows down within field infection rates and slows down disease progress. Allen 1987, had indicated the link between disease appearance and infection acquisition by aphids, resulting in disease spread. In a separate study, we showed that consistent roguing could limit disease spread and promote recovery of fields previously under BBTD infestation in field experiments in Malawi, Benin and Burundi (Omondi 2020). Roguing also supported low risk seed availability for recovery within the fields. Roguing however may not prevent primary infection arriving into the field by seed or through immigrating aphids, hence the need for consistency and landscape scale application.

ACTION: We revised text on **lines 445-450**: “Roguing can significantly reduce BBTV infection rates, lowering incidence to 2-10% when applied consistently and early in the disease expression stage (Omondi et al., 2020). However, the emergence of new infections in both managed and non-managed farms followed a seasonal cycle (Omondi et al., 2020), highlighting the need for time-series studies to clarify how roguing frequency, disease pressure, and environmental factors interact to shape long-term BBTD dynamics..”

4. What are the ecological interactions among BBTV, its aphid vectors, the banana plants, and other possible host plants (including non-banana Zingiberales and vectors like P. caladii), and how do factors like cultivar susceptibility, aphid colonization, virus-driven changes in vector behavior, climate variation, plot size, seed/plant movement, and gender or resource access affect BBTV spread and epidemics?

RESPONSE: Studies have been carried out to asses the host diversity of banana aphids *Pentalonia nigronervosa* versus *P. caladii.* These include:

That both can complete their life cycles in various hosts other than banana and that *P.* *caladii* has a wider host range including banana; while *P. nigronervosa* tends to be limited to *Musa spp* (Foottit 2010; Wessels et al 2015). Both species are competent vectors of BBTV banana, but *P. nigronervosa* is more efficient. BBTV infection changes the host preference of non-viruliferous *P. nigronervosa*; but this preference is lost once the aphid acquires the virus (Muhurbaba et a., 2023; 2025). So there if virus manipulation that could accelerate the rate of spread in a landscape where the disease is present. Besides, we have shown recently that aphids growing on diseased plants multiply almost twice as fast as in healthy plants. Together these mean any presence of BBTV in the plots would strongly increase disease spread due to greater vector population output. However, for our study these factors are included in multiple dimensions including presence of aphids, density of bananas and presence of BBTD infested plants. While these are difficult to test in such a multiple scale, we note that these factors could affect observed model performance, such as significance of banana and of aphid numbers but not necessarily of BBTD infected plants.

ACTION: We added to **Lines 389- 391**: “The availability of viable BBTV susceptible hosts and the presence of backyard gardens could influence vector dispersal and virus spread dynamics. “

ACTION:

1. We change text on **lines 458** from “Ecological interactions between the virus, vector, banana host, and other possible alternative host plants were relevant to this study.” to “Ecological interactions between the virus, vector, banana host, and other possible alternative host plants are highly relevant to understanding BBTD dynamics, but they are difficult to test under natural infection conditions due to their complexity.”

2. We also added after line **463**: “Both *P. caladii* and *P. nigronervosa* can complete their life cycles on several hosts other than banana, although *P. caladii* has a broader host range, while *P. nigronervosa* is largely restricted to *Musa* spp. (Foottit, 2010; Wessels et al., 2015). Despite both being competent BBTV vectors, *P. nigronervosa* is the more efficient transmitter.

5. What are the primary drivers of *Pentalonia nigronervosa* population surges in Burundi-like landscapes? How far do viruliferous aphids disperse across typical smallholder mosaic landscapes, and which habitat features facilitate or hinder movement.

RESPONSE: To date, the actual range of aphid movement has not been measured directly. We have already added the following text the methods which partially covers the question: “The spread of BBTV through the vector is favoured by environmental factors favouring vector aphid population build up and migration, and rapid plant growth and accumulation of BBTV titre to enable vector acquisition. These include favourable temperature, wind and topography (Niyongere et al., 2013). Thus, the greatest BBTD risk BBTD has been associated with humid lowlands.” – **Line 123**

6. What are the practical implications of model averaging for interpreting BBTV risk in different agroecological zones, and how should field experiments be designed to identify context-specific drivers (e.g., aphid-driven vs. wind- or ant-mediated spread) to inform adaptive BBTD management strategies?

RESPONSE: Controlled experiments on cropping mixtures, non-host barriers and ant control have been suggested, to assess their effect on aphid distribution and disease spread. The possible role of non-host intercrops on natural enemies could also be similarly investigated; and preliminary results in our hands show that such complexity affects natural enemies in different ways. Ant studies would be interesting, especially in assessing the effects of repellents and biorational agents in BBTD control (such as soap and botanical pesticides). To date our experiments, show little effect on aphid presence, but the effects on ants would suggest a confounding relationship on aphids.

The field level management options presented here, could influence ants and aphids in diverse ways. Our focus is on their association with resultant disease appearance at this stage. Once the list of important associations is whittled down, systematic field tests could be done to validate their efficacy and come up with the best combination of cost-effective measures for BBTD management. This effort could field experimentation, parameter estimation from field experiments followed by scenario modelling (cf Mapinda et al., 2025).

ACTION: We rephrased the last sentence of the Discussion **Line 503**: “Field experiments targeting such context-specific factors by manipulating levels of cropping mixtures content, presence of non-host barriers or selective levels of aphid and ant control should be conducted to offer more precise insights and support adaptive management strategies for BBTD.”

7. Is there selection pressure on BBTV or vectors leading to more virulent strains or evasion of control measures?

RESPONSE: This is an interesting question and could require very specific studies. Indeed we know that the non-viruliferous aphids prefer infected plants; which also support faster growth of aphid populations (Adjalla et al., 2025; Muhurbuba et al, 2023; 2024). We also know that viruliferous aphids do not discriminate between infected and non-infected plants. Thus, in the presence of infected plants in a garden, there will be a net migration to spread the virus through unequal attraction and then equal dispersals.

A possible mechanism of selection for virulence could involve the delay of symptom appearance relative to the period of infectiousness of diseased plants. Allen (1987) established that three leaves would show symptoms before aphids begin migrating out and becoming an infection threat to other plants. This represents a period ranging from 3 weeks in young plantlets in the rainy season (rapid growth) to about six weeks in older plants Ngatat et al., (2017; 2022) and Chabi et al (2023) showed a variation in symptom appearance. Chabi also showed that transmission was always associated with symptom appearance such that banana aphids could not acquire BBTV from asymptomatic plants previously exposed toBBTV. As systemic virus, the duration of inoculation acquisition time and population multiplication on infected plants are important in BBTD transmission (Roudine et al 2023). So, evolution of invasion response could be strains showing symptoms much later when the virus titre can already be acquired and transmitted by aphids. This has not been studied yet but would be interesting.

ACTION: We have added after **line 470-47****3**:” BBTV infection can alter the host preference of non-viruliferous *P. nigronervosa*, an effect lost once the aphid acquires the virus (Muhurbaba et al., 2023; 2024), suggesting virus manipulation that could enhance transmission in endemic landscapes.”
