# Supplementary material for: Drivers of banana re-infection in newly planted gardens in banana bunchy top disease endemic zones: Implications for the recovery of banana production
Source: PLoS One. 2026 Sep 11;21(9):e0357834. doi: 10.1371/journal.pone.0357834 (PMC13567820; doi:10.1371/journal.pone.0357834)
Supplement: Supplementary file 3 [file pone.0357834.s005.docx]

**View Letter**

| **Date:** | Jan 21 2026 08:04AM |
| --- | --- |
| **To:** | "Aman Bonaventure Omondi" b.a.omondi@cgiar.org |
| **From:** | "PLOS ONE" plosone@plos.org |
| **Subject:** | PLOS ONE Decision: Revision required [PONE-D-25-42829R1] |
| **Attachment(s):** | [PONE-D-25-42829R2.pdf](https://www.editorialmanager.com/pone/download.aspx?guid=%7bf3c741cb-30c6-4cf5-8f09-34c00f785ff3%7d&scheme=13&id=602107) |

PONE-D-25-42829R1

Drivers of Banana Re-infection in Newly Planted Gardens in Banana Bunch top Disease Endemic Zones: Implications for the recovery of banana production

PLOS One

Dear Dr. Omondi,

Thank you for submitting your manuscript to PLOS ONE. After careful consideration, we feel that it has merit but does not fully meet PLOS ONE’s publication criteria as it currently stands. Therefore, we invite you to submit a revised version of the manuscript that addresses the points raised during the review process.

Please submit your revised manuscript by Mar 06 2026 11:59PM. If you will need more time than this to complete your revisions, please reply to this message or contact the journal office at [plosone@plos.org](mailto:plosone@plos.org). When you're ready to submit your revision, log on to https://www.editorialmanager.com/pone/ and select the 'Submissions Needing Revision' folder to locate your manuscript file.

Please include the following items when submitting your revised manuscript:

- A letter that responds to each point raised by the academic editor and reviewer(s). You should upload this letter as a separate file labeled 'Response to Reviewers'.
- A marked-up copy of your manuscript that highlights changes made to the original version. You should upload this as a separate file labeled 'Revised Manuscript with Track Changes'.
- An unmarked version of your revised paper without tracked changes. You should upload this as a separate file labeled 'Manuscript'.

If you would like to make changes to your financial disclosure, please include your updated statement in your cover letter. Guidelines for resubmitting your figure files are available below the reviewer comments at the end of this letter.

If applicable, we recommend that you deposit your laboratory protocols in protocols.io to enhance the reproducibility of your results. Protocols.io assigns your protocol its own identifier (DOI) so that it can be cited independently in the future. For instructions see: <https://journals.plos.org/plosone/s/submission-guidelines#loc-laboratory-protocols>. Additionally, PLOS ONE offers an option for publishing peer-reviewed Lab Protocol articles, which describe protocols hosted on protocols.io. Read more information on sharing protocols at <https://plos.org/protocols?utm_medium=editorial-email&utm_source=authorletters&utm_campaign=protocols>.

We look forward to receiving your revised manuscript.

Kind regards,

Sengottayan Senthil-Nathan, Ph D
Academic Editor
PLOS One

Journal Requirements:

If the reviewer comments include a recommendation to cite specific previously published works, please review and evaluate these publications to determine whether they are relevant and should be cited. There is no requirement to cite these works unless the editor has indicated otherwise. 

Additional Editor Comments:

Reviewer(s) has serious concern with statistical analysis. Please address the issue

[Note: HTML markup is below. Please do not edit.]

Reviewers' comments:

Reviewer's Responses to Questions

**Comments to the Author**

1. If the authors have adequately addressed your comments raised in a previous round of review and you feel that this manuscript is now acceptable for publication, you may indicate that here to bypass the “Comments to the Author” section, enter your conflict of interest statement in the “Confidential to Editor” section, and submit your "Accept" recommendation.

Reviewer #1: All comments have been addressed

Reviewer #2: (No Response)

Reviewer #3: (No Response)

2. Is the manuscript technically sound, and do the data support the conclusions?

The manuscript must describe a technically sound piece of scientific research with data that supports the conclusions. Experiments must have been conducted rigorously, with appropriate controls, replication, and sample sizes. The conclusions must be drawn appropriately based on the data presented.

Reviewer #1: Yes

Reviewer #2: No

Reviewer #3: Partly

3. Has the statistical analysis been performed appropriately and rigorously?

Reviewer #1: Yes

Reviewer #2: No

Reviewer #3: Yes

4. Have the authors made all data underlying the findings in their manuscript fully available?

The [PLOS Data policy](http://www.plosone.org/static/policies.action#sharing) requires authors to make all data underlying the findings described in their manuscript fully available without restriction, with rare exception (please refer to the Data Availability Statement in the manuscript PDF file). The data should be provided as part of the manuscript or its supporting information, or deposited to a public repository. For example, in addition to summary statistics, the data points behind means, medians and variance measures should be available. If there are restrictions on publicly sharing data—e.g. participant privacy or use of data from a third party—those must be specified.

Reviewer #1: Yes

Reviewer #2: No

Reviewer #3: Yes

5. Is the manuscript presented in an intelligible fashion and written in standard English?

PLOS ONE does not copyedit accepted manuscripts, so the language in submitted articles must be clear, correct, and unambiguous. Any typographical or grammatical errors should be corrected at revision, so please note any specific errors here.

Reviewer #1: Yes

Reviewer #2: No

Reviewer #3: No

6. Review Comments to the Author

Please use the space provided to explain your answers to the questions above. You may also include additional comments for the author, including concerns about dual publication, research ethics, or publication ethics. (Please upload your review as an attachment if it exceeds 20,000 characters)

Reviewer #1: (No Response)

Reviewer #2: The current study investigated how environmental and management factors affect Banana Bunchy Top Disease (BBTD) infection in banana plants using a unique on-farm setup where virus-free tissue-cultured plantlets have been grown for three years before the investigation. The outcome of this study, if analyzed properly, is expected to provide practical and important insights into banana production. Unfortunately, however, I am not able to recommend this manuscript for publication in PLOS ONE due to several major concerns stated below.

**Response**: We thank the reviewer for this observation. We attach a revised version of our manuscript, and a response, which includes a to the concerns of the reviewers and action taken to communicate more clearly where necessary. We hope this version has significantly improved the clarity with which we communicate our results.

1. Exclusion of spatial autocorrelation analysis
A virus disease mediated by an insect vector such as BBTD is expected to cluster around the point of vector landing, making spatial autocorrelation analysis quite reasonable. The current study reported that Moran's I test detected no significant spatial autocorrelation in BBTD infection, based on which the spatial autocorrelation term was excluded from the regression equation. However, it is undeniable that the insignificant spatial autocorrelation was likely caused by the use of a 10 km threshold in the spatial weight matrix, which is logically impossible to justify in the context of the current study—especially given that the authors cite Allen (1978) for a 90m vector dispersal range in their buffer zone design. This represents a fundamental inconsistency: if vector dispersal is limited to 90m, why would spatial dependence be assessed at 10km?
Furthermore, while the process of model construction was excessively described in the Materials and Methods section (including full mathematical formulas for AIC, AICc, and Akaike weights), the type and construction method of the spatial weight matrix was not described at all. This raises serious doubts about the authors' understanding of the analytical techniques. The lack of appropriate citations to spatial econometrics and spatial analysis literature adds to this concern. –

**Response:** We thank the reviewer for raising this important point regarding spatial dependence and model specification. We agree that BBTD, as a vector-borne disease, can exhibit clustering at fine spatial scales associated with aphid-mediated dispersal. However, the objective of our spatial autocorrelation analysis was not to model plot-level vector dispersal processes, but rather to evaluate whether *between-site* dependence existed at the landscape scale relevant to our regression framework.

Our sampling units were spatially discrete fields distributed across a broader landscape, with inter-site distances typically far exceeding known aphid dispersal distances. The regression models were constructed at the site level, not at the within-field plant scale. Therefore, the relevant question for model specification was whether infection intensity at one *surveyed site* was statistically dependent on infection intensity at neighbouring *surveyed sites*.

We used a 10 km threshold in the spatial weights matrix to test for potential landscape-level spatial structure among surveyed fields. This threshold was not intended to represent vector dispersal distance; rather, it was selected to ensure that each site had a sufficient number of neighbouring sites in the weights matrix for stable estimation of Moran’s I, given the spatial configuration of the survey. Importantly, most site-to-site distances were substantially larger than aphid dispersal distances, meaning that direct epidemiological coupling between sites via short-range vector movement is biologically implausible. Thus, the spatial test was designed to detect broader landscape-level spatial dependence (e.g., shared agroecological or management contexts), not fine-scale vector clustering.

To ensure that the result was not an artefact of the 10 km threshold, we conducted sensitivity analyses using 15 km and 20 km distance bands. In all cases, Moran’s I remained near zero and non-significant. This confirms that landscape-level spatial dependence is absent and that our inference is not dependent on the chosen neighbourhood specification.

The absence of significant global or local spatial autocorrelation indicates that infection counts at surveyed sites were not spatially structured at the landscape scale considered. Under these conditions, inclusion of conditional autoregressive terms or geostatistical random fields would not be statistically justified and could introduce unnecessary model complexity and overparameterization. We have clarified this distinction in the revised manuscript and now explicitly describe the construction of the spatial weights matrix and the rationale for the chosen threshold.

We also note that our use of negative binomial regression addressed overdispersion in infection counts, and VIF diagnostics ensured limited multicollinearity among predictors. Given the absence of detectable landscape-level spatial dependence, the adopted modelling framework remains appropriate for inference at the site scale.

**Action: We have revised the Methods section to clarify these points lines ( 215-246).**

2. Unbalanced descriptions of analytical techniques
The unnecessarily redundant description of model construction implies the importance of this process in the current study. However, the Abstract contains only one sentence about this analysis: "Model selection indicated that aphid prevalence, banana density, rogueing frequency, and seed source appeared in more than half of the best-fit models..." This phrasing is problematic because "model selection" (a process) cannot logically "indicate" that variables "appeared" in models. The correct subject should be "the selected models" or "model averaging." This error, combined with the extensive but poorly integrated methodological description, suggests insufficient understanding of the analytical framework.
The disconnect between the space devoted to methodological detail in the Methods section and the minimal mention in the Abstract is difficult to understand and suggests that different sections were written without adequate integration.

**Response**: We appreciate this comment; and acknowledge a possible weakness in the summary initially provided.

**Action: the abstract has now been redone for better coherence (line 3-19)**

3. Inappropriate presentation and interpretation of parameter estimates
The Results section describes only the limitations of the model rather than the actual results of parameter estimation. The authors note "small effect sizes or wide confidence intervals," "heteroscedasticity," and "non-random structure in residuals," yet proceed to base their Discussion on this problematic model without adequately addressing whether the conclusions can be trusted given these issues.

**Response:**  Thank you for this comment. We have revised the Results section to explicitly present the parameter estimates, standard errors, 95% confidence intervals, and Incidence Rate Ratios (IRRs) for all key predictors (Tables 3–4). We have also contextualized coefficients according to the observed ranges of continuous variables, so that seemingly small coefficients (e.g., plot size) are interpreted in terms of their practical effect across the observed variable range. For categorical variables, we now clearly report the percent change in expected infection counts relative to reference categories. These changes ensure that both the magnitude and direction of effects are interpretable and provide a robust basis for discussion and management recommendations.

More concerning, the Discussion section attempts to interpret results of parameter estimation without citing any specific tables, figures, coefficient estimates, or confidence intervals. For example, statements like "Lower infection levels were associated with factors such as..." lack any quantitative support or table references. This is particularly problematic given that Tables 4 and 5 contain the coefficient estimates that should form the basis of interpretation.
This unusual structure—excessive methodological detail, minimal results reporting, and unsupported discussion—raises fundamental concerns about whether the authors can adequately defend their analytical approach and interpret their findings.

We appreciate this comment. In line with the above revision, we believe the description of the results is now clear enough.

**Action:** The discussion has been entirely rewritten to accommodate changes in the presentation and interpretation of results considering the Reviewers’ comments (lines 427-637)

**Overall recommendation**
The combination of these issues—inappropriate spatial analysis using an unjustifiable 10km threshold, inconsistent manuscript structure with poor integration between sections, fundamental misuse of statistical terminology, and inadequate interpretation of results—prevents me from recommending this manuscript for publication.

**Response**: We are grateful for the comments and views proposed, and have considered them in the new revised version of our manuscript. We wish to invite the reviewer to look again at our revision, which we believe has addressed the issues raised. We look forward to further discussion on this.

Reviewer #3: Following a large-scale tissue culture based replacement of banana plants in Burundi, the authors evaluated the status of plants 3 years later and gathered information on a number of factors related to banana fields, their surroundings, seed sourcing, and other vegetation present. Based on the reinfection of these ‘clean farms’, the authors use this information to build a negative binomial generalized linear model with BBTD incidence as the response variable.

Field data at scale on this subject is rare, so the manuscript has value for the plant pathology community. I can also see that the authors have already responded to one review and made efforts to improve the manuscript. However there are still issues with the manuscript that need to be addressed before it can be considered for publication.

Some of the methods are insufficiently detailed to be replicable. The study site section gives no description of the size of communes or distance. A map would have been useful. Without some description it is not possible for the reader to evaluate the suitability of sample selection, as well as the use of some of the geographic analysis methods (for example the use of a 10km distance threshold in lines 204-205).

We are grateful for these observations and

**Action**: We have added a map of the study area (Figure 1); and now renamed the other Figures.

The description of sampling for BBTD is also unclear. In the methods you describe ‘In each target field, two diagonal transect lines were laid along which plants were sampled at regular intervals to cover all quarters of the plot. Every third plant was assessed to determine BBTD incidence and aphid density, along two transverse transects, to cover all quarters of the garden.’ In Table 1 aphid density is not reported, only presence/absence. And in Figure 1 you show concentric square areas with subplot labels that don’t seem to match anything else in the manuscript. If you did actually lay out your plots this way on a square rather than a circle, the points where the diagonal grey lines cross into the plots are actually further than 30, 60, 90m. Either way, the description needs clarification.

We appreciate this comment. Aphids were not counted individually but rather in density classes ranging from 0 (absent) 1 (single colonies) 2 (large multiple adult colonies) to 4 large (mixed colonies with alates). Regarding the map, a perfectly uniform distance from the target plot is only possible with circular plots or those with smoothed out corners. But on the ground, many plots are usually regular (trapezoid, or qyadrilateral). We welcome any alternative presentation of the sampling unit.

**Action: The reference to aphid density classes is maintained (line 172)**

There are lots of broken citation & in-text reference links in the revised version, and a lot of spelling and punctuation errors. I am attaching a PDF with many examples highlighted, but you need to go through the whole manuscript carefully.
We have again gone through the manuscript and corrected the corrections desired.

We appreciate this comment.

Action: We have used the PDF version to correct the errors highlighted. We have also removed hyperlinks to table titles and Figure legends which were generating ‘error reference’ text in the manuscript. Besides we have carefully reread and run a spell check
In addition to the PDF, please find specific points below:

1) Please check authors' names are entered correctly in the system; this appears to include a mix of lastname-firstname and firstname-lastname.

**Action**: This has now been aligned, all authors are cited in firstname-lastname Format.

2) 48-49: ‘The banana fruit and various plant parts are consumed in multiple forms depending on the variety.’ You then go on to only give examples of the fruit – either give examples of other plant parts or leave this out.

**Response**: Agreed:

**Action**: Modified to leave out other plant parts from consumption and added the use of leaves in food preparation (Lines 52-59).

3) 80-82: ‘Small-holder production systems, dominated by local landraces, are particularly vulnerable because of their dependence on informal seed sourcing and the perennial low-input nature of the production system (Simbare et al., 2020)’ Please clarify the link between being low-input and vulnerability to BBTD.

Response: We are grateful for this observation.

**Action**: The sentence has been clarified to specifically highlight longevity of plantations, seed systems vulnerability and effect of low monitoring or replacement on BBTD (line 83-90).

4) 100-101: ‘The recovery strategy deployed in Burundi offered a case study to monitor reinfection in 121 small farms and two community nurseries for tissue culture plants in four villages.’ The way you have written this suggests you monitored reinfection in tissue culture nurseries. Please clarify.

OK the sentence has been revised. There was no reinfection in tissue culture nurseries (confirmed by symptom and ELISA testing before distribution). Monitoring was done on the fields, three years after planting.

5) 137-139: ‘By focusing on replanted fields, we eliminated the confounding effects of local disease history and topographic variation, allowing plot-level history and active management practices to emerge as the main explanatory factors’. Explain how this eliminates the effect of topography?
Action: This sentence has been reframed for better clarify (Lines: 144-149)
6) 168: So you collected gender but did not include it in the analysis? How was this determined in the majority case in which the owner is in a married couple?

**Response**: This observation is correct. The group surveyed was one that was in partnership with ISABU/Bioversity BBTD management project in Burundi. Each member (even where there were spouses) had at least one farm they were responsible for and which was included and onitored in the project to determine, for example, if the farmer met the criteria for inclusion, seed distribution and how often they got extension support or expanded their gardens. Also, a number of variables were dropped from the final analysis after the multicollinearity test (see Lines 242-272, also Table 2).

7) ‘Farm communities in the Rift Valley lost fields of the previously dominant beer banana variety, ‘Kayinja’, to Fusarium wilt race1.’ This is an empirical claim, and since it’s in the introduction we assume it is not a results of the present work.

We agree.

**Action: Citations have been added (Line 60 – 63)**

8) The manuscript does not seem sure whether altitude was included in the model or not. Please review the following conflicting statements and correct the manuscript:
o 9-10: ‘In contrast, higher altitude, the presence of hedgerows, and frequent rogueing were negatively associated with BBTD.’
o 350-352: ‘Additionally, variations in the effectiveness of BBTD control measures may be influenced by context specific environmental factors, such as altitude, temperature, and seasonal conditions, which were not fully accounted for in the model.’

**Response**: We thank the reviewer for this observation. This reference had been linked to the village with the highest altitude having the lowest mean BBTD prevalence. This has now been revised, since Altitude is not included in table 1.

**Action: revised, see line 10 – 11.**

9) Given that it is clearly one of the most important factors in your study, the seed variable is quite basic, and is treated very loosely and vaguely. In Table 1 the SeedS variable is presented as binary: either received seeds other than the project-provided, or not. Lines 433-34 directly interpret this as meaning “from informal or local sources”. Was this the case, or did the variable also include other formal sources? In Table 4 it is presented as ‘MFN - Never planting material received or collected from neighbours unknown’. Conclusions line 515 simply says ‘the importance of seed systems’. The manuscript needs extensive revision on this point to present a harmonized description throughout and provide clarity on what was actually collected here. Also consider explaining to the reader what the planting material is and some background about what’s known of its exchange in the study region.

**Action**: The discussion has been revised. It would have been great to add farmers that were entirely not in the group (did not receive planting material); but then this would have compared different periods of disease existence within the gardens. As the study was restricted to a common starting point, all farmers did receive some TC planting material. However some supplemented these with additional planting material from other sources, while others only replanted the low risk suckers from their now clean farms.

This explanation has been added to the discussion see lines 403 – 409. An introduction to types of banana seed has been added to the introduction too (lines 84 – 90)

10) Lines 321-325: among the factors associated with # of BBTD affected plants you include ‘presence of vegetation edges towards the east’ – do you have a hypothesis for why the east and no other direction? It is hard for the reader to understand your choice to include vegetation on each cardinal direction as independent variables in the model, and then when only one of them was significant you conclude that adjacent vegetation is an important management/environmental factor. Please revise how you handle this and justify its inclusion.

This was suprising to us too, but could be linked to the wind direction. Wind has been associated with aphid movement. When considered in seasonal cycles, one direction could be more important given the dominant direction of prevailing winds. We however agree that this hypothesis would need direct testing to verify (Lines 476-479).

**Action: We have edited this and added this hypothesis with citations , See lines: 429 - 435**.

11) There also seems to be a contradiction in the authors’ conclusions about the effect of neighboring plots:
o Lines 381-384: ‘Notably, BBTD levels in neighboring gardens were not among the strongest predictors of infection in the target plots. This emphasizes the importance of broader community-scale management of isolated field-level interventions.’
BBTD risk in the target plot. These variables could influence the spread of vector aphids in the BBTD between mats and gardens (Kebede et al., 2018).

o Then 387-390: ‘Banana canopy density around the recovered plot (connectivity between target plot and neighbouring plots) were associated with an increased

Response: We do not see a contradiction here, and would respectfully disagree. The banana mat density is a better descriptor of observed BBTD rates in the target plot than actual density of infected mats in the neighbouring plots. This does not seem intuitive initially, but we could hypothesise that a number of asymptomatic mats could also be contributing to aphid the strength of the infection sources. The age of infected plants may also come into play, such that a future study would compare the disease classes of the observed infections (consider Allen 1978 and Chabi et al 2023) showing the importance of disease stage in potency as an inoculum source, given the transmission mechanism of BBTV (Roudine et al., 2023). But this remains a hypothesis.

12) Lines 390-391: It is not at all clear what you mean here, or how a plant species 'intervenes'

Agreed revised: We mean barriers or mixtures of non-host species (line 481-485)

13) Lines 393-394: Not clear what ‘the most considered BBTD management options’ means, and a single 50-year old citation is not the best choice if you are trying to refer to the whole period since then, unless you are saying there has been no progress?

**Response**: We are grateful for this observation. It is indeed logical given the age of the reference used. We have now added references to show the dominance of the old methods over time.

**Action: More references added highlighting the use of roguing (with other measures) in other parts of Africa, its adoption and sustainability (lines: 619-621).**

14) Lines 394-395: ‘We observed that hedges around banana gardens were associated with lower levels of infection’ Do hedges count as ‘vegetation’ and if so their presence on the east should be associated with higher levels? What is the difference between a hedge and vegetation?

**Response**: We agree that the framing could be confusing. Vegetation is used in the sense of cultivated and random plant communities in the plots adjacent to the target plot within the measured bands of 30, 60 and 90 m; Hedge is used to define a definite strip of (non-crop) vegetation along the boundaries of the target plot. It is worth noting that boundaries between plots are typically marked using a trench, a fence of some kind (barbed wire, or woven plant debris supported by posts or a hedge).

**Action: we have edited the text , first in the methods to align with this difference, see Lines : 186 – 190) and also explained the possible logic behind a directional difference on the position of the fence (Lines 476-480)**

15) Lines 422-23: This passage mentions the use of ELISA to test plants for BBTV. This was not mentioned in the methods section.

**Response**: It is now largely understood from several studies that asymptomatic banana plants often test negative by certain methids (eg ELISA) even if infected. This has been shown in our hands in Omondi et al 2022, which was confirmed by Chabi et al 2023 – using PCR. The sentence has been reframed. ELISA testing was not done in this study. However, we note that several studies (apart from ours) have also shown limited detection of BBTD on asymptomatic plants, especially by ELISA.

**Action: the sentence has been deleted:**

16) The weakest part of the manuscript is the conclusions (lines 511-526). It does a poor job of summarizing the main results and is written in very imprecise language. The section needs comprehensive revision to make sure that each sentence is making a clear and specific point drawing from the results. Some sections simply do not make sense together. See some examples: These have been revised

**Action: The conclusion has been rewritten to make it pedagogically more logical (Lines 619-640)**

o Lines 512-514: ‘Three key factors in BBTD re-introduction into clean fields were revealed landscape complexity, farmers’ seed movement, and host density, which can be consistently manipulated to achieve sustainable control.’ This statement is too general and extrapolates beyond what you showed in this study. You have looked in a limited area, and the study only showed correlations of these factors with lower BBTD levels. There was no analysis of sustainability thresholds or attempt to apply the concepts beyond the study site. Please rewrite this section of the conclusion with more caution in expressing the conclusions.

**Response: our feeling was that whatever measures found still implemented by farmers three years since the project would represent the more sustainable measures. The conclusion has been rewritten; and a paragraph on limitations also added to the Discussion.**

o 516-519: ‘The success of these approaches may vary significantly according to latitude (or location) and weather conditions. Thus, common farmer cropping practices, such as intercropping and strip cropping, can be used to retard BBTD spread.’ The second sentence does not follow logically from the first, and the study contains no evidence that the second claim is true as it did not evaluate intercropping or strip cropping.

Action: This has been revised (lines 632-635)

o 519-520: ‘Similarly, seed acquisition practices and direct removal of BBTD-affected plants are essential and available to farmers.’ It is unclear what ‘seed acquisition practices’ means, and the sentence is grammatically incorrect.

Revised, changed to clean seed sourcing (line 635-636)

o 523-524: ‘To the best of our knowledge, this is the first study to evaluate the spatial implications of farmer choices on BBTD’. The terms ‘the spatial implications’ and ‘farmer choices on BBTD’ are too vague and imprecise to give this sentence any meaning.

We agree that this makes a claim that is not useful to the reader at this point,

**Action: We have therefore removed this statement.**

17) 535-526: ‘Importantly, this underscores the need to consider contextual factors in epidemiological modelling and disease control efforts in Africa.’ Is this really the main finding of your study? If so it is quite weak. I don’t think you will find a single pathologist that is not already doing this. Please consider ending with something more specific to your findings.

**Action**: This has also been removed

18) In the ethical statement you have written that the study did not involve humans as it was based on field observation. However, this is not strictly true, as you include survey information included (for example gender, rogueing frequency, and seed sourcing behavior).

Please revise.

**Action: This has been revised, yet further advise may be needed: Lines 657-665.**

7. PLOS authors have the option to publish the peer review history of their article ([what does this mean?](https://journals.plos.org/plosone/s/editorial-and-peer-review-process#loc-peer-review-history)). If published, this will include your full peer review and any attached files.

If you choose “no”, your identity will remain anonymous but your review may still be made public.

**Do you want your identity to be public for this peer review?** For information about this choice, including consent withdrawal, please see our [Privacy Policy](https://www.plos.org/privacy-policy).

Reviewer #1: No

Reviewer #2: **Yes:** Nobuhito Sekiya

Reviewer #3: No

[NOTE: If reviewer comments were submitted as an attachment file, they will be attached to this email and accessible via the submission site. Please log into your account, locate the manuscript record, and check for the action link "View Attachments". If this link does not appear, there are no attachment files.]

To ensure your figures meet our technical requirements, please review our figure guidelines: https://journals.plos.org/plosone/s/figures

You may also use PLOS’s free figure tool, NAAS, to help you prepare publication quality figures: https://journals.plos.org/plosone/s/figures#loc-tools-for-figure-preparation.

NAAS will assess whether your figures meet our technical requirements by comparing each figure against our figure specifications.

*In compliance with data protection regulations, you may request that we remove your personal registration details at any time.* [*(Remove my information/details)*](https://www.editorialmanager.com/pone/login.asp?a=r)*. Please contact the publication office if you have any questions.*
