# Supplementary material for: Drivers of banana re-infection in newly planted gardens in banana bunchy top disease endemic zones: Implications for the recovery of banana production
Source: PLoS One. 2026 Sep 11;21(9):e0357834. doi: 10.1371/journal.pone.0357834 (PMC13567820; doi:10.1371/journal.pone.0357834)
Supplement: Supplementary file 4 [file pone.0357834.s006.docx]

PONE-D-25-42829R2

Drivers of Banana Re-infection in Newly Planted Gardens in Banana Bunchy top Disease Endemic Zones: Implications for the recovery of banana production

PLOS One

Dear Dr. Omondi,

Thank you for submitting your manuscript to PLOS ONE. After careful consideration, we feel that it has merit but does not fully meet PLOS ONE’s publication criteria as it currently stands. Therefore, we invite you to submit a revised version of the manuscript that addresses the points raised during the review process.

Please submit your revised manuscript by Jul 25 2026 11:59PM. If you will need more time than this to complete your revisions, please reply to this message or contact the journal office at [plosone@plos.org](mailto:plosone@plos.org). When you're ready to submit your revision, log on to https://www.editorialmanager.com/pone/ and select the 'Submissions Needing Revision' folder to locate your manuscript file.

Please include the following items when submitting your revised manuscript:

- A letter that responds to each point raised by the academic editor and reviewer(s). You should upload this letter as a separate file labeled 'Response to Reviewers'.
- A marked-up copy of your manuscript that highlights changes made to the original version. You should upload this as a separate file labeled 'Revised Manuscript with Track Changes'.
- An unmarked version of your revised paper without tracked changes. You should upload this as a separate file labeled 'Manuscript'.

If you would like to make changes to your financial disclosure, please include your updated statement in your cover letter. Guidelines for resubmitting your figure files are available below the reviewer comments at the end of this letter.

If applicable, we recommend that you deposit your laboratory protocols in protocols.io to enhance the reproducibility of your results. Protocols.io assigns your protocol its own identifier (DOI) so that it can be cited independently in the future. For instructions see: <https://journals.plos.org/plosone/s/submission-guidelines#loc-laboratory-protocols>. Additionally, PLOS ONE offers an option for publishing peer-reviewed Lab Protocol articles, which describe protocols hosted on protocols.io. Read more information on sharing protocols at <https://plos.org/protocols?utm_medium=editorial-email&utm_source=authorletters&utm_campaign=protocols>.

As the corresponding author, your ORCID iD is verified in the submission system and will appear in the published article. PLOS supports the use of ORCID, and we encourage all coauthors to register for an ORCID iD and use it as well. Please encourage your coauthors to verify their ORCID iD within the submission system before final acceptance, as unverified ORCID iDs will not appear in the published article. *Only* the individual author can complete the verification step; PLOS staff *cannot* verify ORCID iDs on behalf of authors.

We look forward to receiving your revised manuscript.

Kind regards,

Sengottayan Senthil-Nathan, D.Sc
Academic Editor
PLOS One

Journal Requirements:

If the reviewer comments include a recommendation to cite specific previously published works, please review and evaluate these publications to determine whether they are relevant and should be cited. There is no requirement to cite these works unless the editor has indicated otherwise. 

Please review your reference list to ensure that it is complete and correct. If you have cited papers that have been retracted, please include the rationale for doing so in the manuscript text, or remove these references and replace them with relevant current references. Any changes to the reference list should be mentioned in the rebuttal letter that accompanies your revised manuscript. If you need to cite a retracted article, indicate the article’s retracted status in the References list and also include a citation and full reference for the retraction notice.

 Response: We appreciate and accommodate this comment in our revision

Action: References have been check and format aligned.

Additional Editor Comments:

Comments
1. Causality vs. Association: Clarify Interpretation
The abstract and discussion repeatedly use causal language (“driving”, “positively associate with”, “influencing infection”), but the study design is cross-sectional and observational. While multivariate modeling (e.g., GLM or random forest) likely underpins the reported associations, the manuscript does not explicitly state:
•Which statistical models were used (and justification for selection),
•Whether spatial autocorrelation was tested/accounted for (gardens may be clustered geographically, violating independence assumptions),
•Whether confounding (e.g., soil type, irrigation practices, proximity to infected fallow fields) was assessed or adjusted for.
Suggestion: Revise language to consistently reflect associations, not causation (e.g., “were associated with higher BBTD prevalence”). Add a brief methods subsection on model specification, diagnostics (VIF, residuals, spatial tests), and handling of potential confounders.

**Response**: The soil type was not considered in this study. While soil fertility could affect plant growth and eventual aphid density or disease trajectory, it was simply not the focus of the study.
Infected fallow fields: We believe the reviewer wishes to say fields without regular crop but which have diseased banana plants (?). All fields are considered with the presence of banana or not as a key indicator of banana density, given that the banana aphid and the BBTV do not have known alternative hosts. Aroids could have been an important crop to consider in a more controlled future study, as they have been reported to host *Pentalonia* spp.

Spatial tests are added in the Supplementary Figures following earlier recommendations. We are happy to move them back if this works.

Irrigation practices: There was no irrigation. Differences in cropping management could include intercropping, and perhaps soil fertility management. As banana is grown as a perennial, these factors are not considered in our model, we are happy to discuss them.

**Action**: The entire manuscript has been revised to remove implications of causality and put forth association as the key line of argument.

Model selection: This is an important comment and is accommodated in the manuscript. We describe the data summary and exploration to identify the most appropriate models given the data structure. Indeed we further evaluate the spatial autocorrelation as a way to test the support of our conclusions (Supplemetary Figure 1). See Lines 228 – 238.

2. The aphid quantification method lacks detail
The manuscript states “increased aphid populations” correlate with BBTD, yet it does not specify:
•How aphids were sampled (visual counts? yellow traps? leaf inspections?),
•Timing and frequency (single snapshot vs. seasonal monitoring),
•Whether *Pentalonia nigronervosa* (the sole BBTV vector) was confirmed morphologically or molecularly, or whether non-vector aphids were inadvertently included.

Suggestion: Clarify sampling protocol in Methods (ideally with a citation or standard protocol reference, e.g., IITA or FAO guidelines). If *P. nigronervosa* identification wasn’t verified, explicitly acknowledge this as a limitation.

Identification wasn’t verified; explicitly acknowledge this as a limitation.

**Response**: We agree with the reviewer on the need to add some possible limitation, which has been duly added. We however point out that due to vector host specificity, most colony-forming aphids on banana will be banana aphids (*Pentalonia nigronervosa*, or rarely *P. caladii*) (See Foottit 2010). Indeed aphids collected by us in multiple countries (Uganda, DRC, Burundi – same gardens as this study; Rwanda, Benin and Malawi) targeting multiple Musaceae hosts showed than a very small sample of banana aphids only from DRC were *P caladii,* the rest being *P.* *nigronervosa*. No other aphid species was recovered forming colonies on banana (See Wessels et al., 2019).

**Action**: We add a paragraph on aphid sampling (lines 186-200) with references. The limitation of potential misidentification of aphids is addressed in the discussion (lines 650 – 675) alongside other potential limitations

3. Rogueing definition and fidelity are ambiguous
“Frequent rogueing” is cited as protective, but no definition is provided:
•Was it self-reported by farmers? Observed? Documented in logs?
•What constituted “rogueing” — removal of symptomatic plants only? Or also asymptomatic neighbors (a recommended practice)?
•Was timing (e.g., pre- or post-symptom onset) recorded?

Suggestion: Define rogueing operationally in Methods and discuss the reliability of measurement (e.g., inter-farmer variability, recall bias). Consider adding a brief validation note if field staff verified reports.

**Response**: Roguing of asymptomatic neighbours is not really presently recommended, as BBTD does not spread in a diffusion model from a central plant. We have used the approach we previously tested (see Omondi et al., 2020) in Malawi, Burundi (this community) and Benin, involving regular roguing of diseases plants only. Some asymptomatic plants could be missed, at the tune of 2% (see Omondi et al., 2020). Where monthly roguing is done, asymptomatic plants were not significant subsequent infection sources (Chabi et al., 2023) and would be removed in subsequent assessment. Thus, only symptomatic plants were removed.

Asymptomatic mats close to the diseased plant were not removed (See Omondi et al., 2020; and 2026 – which tested this approach). This is not the recommended practice where monitoring is continuous, and is particularly repulsive to smallholder farmers due to the potential heavy loss of plants. Out later studies (Chabi et al., 2023) did show, in three cultivars, that aphids were not as successful in transmitting the virus from asymptomatic exposed plants to healthy ones. Spatial assessment in Australia (John Thomas, personal communication) since 2019 are consistent with this assessment. So only backyard bananas are treated like this given that they are not subjected to frequent/ consistent monitoring. We agree with the reviewer that farmer reported roguing without a parallel monthly technical log could be erroneous, but this kind of study was already done by us (Omondi et al., 2020) when we sought to evaluate the performance of roguing among smallholders. In this study, monthly reporting and three layer supervision (Farmer Group, Extension officer and Project Technician) was included. The bias of farmer reported roguing is addressed in the discussion and could explain why roguing does not come up strongly as associated with BBTD prevalence.

**Action**: We have now added a definition of the roguing protocol used and the reference in lines 206 – 2011 (corrected version with Track changes). That farmer-reported roguing was done is and added limitations associated with it in the text (lines the limitations of the approach is explained in the text) lines 211 – 214.

4. Lack of BBTV detection confirmation
The study assumes BBTD diagnosis = BBTV infection. However, visual symptoms can mimic other stresses (e.g., nematode damage, drought, nutrient deficiency). No mention is made of:
• Diagnostic confirmation (e.g., PCR, ELISA, or lateral flow assays) on a subsample,
• Inter-rater reliability of symptom scoring (if multiple enumerators were used).

Suggestion: State whether lab confirmation was performed — and if not, add a clear limitation acknowledging possible misclassification bias, which may attenuate or distort observed associations.

**Response**: This limitation has now been addressed. In an associated study, we had carried out ELISA and PCR tests (Burundi and Benin respectively) to verify infection. All symptomatic plants were positive in these studies.

While we agree that some symptoms of BBTD could resemble nutrient deficiency symptoms, especially Potassium; BBTD presents multiple symptoms that together are used to diagnose the disease symptomatically quite accurately. We have added this evaluation to the text,

**Action**: We have now added a definition of the roguing protocol used and the reference in lines 206 – 2011 (corrected version with Track changes). That farmer-reported roguing was done is and added limitations associated with it in the text (lines the limitations of the approach is explained in the text) lines 211 – 214.

**Reviewer #1: Strengths**
•Timely and applied relevance: BBTD remains a critical constraint to food security and livelihoods in smallholder banana systems across Africa and Asia. This study addresses a practical, underexplored question: why do virus-free plantings still fail?
•Strong field design: The assessment of 121 independently managed gardens — all established with certified virus-free plantlets — provides valuable real-world context beyond controlled trials.
•Ecologically grounded variables: Inclusion of landscape-level factors (elevation, hedgerows, varietal diversity, rogueing frequency) reflects a systems-thinking approach aligned with agroecological disease management principles.
•Clear translational messaging: Recommendations (e.g., barrier plants, mixed cropping, community rogueing protocols) are actionable and farmer-informed — highly appropriate for PLOS ONE’s mission of accessible, policy-relevant science.

Comments
1. Causality vs. Association: Clarify Interpretation
The abstract and discussion repeatedly use causal language (“driving”, “positively associate with”, “influencing infection”), but the study design is cross-sectional and observational. While multivariate modeling (e.g., GLM or random forest) likely underpins the reported associations, the manuscript does not explicitly state:
•Which statistical models were used (and justification for selection),
•Whether spatial autocorrelation was tested/accounted for (gardens may be clustered geographically, violating independence assumptions),
•Whether confounding (e.g., soil type, irrigation practices, proximity to infected fallow fields) was assessed or adjusted for.
Suggestion: Revise language to consistently reflect associations, not causation (e.g., “were associated with higher BBTD prevalence”). Add a brief methods subsection on model specification, diagnostics (VIF, residuals, spatial tests), and handling of potential confounders.

Response:

**Response**: The soil type was not considered in this study. While soil fertility could affect plant growth and eventual aphid density or disease trajectory, it was simply not the focus of the study.
Infected fallow fields: We believe the reviewer wishes to say fields without regular crop but which have diseased banana plants (?). All fields are considered with the presence of banana or not as a key indicator of banana density, given that the banana aphid and the BBTV do not have known alternative hosts. Aroids could have been an important crop to consider in a more controlled future study, as they have been reported to host *Pentalonia* spp.

Spatial tests are added in the Supplementary Figures following earlier recommendations. We are happy to move them back if this works.

Irrigation practices: There was no irrigation. Differences in cropping management could include intercropping, and perhaps soil fertility management. As banana is grown as a perennial, these factors are not considered in our model, we are happy to discuss them.

**Action**: The entire manuscript has been revised to remove implications of causality and put forth association as the key line of argument.

Model selection: This is an important comment and is accommodated in the manuscript. We describe the data summary and exploration to identify the most appropriate models given the data structure. Indeed we further evaluate the spatial autocorrelation as a way to test the support of our conclusions (Supplemetary Figure 1). See Lines 228 – 238.

2. The aphid quantification method lacks detail
The manuscript states “increased aphid populations” correlate with BBTD, yet it does not specify:
•How aphids were sampled (visual counts? yellow traps? leaf inspections?),
•Timing and frequency (single snapshot vs. seasonal monitoring),
•Whether *Pentalonia nigronervosa* (the sole BBTV vector) was confirmed morphologically or molecularly, or whether non-vector aphids were inadvertently included.

**Suggestion**: Clarify sampling protocol in Methods (ideally with a citation or standard protocol reference, e.g., IITA or FAO guidelines). If *P. nigronervosa* identification wasn’t verified, explicitly acknowledge this as a limitation.

Identification wasn’t verified; explicitly acknowledge this as a limitation.

**Response**: We agree with the reviewer on the need to add some possible limitation, which has been duly added. We however point out that due to vector host specificity, most colony-forming aphids on banana will be banana aphids (*Pentalonia nigronervosa*, or rarely *P. caladii*) (See Foottit 2010). Indeed aphids collected by us in multiple countries (Uganda, DRC, Burundi – same gardens as this study; Rwanda, Benin and Malawi) targeting multiple Musaceae hosts showed than a very small sample of banana aphids only from DRC were *P caladii,* the rest being *P.* *nigronervosa*. No other aphid species was recovered forming colonies on banana (See Wessels et al., 2019).

**Action**: We add a paragraph on aphid sampling (lines 186-200) with references. The limitation of potential misidentification of aphids is addressed in the discussion (lines 650 – 675) alongside other potential limitations

3. Rogueing definition and fidelity are ambiguous
“Frequent rogueing” is cited as protective, but no definition is provided:
•Was it self-reported by farmers? Observed? Documented in logs?
•What constituted “rogueing” — removal of symptomatic plants only? Or also asymptomatic neighbors (a recommended practice)?
•Was timing (e.g., pre- or post-symptom onset) recorded?

**Suggestion**: Define rogueing operationally in Methods and discuss the reliability of measurement (e.g., inter-farmer variability, recall bias). Consider adding a brief validation note if field staff verified reports.

**Response**: We appreciate this comment and accommodate it in the revision. Asymptomatic mats close to the diseased plant were not removed (See Omondi et al., 2020; and 2026 – which tested this approach). This is not the recommended practice where monitoring is continuous, and is particularly repulsive to smallholder farmers due to the potential heavy loss of plants. Out later studies (Chabi et al., 2023) did show, in three cultivars, that aphids were not as successful in transmitting the virus from asymptomatic exposed plants to healthy ones. Spatial assessment in Australia (John Thomas, personal communication) since 2019 are consistent with this assessment. So only backyard bananas are treated like this given that they are not subjected to frequent/ consistent monitoring. We agree with the reviewer that farmer reported roguing without a parallel monthly technical log could be erroneous, but this kind of study was already done by us (Omondi et al., 2020) when we sought to evaluate the performance of roguing among smallholders. In this study, monthly reporting and three layer supervision (Farmer Group, Extension officer and Project Technician) was included.

**Action**: We have now added a definition of the roguing protocol used and the reference in lines 206 – 2011 (corrected version with Track changes). That farmer-reported roguing was done is and added limitations associated with it in the text (lines the limitations of the approach is explained in the text) lines 211 – 214.

4. Lack of BBTV detection confirmation
The study assumes BBTD diagnosis = BBTV infection. However, visual symptoms can mimic other stresses (e.g., nematode damage, drought, nutrient deficiency). No mention is made of:
• Diagnostic confirmation (e.g., PCR, ELISA, or lateral flow assays) on a subsample,
• Inter-rater reliability of symptom scoring (if multiple enumerators were used).

Suggestion: State whether lab confirmation was performed — and if not, add a clear limitation acknowledging possible misclassification bias, which may attenuate or distort observed associations.

**Response: Laboratory confirmation of infection was not done in this study.**

This limitation has now been addressed. In an associated study, we had carried out ELISA and PCR tests (Burundi and Benin respectively) to verify infection. All symptomatic plants were positive in these studies. Further, about 1-2% of asymptomatic plants tested positive for BBTV (See Omondi et al., 2020).

While we agree that some symptoms of BBTD could resemble nutrient deficiency symptoms, especially Potassium; BBTD presents multiple symptoms that together are used to diagnose the disease symptomatically quite accurately. We have added this evaluation to the text and cite previously published papers that clearly describe the symptom progression we used (eg Chabi et al., 2023).

**Action:** We have added a discussion paragraph on technical limitations in the text (Lines 657-675).
