# Supplementary material for: Drivers of banana re-infection in newly planted gardens in banana bunchy top disease endemic zones: Implications for the recovery of banana production
Source: PLoS One. 2026 Sep 11;21(9):e0357834. doi: 10.1371/journal.pone.0357834 (PMC13567820; doi:10.1371/journal.pone.0357834)
Supplement: Peer review history

# Peer review: Decision Letter 0

**DOI:** 10.1371/journal.pone.0357834.r001

**Authors:** Sarah Jose

7 Oct 2025

Dear Dr. Omondi,

Thank you for submitting your manuscript to PLOS ONE. After careful consideration, we feel that it has merit but does not fully meet PLOS ONE’s publication criteria as it currently stands. Therefore, we invite you to submit a revised version of the manuscript that addresses the points raised during the review process.

Please submit your revised manuscript by Nov 20 2025 11:59PM. If you will need more time than this to complete your revisions, please reply to this message or contact the journal office at plosone@plos.org. When you're ready to submit your revision, log on to https://www.editorialmanager.com/pone/ and select the 'Submissions Needing Revision' folder to locate your manuscript file.

- A rebuttal letter that responds to each point raised by the academic editor and reviewer(s). You should upload this letter as a separate file labeled 'Response to Reviewers'.
- A marked-up copy of your manuscript that highlights changes made to the original version. You should upload this as a separate file labeled 'Revised Manuscript with Track Changes'.
- An unmarked version of your revised paper without tracked changes. You should upload this as a separate file labeled 'Manuscript'.

If you would like to make changes to your financial disclosure, please include your updated statement in your cover letter. Guidelines for resubmitting your figure files are available below the reviewer comments at the end of this letter.

If applicable, we recommend that you deposit your laboratory protocols in protocols.io to enhance the reproducibility of your results. Protocols.io assigns your protocol its own identifier (DOI) so that it can be cited independently in the future. For instructions see: https://journals.plos.org/plosone/s/submission-guidelines#loc-laboratory-protocols. Additionally, PLOS ONE offers an option for publishing peer-reviewed Lab Protocol articles, which describe protocols hosted on protocols.io. Read more information on sharing protocols at https://plos.org/protocols?utm_medium=editorial-email&utm_source=authorletters&utm_campaign=protocols.

We look forward to receiving your revised manuscript.

Kind regards,

Sarah Jose, Ph.D.

Staff Editor

PLOS ONE

Journal requirements:

When submitting your revision, we need you to address these additional requirements.

1. Please ensure that your manuscript meets PLOS ONE's style requirements, including those for file naming. The PLOS ONE style templates can be found at

https://journals.plos.org/plosone/s/file?id=wjVg/PLOSOne_formatting_sample_main_body.pdf and

https://journals.plos.org/plosone/s/file?id=ba62/PLOSOne_formatting_sample_title_authors_affiliations.pdf

2. In your Methods section, please provide additional information regarding the permits you obtained for the work. Please ensure you have included the full name of the authority that approved the field site access and, if no permits were required, a brief statement explaining why.

3. Please note that PLOS One has specific guidelines on code sharing for submissions in which author-generated code underpins the findings in the manuscript. In these cases, we expect all author-generated code to be made available without restrictions upon publication of the work. Please review our guidelines at https://journals.plos.org/plosone/s/materials-and-software-sharing#loc-sharing-code and ensure that your code is shared in a way that follows best practice and facilitates reproducibility and reuse.

4. Thank you for stating the following financial disclosure:

[Funding for this work was supported by the CIGAR Research Program on Root Tubers and Bananas (RTB) for ABO, AS, EN, CS, and CN at the Alliance of Bioversity International and CIAT and ISABU Burundi. RK, ABO, and CS were also supported by a Gates Foundation Grant on BBTD in Nigeria and Benin [INV010652], while RR was supported by The Gates Foundation Grant [INV070408]. Under these grant conditions of the Foundation, a Creative Commons Attribution 4.0 Generic License has already been assigned to the Author Accepted Manuscript version that might arise from this submission. Further funding at the time of ABO was supported by the Plant Health Initiative of the One-CGIAR, which is grateful for the support of CGIAR Trust Fund contributors (www.cgiar.org/funders).].

Please state what role the funders took in the study.  If the funders had no role, please state: "The funders had no role in study design, data collection and analysis, decision to publish, or preparation of the manuscript."

If this statement is not correct you must amend it as needed.

Please include this amended Role of Funder statement in your cover letter; we will change the online submission form on your behalf.

5. Your ethics statement should only appear in the Methods section of your manuscript. If your ethics statement is written in any section besides the Methods, please move it to the Methods section and delete it from any other section. Please ensure that your ethics statement is included in your manuscript, as the ethics statement entered into the online submission form will not be published alongside your manuscript.

6. We note that Figure 1 in your submission contain [map/satellite] images which may be copyrighted. All PLOS content is published under the Creative Commons Attribution License (CC BY 4.0), which means that the manuscript, images, and Supporting Information files will be freely available online, and any third party is permitted to access, download, copy, distribute, and use these materials in any way, even commercially, with proper attribution. For these reasons, we cannot publish previously copyrighted maps or satellite images created using proprietary data, such as Google software (Google Maps, Street View, and Earth). For more information, see our copyright guidelines: http://journals.plos.org/plosone/s/licenses-and-copyright.

We require you to either (1) present written permission from the copyright holder to publish these figures specifically under the CC BY 4.0 license, or (2) remove the figures from your submission:

1. You may seek permission from the original copyright holder of Figure 1 to publish the content specifically under the CC BY 4.0 license.

We recommend that you contact the original copyright holder with the Content Permission Form (http://journals.plos.org/plosone/s/file?id=7c09/content-permission-form.pdf) and the following text:

“I request permission for the open-access journal PLOS ONE to publish XXX under the Creative Commons Attribution License (CCAL) CC BY 4.0 (http://creativecommons.org/licenses/by/4.0/). Please be aware that this license allows unrestricted use and distribution, even commercially, by third parties. Please reply and provide explicit written permission to publish XXX under a CC BY license and complete the attached form.”

Please upload the completed Content Permission Form or other proof of granted permissions as an "Other" file with your submission.

In the figure caption of the copyrighted figure, please include the following text: “Reprinted from [ref] under a CC BY license, with permission from [name of publisher], original copyright [original copyright year].”

2. If you are unable to obtain permission from the original copyright holder to publish these figures under the CC BY 4.0 license or if the copyright holder’s requirements are incompatible with the CC BY 4.0 license, please either i) remove the figure or ii) supply a replacement figure that complies with the CC BY 4.0 license. Please check copyright information on all replacement figures and update the figure caption with source information. If applicable, please specify in the figure caption text when a figure is similar but not identical to the original image and is therefore for illustrative purposes only.

The following resources for replacing copyrighted map figures may be helpful:

USGS National Map Viewer (public domain): http://viewer.nationalmap.gov/viewer/

The Gateway to Astronaut Photography of Earth (public domain): http://eol.jsc.nasa.gov/sseop/clickmap/

Maps at the CIA (public domain): https://www.cia.gov/library/publications/the-world-factbook/index.html and https://www.cia.gov/library/publications/cia-maps-publications/index.html

NASA Earth Observatory (public domain): http://earthobservatory.nasa.gov/

Landsat: http://landsat.visibleearth.nasa.gov/

USGS EROS (Earth Resources Observatory and Science (EROS) Center) (public domain): http://eros.usgs.gov/#

Natural Earth (public domain): http://www.naturalearthdata.com/

7. If the reviewer comments include a recommendation to cite specific previously published works, please review and evaluate these publications to determine whether they are relevant and should be cited. There is no requirement to cite these works unless the editor has indicated otherwise.

Additional Editor Comments (if provided):

[Note: HTML markup is below. Please do not edit.]

Reviewers' comments:

Reviewer's Responses to Questions

**Comments to the Author**

1. Is the manuscript technically sound, and do the data support the conclusions?

Reviewer #1: Yes

**********

2. Has the statistical analysis been performed appropriately and rigorously?

Reviewer #1: Yes

**********

3. Have the authors made all data underlying the findings in their manuscript fully available?

The PLOS Data policy

Reviewer #1: Yes

**********

4. Is the manuscript presented in an intelligible fashion and written in standard English?

Reviewer #1: Yes

**********

Reviewer #1: Thank you for your clear and well-written work. I am interested in your manuscript and want to learn more about your research. Please see my questions below.

Abstract:

The abstract should clearly state the research objectives to guide the reader and frame the study

Methods:

1. What environmental and management factors contribute to the reinfection of Banana Bunchy Top Disease (BBTD)?

2. Could you please elaborate on the rationale behind selecting farms exclusively replanted with BBTV-free tissue culture plantlets, considering the potential influence of pre-existing disease incidence in the study area on the observed BBTD reinfection rates?

3. Based on the provided methodology, what specific measures were implemented to mitigate potential biases arising from subjective visual assessments of BBTD incidence during data collection?

Discussion:

1. What specific strategies were employed to ensure that the imputation process did not introduce bias or distort the true relationships within the dataset, particularly given the observed overdispersion and the eventual use of the NBRM?

2. How might the observed heteroscedasticity in the residuals and the small effect sizes of some statistically significant predictors in the best model (M1) be addressed to improve the practical utility and predictive power of future BBTD management strategies?

3. What effect does promptly removing diseased banana plants (roguing) have on BBTV infection rates, and what evidence suggests a need for time-series studies to tease apart the intricate link between roguing frequency, reinfection, and overall disease dynamics?

4. What are the ecological interactions among BBTV, its aphid vectors, the banana plants, and other possible host plants (including non-banana Zingiberales and vectors like P. caladii), and how do factors like cultivar susceptibility, aphid colonization, virus-driven changes in vector behavior, climate variation, plot size, seed/plant movement, and gender or resource access affect BBTV spread and epidemics?

5. What are the primary drivers of Pentalonia nigronervosa population surges in Burundi-like landscapes? How far do viruliferous aphids disperse across typical smallholder mosaic landscapes, and which habitat features facilitate or hinder movement

6. What are the practical implications of model averaging for interpreting BBTV risk in different agroecological zones, and how should field experiments be designed to identify context-specific drivers (e.g., aphid-driven vs. wind- or ant-mediated spread) to inform adaptive BBTD management strategies? Is there selection pressure on BBTV or vectors leading to more virulent strains or evasion of control measures?

**********

what does this mean?). If published, this will include your full peer review and any attached files.

If you choose “no”, your identity will remain anonymous but your review may still be made public.

**Do you want your identity to be public for this peer review?**  For information about this choice, including consent withdrawal, please see our Privacy Policy

Reviewer #1: No

**********

[NOTE: If reviewer comments were submitted as an attachment file, they will be attached to this email and accessible via the submission site. Please log into your account, locate the manuscript record, and check for the action link "View Attachments". If this link does not appear, there are no attachment files.]

While revising your submission, please upload your figure files to the Preflight Analysis and Conversion Engine (PACE) digital diagnostic tool, https://pacev2.apexcovantage.com/. PACE helps ensure that figures meet PLOS requirements. To use PACE, you must first register as a user. Registration is free. Then, login and navigate to the UPLOAD tab, where you will find detailed instructions on how to use the tool. If you encounter any issues or have any questions when using PACE, please email PLOS at figures@plos.org. Please note that Supporting Information files do not need this step.

# Author comment: Author response to Decision Letter 1

**DOI:** 10.1371/journal.pone.0357834.r002

23 Oct 2025

Reviewer #1: Thank you for your clear and well-written work. I am interested in your manuscript and want to learn more about your research. Please see my questions below.

RESPONSE: We appreciate your kind remarks. Please find our detailed responses below.

Abstract:

The abstract should clearly state the research objectives to guide the reader and frame the study

Response: We agree to this observations

ACTION: We have added the objective linked to the approach (Lines 3-7): “This study aimed to identify the key landscape, field design, and management factors influencing the persistence and re-infection of Banana Bunchy Top Disease (BBTD) in endemic smallholder systems. We assessed 121 banana gardens reestablished with virus-free tissue-culture plantlets, three years prior, to determine the combination of practices associated with low BBTD incidence.”

Methods:

1. What environmental and management factors contribute to the reinfection of Banana Bunchy Top Disease (BBTD)?

ACTION: We have added details and reference on lines 123-127: “The spread of BBTV through the vector is favoured by environmental factors that support vector aphid population build up and migration, and rapid plant growth and accumulation of BBTV titre to enable vector acquisition. These include favourable temperature, wind and topography (Niyongere et al., 2013). Thus, the greatest BBTD risk has been associated with humid lowlands.”

2. Could you please elaborate on the rationale behind selecting farms exclusively replanted with BBTV-free tissue culture plantlets, considering the potential influence of pre-existing disease incidence in the study area on the observed BBTD reinfection rates?

RESPONSE: This is an important question. The BBTD existence in the vicinity provides comparable source risk around the target plots, which is important for eliminating spatial characteristics that would be associated with a new introduced area where BBTD would still be in specific hotspots. The selection of only replanted fields provides a uniform starting point and eliminates local disease history around specific topographies as a confounding factor. This way, plot history becomes more important and factors associated with active management practices can be identified.

Naturally in a BBTD infested landscape, different farms would be at different levels of epidemy development linked to the landscape and management attributes but also to the age of the plantations. By choosing the replanted gardens, we had a unique opportunity to have multiple farms that started at the same time, with comparable management systems, not much cultivar variation and whose differences would bring us closer to understanding landscape and management-linked factors influencing BBTD prevalence.

ACTION: We revised text on lines 132-139: “ As all farms shared a known replanting start date with virus-free material, they provided a consistent baseline for assessing the environmental and management factors influencing BBTD reinfection. The presence of BBTD in the surrounding landscape ensured a comparable source of inoculum across sites, minimizing spatial bias that would otherwise occur in newly affected areas where infections remain localized in hotspots. By focusing on replanted fields, we eliminated the confounding effects of local disease history and topographic variation, allowing plot-level history and active management practices to emerge as the main explanatory factors.”

3. Based on the provided methodology, what specific measures were implemented to mitigate potential biases arising from subjective visual assessments of BBTD incidence during data collection?

RESPONSE: The data collection team was first trained on the implementation of the protocol in a one-week workshop outside of the data collection area. A uniform protocol with disease classification thresholds was used. BBTD symptoms follow progression from incipient levels (very easy to misdiagnose) to clearer ones at a later stage. Data taking involved cross-auditing a fraction of the gardens between data takers, evaluating the same mats previously classified.

We therefore believe the resulting bias would be random and end up in a general underestimation or overestimation. The research team had been trained in BBTD identification and had been used in the same community as trainers for two years. Our own research in Malawi, Benin, DRC and more recently in Rwanda showed that with minimal field training, farmers become quite accurate in detecting BBTD. A study done in Rwanda to assess performance and retention of BBTD identification knowledge a year after training showed practically the same result.

ACTION: Added after line 148-151: “The data collection team underwent standardized training using a uniform disease classification protocol, implemented cross-auditing of observations between data collectors, and leveraged experienced personnel previously trained and validated in accurate BBTD identification across multiple countries.”

Discussion:

1. What specific strategies were employed to ensure that the imputation process did not introduce bias or distort the true relationships within the dataset, particularly given the observed overdispersion and the eventual use of the NBRM?

Renata/Dato/

RESPONSE: Missing categorical variables were imputed using multiple imputation by chained equations (MICE). Multiple imputation generates several predictions for each missing value, allowing subsequent analyses to account for uncertainty in the imputations and produce accurate standard errors. When the observed data provide limited information about the missing values, imputations may vary widely, leading to larger standard errors, whereas highly predictive observed data yield more consistent imputations and smaller, yet still accurate, standard errors (Azur et al., 2011).

ACTION: We have added after line 178: To assess potential bias from imputation, we examined the standard errors of the imputed values.

2. How might the observed heteroscedasticity in the residuals and the small effect sizes of some statistically significant predictors in the best model (M1) be addressed to improve the practical utility and predictive power of future BBTD management strategies?

RESPONSE: We thank the reviewer for this insightful question.

ACTION: We added after line 369-374: “However, our analysis provides valuable insights into the shortcomings of some aspects of data availability. For example, the widest confidence interval ranges for the IRR were observed for the mean banana density at 30 m and 60 m. Designing an experimental protocol that captures greater variability (i.e., distance between rows and columns) could improve the practical utility and predictive power of the models for informing BBTD management strategies.”

3. What effect does promptly removing diseased banana plants (roguing) have on BBTV infection rates, and what evidence suggests a need for time-series studies to tease apart the intricate link between roguing frequency, reinfection, and overall disease dynamics?

RESPONSE: Prompt roguing slows down within field infection rates and slows down disease progress. Allen 1987, had indicated the link between disease appearance and infection acquisition by aphids, resulting in disease spread. In a separate study, we showed that consistent roguing could limit disease spread and promote recovery of fields previously under BBTD infestation in field experiments in Malawi, Benin and Burundi (Omondi 2020). Roguing also supported low risk seed availability for recovery within the fields. Roguing however may not prevent primary infection arriving into the field by seed or through immigrating aphids, hence the need for consistency and landscape scale application.

ACTION: We revised text on lines 445-450: “Roguing can significantly reduce BBTV infection rates, lowering incidence to 2-10% when applied consistently and early in the disease expression stage (Omondi et al., 2020). However, the emergence of new infections in both managed and non-managed farms followed a seasonal cycle (Omondi et al., 2020), highlighting the need for time-series studies to clarify how roguing frequency, disease pressure, and environmental factors interact to shape long-term BBTD dynamics..”

4. What are the ecological interactions among BBTV, its aphid vectors, the banana plants, and other possible host plants (including non-banana Zingiberales and vectors like P. caladii), and how do factors like cultivar susceptibility, aphid colonization, virus-driven changes in vector behavior, climate variation, plot size, seed/plant movement, and gender or resource access affect BBTV spread and epidemics?

RESPONSE: Studies have been carried out to asses the host diversity of banana aphids Pentalonia nigronervosa versus P. caladii. These include:

That both can complete their life cycles in various hosts other than banana and that P. caladii has a wider host range including banana; while P. nigronervosa tends to be limited to Musa spp (Foottit 2010; Wessels et al 2015). Both species are competent vectors of BBTV banana, but P. nigronervosa is more efficient. BBTV infection changes the host preference of non-viruliferous P. nigronervosa; but this preference is lost once the aphid acquires the virus (Muhurbaba et a., 2023; 2025). So there if virus manipulation that could accelerate the rate of spread in a landscape where the disease is present. Besides, we have shown recently that aphids growing on diseased plants multiply almost twice as fast as in healthy plants. Together these mean any presence of BBTV in the plots would strongly increase disease spread due to greater vector population output. However, for our study these factors are included in multiple dimensions including presence of aphids, density of bananas and presence of BBTD infested plants. While these are difficult to test in such a multiple scale, we note that these factors could affect observed model performance, such as significance of banana and of aphid numbers but not necessarily of BBTD infected plants.

ACTION: We added to Lines 389- 391: “The availability of viable BBTV susceptible hosts and the presence of backyard gardens could influence vector dispersal and virus spread dynamics. “

ACTION:

1. We change text on lines 458 from “Ecological interactions between the virus, vector, banana host, and other possible alternative host plants were relevant to this study.” to “Ecological interactions between the virus, vector, banana host, and other possible alternative host plants are highly relevant to understanding BBTD dynamics, but they are difficult to test under natural infection conditions due to their complexity.”

2. We also added after line 463: “Both P. caladii and P. nigronervosa can complete their life cycles on several hosts other than banana, although P. caladii has a broader host range, while P. nigronervosa is largely restricted to Musa spp. (Foottit, 2010; Wessels et al., 2015). Despite both being competent BBTV vectors, P. nigronervosa is the more efficient transmitter.

5. What are the primary drivers of Pentalonia nigronervosa population surges in Burundi-like landscapes? How far do viruliferous aphids disperse across typical smallholder mosaic landscapes, and which habitat features facilitate or hinder movement.

RESPONSE: To date, the actual range of aphid movement has not been measured directly. We have already added the following text the methods which partially covers the question: “The spread of BBTV through the vector is favoured by environmental factors favouring vector aphid population build up and migration, and rapid plant growth and accumulation of BBTV titre to enable vector acquisition. These include favourable temperature, wind and topography (Niyongere et al., 2013). Thus, the greatest BBTD risk BBTD has been associated with humid lowlands.” – Line 123

6. What are the practical implications of model averaging for interpreting BBTV risk in different agroecological zones, and how should field experiments be designed to identify context-specific drivers (e.g., aphid-driven vs. wind- or ant-mediated spread) to inform adaptive BBTD management strategies?

RESPONSE: Controlled experiments on cropping mixtures, non-host barriers and ant control have been suggested, to assess their effect on aphid distribution and disease spread. The possible role of non-host intercrops on natural enemies could also be similarly investigated; and preliminary results in our hands show that such complexity affects natural enemies in different ways. Ant studies would be interesting, especially in assessing the effects of repellents and biorational agents in BBTD control (such as soap and botanical pesticides). To date our experiments, show little effect on aphid presence, but the effects on ants would suggest a confounding relationship on aphids.

The field level management options presented here, could influence ants and aphids in diverse ways. Our focus is on their association with resultant disease appearance at this stage. Once the list of important associations is whittled down, systematic field tests could be done to validate their efficacy and come up with the best combination of cost-effective measures for BBTD management. This effort could field experimentation, parameter estimation from field experiments followed by scenario modelling (cf Mapinda et al., 2025).

ACTION: We rephrased the last sentence of the Discussion Line 503: “Field experiments targeting such context-specific factors by manipulating levels of cropping mixtures content, presence of non-host barriers or selective levels of aphid and ant control should be conducted to offer more precise insights and support adaptive management strategies for BBTD.”

7. Is there selection pressure on BBTV or vectors leading to more virulent strains or evasion of control measures?

RESPONSE: This is an interesting question and could require very specific studies. Indeed we know that the non-viruliferous aphids prefer infected plants; which also support faster growth of aphid populations (Adjalla et al., 2025; Muhurbuba et al, 2023; 2024). We also know that viruliferous aphids do not discriminate between infected and non-infected plants. Thus, in the presence of infected plants in a garden, there will be a net migration to spread the virus through unequal attraction and then equal dispersals.

A possible mechanism of selection for virulence could involve the delay of symptom appearance relative to the period of infectiousness of diseased plants. Allen (1987) established that three leaves would show symptoms before aphids begin migrating out and becoming an infection threat to other plants. This represents a period ranging from 3 weeks in young plantlets in the rainy season (rapid growth) to about six weeks in older plants Ngatat et al., (2017; 2022) and Chabi et al (2023) showed a variation in symptom appearance. Chabi also showed that transmission was always associated with symptom appearance such that banana aphids could not acquire BBTV from asymptomatic plants previously exposed toBBTV. As systemic virus, the duration of inoculation acquisition time and population multiplication on infected plants are important in BBTD transmission (Roudine et al 2023). So, evolution of invasion response could be strains showing symptoms much later when the virus titre can already be acquired and transmitted by aphids. This has not been studied yet but would be interesting.

ACTION: We have added after line 470-473:” BBTV infection can alter the host preference of non-viruliferous P. nigronervosa, an effect lost once the aphid acquires the virus (Muhurbaba et al., 2023; 2024), suggesting virus manipulation that could enhance transmission in endemic landscapes.”

Attachment Submitted filename: PLOS One reviewer response Final.docx

# Peer review: Decision Letter 1

**DOI:** 10.1371/journal.pone.0357834.r003

**Authors:** Sengottayan Senthil-Nathan

21 Jan 2026

Drivers of Banana Re-infection in Newly Planted Gardens in Banana Bunch top Disease Endemic Zones: Implications for the recovery of banana production

PLOS One

Dear Dr. Omondi,

Thank you for submitting your manuscript to PLOS ONE. After careful consideration, we feel that it has merit but does not fully meet PLOS ONE’s publication criteria as it currently stands. Therefore, we invite you to submit a revised version of the manuscript that addresses the points raised during the review process.

Please submit your revised manuscript by Mar 06 2026 11:59PM. If you will need more time than this to complete your revisions, please reply to this message or contact the journal office at plosone@plos.org. When you're ready to submit your revision, log on to https://www.editorialmanager.com/pone/ and select the 'Submissions Needing Revision' folder to locate your manuscript file.

Please include the following items when submitting your revised manuscript:

- A letter that responds to each point raised by the academic editor and reviewer(s). You should upload this letter as a separate file labeled 'Response to Reviewers'.
- A marked-up copy of your manuscript that highlights changes made to the original version. You should upload this as a separate file labeled 'Revised Manuscript with Track Changes'.
- An unmarked version of your revised paper without tracked changes. You should upload this as a separate file labeled 'Manuscript'.

If you would like to make changes to your financial disclosure, please include your updated statement in your cover letter. Guidelines for resubmitting your figure files are available below the reviewer comments at the end of this letter.

If applicable, we recommend that you deposit your laboratory protocols in protocols.io to enhance the reproducibility of your results. Protocols.io assigns your protocol its own identifier (DOI) so that it can be cited independently in the future. For instructions see: https://journals.plos.org/plosone/s/submission-guidelines#loc-laboratory-protocols. Additionally, PLOS ONE offers an option for publishing peer-reviewed Lab Protocol articles, which describe protocols hosted on protocols.io. Read more information on sharing protocols at https://plos.org/protocols?utm_medium=editorial-email&utm_source=authorletters&utm_campaign=protocols.

We look forward to receiving your revised manuscript.

Kind regards,

Sengottayan Senthil-Nathan, Ph D

Academic Editor

PLOS One

Journal Requirements:

If the reviewer comments include a recommendation to cite specific previously published works, please review and evaluate these publications to determine whether they are relevant and should be cited. There is no requirement to cite these works unless the editor has indicated otherwise.

Additional Editor Comments:

Reviewer(s) has serious concern with statistical analysis. Please address the issue

[Note: HTML markup is below. Please do not edit.]

Reviewers' comments:

Reviewer's Responses to Questions

**Comments to the Author**

Reviewer #1: All comments have been addressed

Reviewer #2: (No Response)

Reviewer #3: (No Response)

**********

2. Is the manuscript technically sound, and do the data support the conclusions?

Reviewer #1: Yes

Reviewer #2: No

Reviewer #3: Partly

**********

3. Has the statistical analysis been performed appropriately and rigorously?

Reviewer #1: Yes

Reviewer #2: No

Reviewer #3: Yes

**********

4. Have the authors made all data underlying the findings in their manuscript fully available?

The PLOS Data policy

Reviewer #1: Yes

Reviewer #2: No

Reviewer #3: Yes

**********

5. Is the manuscript presented in an intelligible fashion and written in standard English?

Reviewer #1: Yes

Reviewer #2: No

Reviewer #3: No

**********

Reviewer #1: (No Response)

Reviewer #2: The current study investigated how environmental and management factors affect Banana Bunchy Top Disease (BBTD) infection in banana plants using a unique on-farm setup where virus-free tissue-cultured plantlets have been grown for three years before the investigation. The outcome of this study, if analyzed properly, is expected to provide practical and important insights into banana production. Unfortunately, however, I am not able to recommend this manuscript for publication in PLOS ONE due to several major concerns stated below.

1. Exclusion of spatial autocorrelation analysis

A virus disease mediated by an insect vector such as BBTD is expected to cluster around the point of vector landing, making spatial autocorrelation analysis quite reasonable. The current study reported that Moran's I test detected no significant spatial autocorrelation in BBTD infection, based on which the spatial autocorrelation term was excluded from the regression equation. However, it is undeniable that the insignificant spatial autocorrelation was likely caused by the use of a 10 km threshold in the spatial weight matrix, which is logically impossible to justify in the context of the current study—especially given that the authors cite Allen (1978) for a 90m vector dispersal range in their buffer zone design. This represents a fundamental inconsistency: if vector dispersal is limited to 90m, why would spatial dependence be assessed at 10km?

Furthermore, while the process of model construction was excessively described in the Materials and Methods section (including full mathematical formulas for AIC, AICc, and Akaike weights), the type and construction method of the spatial weight matrix was not described at all. This raises serious doubts about the authors' understanding of the analytical techniques. The lack of appropriate citations to spatial econometrics and spatial analysis literature adds to this concern.

2. Unbalanced descriptions of analytical techniques

The unnecessarily redundant description of model construction implies the importance of this process in the current study. However, the Abstract contains only one sentence about this analysis: "Model selection indicated that aphid prevalence, banana density, rogueing frequency, and seed source appeared in more than half of the best-fit models..." This phrasing is problematic because "model selection" (a process) cannot logically "indicate" that variables "appeared" in models. The correct subject should be "the selected models" or "model averaging." This error, combined with the extensive but poorly integrated methodological description, suggests insufficient understanding of the analytical framework.

The disconnect between the space devoted to methodological detail in the Methods section and the minimal mention in the Abstract is difficult to understand and suggests that different sections were written without adequate integration.

3. Inappropriate presentation and interpretation of parameter estimates

The Results section describes only the limitations of the model rather than the actual results of parameter estimation. The authors note "small effect sizes or wide confidence intervals," "heteroscedasticity," and "non-random structure in residuals," yet proceed to base their Discussion on this problematic model without adequately addressing whether the conclusions can be trusted given these issues.

More concerning, the Discussion section attempts to interpret results of parameter estimation without citing any specific tables, figures, coefficient estimates, or confidence intervals. For example, statements like "Lower infection levels were associated with factors such as..." lack any quantitative support or table references. This is particularly problematic given that Tables 4 and 5 contain the coefficient estimates that should form the basis of interpretation.

This unusual structure—excessive methodological detail, minimal results reporting, and unsupported discussion—raises fundamental concerns about whether the authors can adequately defend their analytical approach and interpret their findings.

Overall recommendation

The combination of these issues—inappropriate spatial analysis using an unjustifiable 10km threshold, inconsistent manuscript structure with poor integration between sections, fundamental misuse of statistical terminology, and inadequate interpretation of results—prevents me from recommending this manuscript for publication.

Reviewer #3: Following a large-scale tissue culture based replacement of banana plants in Burundi, the authors evaluated the status of plants 3 years later and gathered information on a number of factors related to banana fields, their surroundings, seed sourcing, and other vegetation present. Based on the reinfection of these ‘clean farms’, the authors use this information to build a negative binomial generalized linear model with BBTD incidence as the response variable.

Field data at scale on this subject is rare, so the manuscript has value for the plant pathology community. I can also see that the authors have already responded to one review and made efforts to improve the manuscript. However there are still issues with the manuscript that need to be addressed before it can be considered for publication.

Some of the methods are insufficiently detailed to be replicable. The study site section gives no description of the size of communes or distance. A map would have been useful. Without some description it is not possible for the reader to evaluate the suitability of sample selection, as well as the use of some of the geographic analysis methods (for example the use of a 10km distance threshold in lines 204-205).

The description of sampling for BBTD is also unclear. In the methods you describe ‘In each target field, two diagonal transect lines were laid along which plants were sampled at regular intervals to cover all quarters of the plot. Every third plant was assessed to determine BBTD incidence and aphid density, along two transverse transects, to cover all quarters of the garden.’ In Table 1 aphid density is not reported, only presence/absence. And in Figure 1 you show concentric square areas with subplot labels that don’t seem to match anything else in the manuscript. If you did actually lay out your plots this way on a square rather than a circle, the points where the diagonal grey lines cross into the plots are actually further than 30, 60, 90m. Either way, the description needs clarification.

There are lots of broken citation & in-text reference links in the revised version, and a lot of spelling and punctuation errors. I am attaching a PDF with many examples highlighted, but you need to go through the whole manuscript carefully.

In addition to the PDF, please find specific points below:

1) Please check authors' names are entered correctly in the system; this appears to include a mix of lastname-firstname and firstname-lastname.

2) 48-49: ‘The banana fruit and various plant parts are consumed in multiple forms depending on the variety.’ You then go on to only give examples of the fruit – either give examples of other plant parts or leave this out.

3) 80-82: ‘Small-holder production systems, dominated by local landraces, are particularly vulnerable because of their dependence on informal seed sourcing and the perennial low-input nature of the production system (Simbare et al., 2020)’ Please clarify the link between being low-input and vulnerability to BBTD.

4) 100-101: ‘The recovery strategy deployed in Burundi offered a case study to monitor reinfection in 121 small farms and two community nurseries for tissue culture plants in four villages.’ The way you have written this suggests you monitored reinfection in tissue culture nurseries. Please clarify.

5) 137-139: ‘By focusing on replanted fields, we eliminated the confounding effects of local disease history and topographic variation, allowing plot-level history and active management practices to emerge as the main explanatory factors’. Explain how this eliminates the effect of topography?

6) 168: So you collected gender but did not include it in the analysis? How was this determined in the majority case in which the owner is in a married couple?

7) ‘Farm communities in the Rift Valley lost fields of the previously dominant beer banana variety, ‘Kayinja’, to Fusarium wilt race1.’ This is an empirical claim, and since it’s in the introduction we assume it is not a results of the present work. Please cite.

8) The manuscript does not seem sure whether altitude was included in the model or not. Please review the following conflicting statements and correct the manuscript:

o 9-10: ‘In contrast, higher altitude, the presence of hedgerows, and frequent rogueing were negatively associated with BBTD.’

o 350-352: ‘Additionally, variations in the effectiveness of BBTD control measures may be influenced by context specific environmental factors, such as altitude, temperature, and seasonal conditions, which were not fully accounted for in the model.’

o Altitude not included in table 1.

9) Given that it is clearly one of the most important factors in your study, the seed variable is quite basic, and is treated very loosely and vaguely. In Table 1 the SeedS variable is presented as binary: either received seeds other than the project-provided, or not. Lines 433-34 directly interpret this as meaning ‘from informal or local sources’. Was this the case, or did the variable also include other formal sources? In Table 4 it is presented as ‘MFN - Never planting material received or collected from neighbours unknown’. Conclusions line 515 simply says ‘the importance of seed systems’. The manuscript needs extensive revision on this point to present a harmonized description throughout and provide clarity on what was actually collected here. Also consider explaining to the reader what the planting material is and some background about what’s known of its exchange in the study region.

10) Lines 321-325: among the factors associated with # of BBTD affected plants you include ‘presence of vegetation edges towards the east’ – do you have a hypothesis for why the east and no other direction? It is hard for the reader to understand your choice to include vegetation on each cardinal direction as independent variables in the model, and then when only one of them was significant you conclude that adjacent vegetation is an important management/environmental factor. Please revise how you handle this and justify its inclusion.

11) There also seems to be a contradiction in the authors’ conclusions about the effect of neighboring plots:

o Lines 381-384: ‘Notably, BBTD levels in neighboring gardens were not among the strongest predictors of infection in the target plots. This emphasizes the importance of broader community-scale management of isolated field-level interventions.’

o Then 387-390: ‘Banana canopy density around the recovered plot (connectivity between target plot and neighbouring plots) were associated with an increased BBTD risk in the target plot. These variables could influence the spread of vector aphids in the BBTD between mats and gardens (Kebede et al., 2018).

12) Lines 390-391: It is not at all clear what you mean here, or how a plant species 'intervenes'

13) Lines 393-394: Not clear what ‘the most considered BBTD management options’ means, and a single 50-year old citation is not the best choice if you are trying to refer to the whole period since then, unless you are saying there has been no progress?

14) Lines 394-395: ‘We observed that hedges around banana gardens were associated with lower levels of infection’ Do hedges count as ‘vegetation’ and if so their presence on the east should be associated with higher levels? What is the difference between a hedge and vegetation?

15) Lines 422-23: This passage mentions the use of ELISA to test plants for BBTV. This was not mentioned in the methods section.

16) The weakest part of the manuscript is the conclusions (lines 511-526). It does a poor job of summarizing the main results and is written in very imprecise language. The section needs comprehensive revision to make sure that each sentence is making a clear and specific point drawing from the results. Some sections simply do not make sense together. See some examples:

o Lines 512-514: ‘Three key factors in BBTD re-introduction into clean fields were revealed landscape complexity, farmers’ seed movement, and host density, which can be consistently manipulated to achieve sustainable control.’ This statement is too general and extrapolates beyond what you showed in this study. You have looked in a limited area, and the study only showed correlations of these factors with lower BBTD levels. There was no analysis of sustainability thresholds or attempt to apply the concepts beyond the study site. Please rewrite this section of the conclusion with more caution in expressing the conclusions.

o 516-519: ‘The success of these approaches may vary significantly according to latitude (or location) and weather conditions. Thus, common farmer cropping practices, such as intercropping and strip cropping, can be used to retard BBTD spread.’ The second sentence does not follow logically from the first, and the study contains no evidence that the second claim is true as it did not evaluate intercropping or strip cropping.

o 519-520: ‘Similarly, seed acquisition practices and direct removal of BBTD-affected plants are essential and available to farmers.’ It is unclear what ‘seed acquisition practices’ means, and the sentence is grammatically incorrect.

o 523-524: ‘To the best of our knowledge, this is the first study to evaluate the spatial implications of farmer choices on BBTD’. The terms ‘the spatial implications’ and ‘farmer choices on BBTD’ are too vague and imprecise to give this sentence any meaning.

17) 535-526: ‘Importantly, this underscores the need to consider contextual factors in epidemiological modelling and disease control efforts in Africa.’ Is this really the main finding of your study? If so it is quite weak. I don’t think you will find a single pathologist that is not already doing this. Please consider ending with something more specific to your findings.

18) In the ethical statement you have written that the study did not involve humans as it was based on field observation. However this is not strictly true, as you include survey information included (for example gender, rogueing frequency, and seed sourcing behavior). Please revise.

**********

what does this mean?). If published, this will include your full peer review and any attached files.

If you choose “no”, your identity will remain anonymous but your review may still be made public.

**Do you want your identity to be public for this peer review?**  For information about this choice, including consent withdrawal, please see our Privacy Policy

Reviewer #1: No

Reviewer #2: **Yes:** Nobuhito Sekiya

Reviewer #3: No

**********

[NOTE: If reviewer comments were submitted as an attachment file, they will be attached to this email and accessible via the submission site. Please log into your account, locate the manuscript record, and check for the action link "View Attachments". If this link does not appear, there are no attachment files.]

To ensure your figures meet our technical requirements, please review our figure guidelines: https://journals.plos.org/plosone/s/figures

You may also use PLOS’s free figure tool, NAAS, to help you prepare publication quality figures: https://journals.plos.org/plosone/s/figures#loc-tools-for-figure-preparation.

NAAS will assess whether your figures meet our technical requirements by comparing each figure against our figure specifications.

Attachment Submitted filename: PONE-D-25-42829R2.pdf

# Author comment: Author response to Decision Letter 2

**DOI:** 10.1371/journal.pone.0357834.r004

12 Mar 2026

Dear Reviewers,

We are grateful for the effort made to improve our manuscript. We were in most cases grateful for your comments and followed the recommended corrections or made an effort to express our message more clearly. In these cases, we give a response and also the action taken, referring to the 'track changes' version of the article. Where we disagreed, we explained and in most cases, also attempted to express ourselves more clearly. Below is a consolidated list of the your concerns and our responses:

6. Review Comments to the Author

Please use the space provided to explain your answers to the questions above. You may also include additional comments for the author, including concerns about dual publication, research ethics, or publication ethics. (Please upload your review as an attachment if it exceeds 20,000 characters)

Reviewer #1: (No Response)

Reviewer #2: The current study investigated how environmental and management factors affect Banana Bunchy Top Disease (BBTD) infection in banana plants using a unique on-farm setup where virus-free tissue-cultured plantlets have been grown for three years before the investigation. The outcome of this study, if analyzed properly, is expected to provide practical and important insights into banana production. Unfortunately, however, I am not able to recommend this manuscript for publication in PLOS ONE due to several major concerns stated below.

Response: We thank the reviewer for this observation. We attach a revised version of our manuscript, and a response, which includes a to the concerns of the reviewers and action taken to communicate more clearly where necessary. We hope this version has significantly improved the clarity with which we communicate our results.

1. Exclusion of spatial autocorrelation analysis

A virus disease mediated by an insect vector such as BBTD is expected to cluster around the point of vector landing, making spatial autocorrelation analysis quite reasonable. The current study reported that Moran's I test detected no significant spatial autocorrelation in BBTD infection, based on which the spatial autocorrelation term was excluded from the regression equation. However, it is undeniable that the insignificant spatial autocorrelation was likely caused by the use of a 10 km threshold in the spatial weight matrix, which is logically impossible to justify in the context of the current study—especially given that the authors cite Allen (1978) for a 90m vector dispersal range in their buffer zone design. This represents a fundamental inconsistency: if vector dispersal is limited to 90m, why would spatial dependence be assessed at 10km?

Furthermore, while the process of model construction was excessively described in the Materials and Methods section (including full mathematical formulas for AIC, AICc, and Akaike weights), the type and construction method of the spatial weight matrix was not described at all. This raises serious doubts about the authors' understanding of the analytical techniques. The lack of appropriate citations to spatial econometrics and spatial analysis literature adds to this concern. –

Response: We thank the reviewer for raising this important point regarding spatial dependence and model specification. We agree that BBTD, as a vector-borne disease, can exhibit clustering at fine spatial scales associated with aphid-mediated dispersal. However, the objective of our spatial autocorrelation analysis was not to model plot-level vector dispersal processes, but rather to evaluate whether between-site dependence existed at the landscape scale relevant to our regression framework.

Our sampling units were spatially discrete fields distributed across a broader landscape, with inter-site distances typically far exceeding known aphid dispersal distances. The regression models were constructed at the site level, not at the within-field plant scale. Therefore, the relevant question for model specification was whether infection intensity at one surveyed site was statistically dependent on infection intensity at neighbouring surveyed sites.

We used a 10 km threshold in the spatial weights matrix to test for potential landscape-level spatial structure among surveyed fields. This threshold was not intended to represent vector dispersal distance; rather, it was selected to ensure that each site had a sufficient number of neighbouring sites in the weights matrix for stable estimation of Moran’s I, given the spatial configuration of the survey. Importantly, most site-to-site distances were substantially larger than aphid dispersal distances, meaning that direct epidemiological coupling between sites via short-range vector movement is biologically implausible. Thus, the spatial test was designed to detect broader landscape-level spatial dependence (e.g., shared agroecological or management contexts), not fine-scale vector clustering.

To ensure that the result was not an artefact of the 10 km threshold, we conducted sensitivity analyses using 15 km and 20 km distance bands. In all cases, Moran’s I remained near zero and non-significant. This confirms that landscape-level spatial dependence is absent and that our inference is not dependent on the chosen neighbourhood specification.

The absence of significant global or local spatial autocorrelation indicates that infection counts at surveyed sites were not spatially structured at the landscape scale considered. Under these conditions, inclusion of conditional autoregressive terms or geostatistical random fields would not be statistically justified and could introduce unnecessary model complexity and overparameterization. We have clarified this distinction in the revised manuscript and now explicitly describe the construction of the spatial weights matrix and the rationale for the chosen threshold.

We also note that our use of negative binomial regression addressed overdispersion in infection counts, and VIF diagnostics ensured limited multicollinearity among predictors. Given the absence of detectable landscape-level spatial dependence, the adopted modelling framework remains appropriate for inference at the site scale.

Action: We have revised the Methods section to clarify these points lines ( 215-246).

2. Unbalanced descriptions of analytical techniques

The unnecessarily redundant description of model construction implies the importance of this process in the current study. However, the Abstract contains only one sentence about this analysis: "Model selection indicated that aphid prevalence, banana density, rogueing frequency, and seed source appeared in more than half of the best-fit models..." This phrasing is problematic because "model selection" (a process) cannot logically "indicate" that variables "appeared" in models. The correct subject should be "the selected models" or "model averaging." This error, combined with the extensive but poorly integrated methodological description, suggests insufficient understanding of the analytical framework.

The disconnect between the space devoted to methodological detail in the Methods section and the minimal mention in the Abstract is difficult to understand and suggests that different sections were written without adequate integration.

Response: We appreciate this comment; and acknowledge a possible weakness in the summary initially provided.

Action: the abstract has now been redone for better coherence (line 3-19)

3. Inappropriate presentation and interpretation of parameter estimates

The Results section describes only the limitations of the model rather than the actual results of parameter estimation. The authors note "small effect sizes or wide confidence intervals," "heteroscedasticity," and "non-random structure in residuals," yet proceed to base their Discussion on this problematic model without adequately addressing whether the conclusions can be trusted given these issues.

Response: Thank you for this comment. We have revised the Results section to explicitly present the parameter estimates, standard errors, 95% confidence intervals, and Incidence Rate Ratios (IRRs) for all key predictors (Tables 3–4). We have also contextualized coefficients according to the observed ranges of continuous variables, so that seemingly small coefficients (e.g., plot size) are interpreted in terms of their practical effect across the observed variable range. For categorical variables, we now clearly report the percent change in expected infection counts relative to reference categories. These changes ensure that both the magnitude and direction of effects are interpretable and provide a robust basis for discussion and management recommendations.

More concerning, the Discussion section attempts to interpret results of parameter estimation without citing any specific tables, figures, coefficient estimates, or confidence intervals. For example, statements like "Lower infection levels were associated with factors such as..." lack any quantitative support or table references. This is particularly problematic given that Tables 4 and 5 contain the coefficient estimates that should form the basis of interpretation.

This unusual structure—excessive methodological detail, minimal results reporting, and unsupported discussion—raises fundamental concerns about whether the authors can adequately defend their analytical approach and interpret their findings.

We appreciate this comment. In line with the above revision, we believe the description of the results is now clear enough.

Action: The discussion has been entirely rewritten to accommodate changes in the presentation and interpretation of results considering the Reviewers’ comments (lines 427-637)

Overall recommendation

The combination of these issues—inappropriate spatial analysis using an unjustifiable 10km threshold, inconsistent manuscript structure with poor integration between sections, fundamental misuse of statistical terminology, and inadequate interpretation of results—prevents me from recommending this manuscript for publication.

Response: We are grateful for the comments and views proposed, and have considered them in the new revised version of our manuscript. We wish to invite the reviewer to look again at our revision, which we believe has addressed the issues raised. We look forward to further discussion on this.

Reviewer #3: Following a large-scale tissue culture based replacement of banana plants in Burundi, the authors evaluated the status of plants 3 years later and gathered information on a number of factors related to banana fields, their surroundings, seed sourcing, and other vegetation present. Based on the reinfection of these ‘clean farms’, the authors use this information to build a negative binomial generalized linear model with BBTD incidence as the response variable.

Field data at scale on this subject is rare, so the manuscript has value for the plant pathology community. I can also see that the authors have already responded to one review and made efforts to improve the manuscript. However there are still issues with the manuscript that need to be addressed before it can be considered for publication.

Some of the methods are insufficiently detailed to be replicable. The study site section gives no description of the size of communes or distance. A map would have been useful. Without some description it is not possible for the reader to evaluate the suitability of sample selection, as well as the use of some of the geographic analysis methods (for example the use of a 10km distance threshold in lines 204-205).

We are grateful for these observations and

Action: We have added a map of the study area (Figure 1); and now renamed the other Figures.

The description of sampling for BBTD is also unclear. In the methods you describe ‘In each target field, two diagonal transect lines were laid along which plants were sampled at regular intervals to cover all quarters of the plot. Every third plant was assessed to determine BBTD incidence and aphid density, along two transverse transects, to cover all quarters of the garden.’ In Table 1 aphid density is not reported, only presence/absence. And in Figure 1 you show concentric square areas with subplot labels that don’t seem to match anything else in the manuscript. If you did actually lay out your plots this way on a square rather than a circle, the points where the diagonal grey lines cross into the plots are actually further than 30, 60, 90m. Either way, the description needs clarification.

We appreciate this comment. Aphids were not counted individually but rather in density classes ranging from 0 (absent) 1 (single colonies) 2 (large multiple adult colonies) to 4 large (mixed colonies with alates). Regarding the map, a perfectly uniform distance from the target plot is only possible with circular plots or those with smoothed out corners. But on the ground, many plots are usually regular (trapezoid, or qyadrilateral). We welcome any alternative presentation of the sampling unit.

Action: The reference to aphid density classes is maintained (line 172)

There are lots of broken citation & in-text reference links in the revised version, and a lot of spelling and punctuation errors. I am attaching a PDF with many examples highlighted, but you need to go through the whole manuscript carefully.

We have again gone through the manuscript and corrected the corrections desired.

We appreciate this comment.

Action: We have used the PDF version to correct the errors highlighted. We have also removed hyperlinks to table titles and Figure legends which were generating ‘error reference’ text in the manuscript. Besides we have carefully reread and run a spell check

In addition to the PDF, please find specific points below:

1) Please check authors' names are entered correctly in the system; this appears to include a mix of lastname-firstname and firstname-lastname.

Action: This has now been aligned, all authors are cited in firstname-lastname Format.

2) 48-49: ‘The banana fruit and various plant parts are consumed in multiple forms depending on the variety.’ You then go on to only give examples of the fruit – either give examples of other plant parts or leave this out.

Response: Agreed:

Action: Modified to leave out other plant parts from consumption and added the use of leaves in food preparation (Lines 52-59).

3) 80-82: ‘Small-holder production systems, dominated by local landraces, are particularly vulnerable because of their dependence on informal seed sourcing and the perennial low-input nature of the production system (Simbare et al., 2020)’ Please clarify the link between being low-input and vulnerability to BBTD.

Response: We are grateful for this observation.

Action: The sentence has been clarified to specifically highlight longevity of plantations, seed systems vulnerability and effect of low monitoring or replacement on BBTD (line 83-90).

4) 100-101: ‘The recovery strategy deployed in Burundi offered a case study to monitor reinfection in 121 small farms and two community nurseries for tissue culture plants in four villages.’ The way you have written this suggests you monitored reinfection in tissue culture nurseries. Please clarify.

OK the sentence has been revised. There was no reinfection in tissue culture nurseries (confirmed by symptom and ELISA testing before distribution). Monitoring was done on the fields, three years after planting.

5) 137-139: ‘By focusing on replanted fields, we eliminated the confounding effects of local disease history and topographic variation, allowing plot-level history and active management practices to emerge as the main explanatory factors’. Explain how this eliminates the effect of topography?

Action: This sentence has been reframed for better clarify (Lines: 144-149)

6) 168: So you collected gender but did not include it in the analysis? How was this determined in the majority case in which the owner is in a married couple?

Response: This observation is correct. The group surveyed was one that was in partnership with ISABU/Bioversity BBTD management project in Burundi. Each member (even where there were spouses) had at least one farm they were responsible for and which was included and onitored in the project to determine, for example, if the f

Attachment Submitted filename: Omondi et alRevision.docx

# Peer review: Decision Letter 2

**DOI:** 10.1371/journal.pone.0357834.r005

**Authors:** Sengottayan Senthil-Nathan

10 Jun 2026

Dear Dr. Omondi,

Thank you for submitting your manuscript to PLOS ONE. After careful consideration, we feel that it has merit but does not fully meet PLOS ONE’s publication criteria as it currently stands. Therefore, we invite you to submit a revised version of the manuscript that addresses the points raised during the review process.

Please submit your revised manuscript by Jul 25 2026 11:59PM. If you will need more time than this to complete your revisions, please reply to this message or contact the journal office at plosone@plos.org. When you're ready to submit your revision, log on to https://www.editorialmanager.com/pone/ and select the 'Submissions Needing Revision' folder to locate your manuscript file.

- A letter that responds to each point raised by the academic editor and reviewer(s). You should upload this letter as a separate file labeled 'Response to Reviewers'.
- A marked-up copy of your manuscript that highlights changes made to the original version. You should upload this as a separate file labeled 'Revised Manuscript with Track Changes'.
- An unmarked version of your revised paper without tracked changes. You should upload this as a separate file labeled 'Manuscript'.

If you would like to make changes to your financial disclosure, please include your updated statement in your cover letter. Guidelines for resubmitting your figure files are available below the reviewer comments at the end of this letter.

If applicable, we recommend that you deposit your laboratory protocols in protocols.io to enhance the reproducibility of your results. Protocols.io assigns your protocol its own identifier (DOI) so that it can be cited independently in the future. For instructions see: https://journals.plos.org/plosone/s/submission-guidelines#loc-laboratory-protocols. Additionally, PLOS ONE offers an option for publishing peer-reviewed Lab Protocol articles, which describe protocols hosted on protocols.io. Read more information on sharing protocols at https://plos.org/protocols?utm_medium=editorial-email&utm_source=authorletters&utm_campaign=protocols.

As the corresponding author, your ORCID iD is verified in the submission system and will appear in the published article. PLOS supports the use of ORCID, and we encourage all coauthors to register for an ORCID iD and use it as well. Please encourage your coauthors to verify their ORCID iD within the submission system before final acceptance, as unverified ORCID iDs will not appear in the published article. *Only*  the individual author can complete the verification step; PLOS staff *cannot*  verify ORCID iDs on behalf of authors.

We look forward to receiving your revised manuscript.

Kind regards,

Sengottayan Senthil-Nathan, D.Sc

Academic Editor

PLOS One

Journal Requirements:

If the reviewer comments include a recommendation to cite specific previously published works, please review and evaluate these publications to determine whether they are relevant and should be cited. There is no requirement to cite these works unless the editor has indicated otherwise.

Please review your reference list to ensure that it is complete and correct. If you have cited papers that have been retracted, please include the rationale for doing so in the manuscript text, or remove these references and replace them with relevant current references. Any changes to the reference list should be mentioned in the rebuttal letter that accompanies your revised manuscript. If you need to cite a retracted article, indicate the article’s retracted status in the References list and also include a citation and full reference for the retraction notice.

Additional Editor Comments:

Comments

1. Causality vs. Association: Clarify Interpretation

The abstract and discussion repeatedly use causal language (“driving”, “positively associate with”, “influencing infection”), but the study design is cross-sectional and observational. While multivariate modeling (e.g., GLM or random forest) likely underpins the reported associations, the manuscript does not explicitly state:

•Which statistical models were used (and justification for selection),

•Whether spatial autocorrelation was tested/accounted for (gardens may be clustered geographically, violating independence assumptions),

•Whether confounding (e.g., soil type, irrigation practices, proximity to infected fallow fields) was assessed or adjusted for.

Suggestion: Revise language to consistently reflect associations, not causation (e.g., “were associated with higher BBTD prevalence”). Add a brief methods subsection on model specification, diagnostics (VIF, residuals, spatial tests), and handling of potential confounders.

2. The aphid quantification method lacks detail

The manuscript states “increased aphid populations” correlate with BBTD, yet it does not specify:

•How aphids were sampled (visual counts? yellow traps? leaf inspections?),

•Timing and frequency (single snapshot vs. seasonal monitoring),

•Whether Pentalonia nigronervosa (the sole BBTV vector) was confirmed morphologically or molecularly, or whether non-vector aphids were inadvertently included.

Suggestion: Clarify sampling protocol in Methods (ideally with a citation or standard protocol reference, e.g., IITA or FAO guidelines). If P. nigronervosa identification wasn’t verified, explicitly acknowledge this as a limitation.

Identification wasn’t verified; explicitly acknowledge this as a limitation.

3. Rogueing definition and fidelity are ambiguous

“Frequent rogueing” is cited as protective, but no definition is provided:

•Was it self-reported by farmers? Observed? Documented in logs?

•What constituted “rogueing” — removal of symptomatic plants only? Or also asymptomatic neighbors (a recommended practice)?

•Was timing (e.g., pre- or post-symptom onset) recorded?

Suggestion: Define rogueing operationally in Methods and discuss the reliability of measurement (e.g., inter-farmer variability, recall bias). Consider adding a brief validation note if field staff verified reports.

4. Lack of BBTV detection confirmation

The study assumes BBTD diagnosis = BBTV infection. However, visual symptoms can mimic other stresses (e.g., nematode damage, drought, nutrient deficiency). No mention is made of:

• Diagnostic confirmation (e.g., PCR, ELISA, or lateral flow assays) on a subsample,

• Inter-rater reliability of symptom scoring (if multiple enumerators were used).

Suggestion: State whether lab confirmation was performed — and if not, add a clear limitation acknowledging possible misclassification bias, which may attenuate or distort observed associations.

[Note: HTML markup is below. Please do not edit.]

Reviewers' comments:

Reviewer's Responses to Questions

**Comments to the Author**

Reviewer #1: All comments have been addressed

**********

2. Is the manuscript technically sound, and do the data support the conclusions?

Reviewer #1: Yes

**********

3. Has the statistical analysis been performed appropriately and rigorously?

Reviewer #1: Yes

**********

4. Have the authors made all data underlying the findings in their manuscript fully available?

The PLOS Data policy

Reviewer #1: Yes

**********

5. Is the manuscript presented in an intelligible fashion and written in standard English?

Reviewer #1: Yes

**********

Reviewer #1: Strengths

•Timely and applied relevance: BBTD remains a critical constraint to food security and livelihoods in smallholder banana systems across Africa and Asia. This study addresses a practical, underexplored question: why do virus-free plantings still fail?

•Strong field design: The assessment of 121 independently managed gardens — all established with certified virus-free plantlets — provides valuable real-world context beyond controlled trials.

•Ecologically grounded variables: Inclusion of landscape-level factors (elevation, hedgerows, varietal diversity, rogueing frequency) reflects a systems-thinking approach aligned with agroecological disease management principles.

•Clear translational messaging: Recommendations (e.g., barrier plants, mixed cropping, community rogueing protocols) are actionable and farmer-informed — highly appropriate for PLOS ONE’s mission of accessible, policy-relevant science.

Comments

1. Causality vs. Association: Clarify Interpretation

The abstract and discussion repeatedly use causal language (“driving”, “positively associate with”, “influencing infection”), but the study design is cross-sectional and observational. While multivariate modeling (e.g., GLM or random forest) likely underpins the reported associations, the manuscript does not explicitly state:

•Which statistical models were used (and justification for selection),

•Whether spatial autocorrelation was tested/accounted for (gardens may be clustered geographically, violating independence assumptions),

•Whether confounding (e.g., soil type, irrigation practices, proximity to infected fallow fields) was assessed or adjusted for.

Suggestion: Revise language to consistently reflect associations, not causation (e.g., “were associated with higher BBTD prevalence”). Add a brief methods subsection on model specification, diagnostics (VIF, residuals, spatial tests), and handling of potential confounders.

2. The aphid quantification method lacks detail

The manuscript states “increased aphid populations” correlate with BBTD, yet it does not specify:

•How aphids were sampled (visual counts? yellow traps? leaf inspections?),

•Timing and frequency (single snapshot vs. seasonal monitoring),

•Whether Pentalonia nigronervosa (the sole BBTV vector) was confirmed morphologically or molecularly, or whether non-vector aphids were inadvertently included.

Suggestion: Clarify sampling protocol in Methods (ideally with a citation or standard protocol reference, e.g., IITA or FAO guidelines). If P. nigronervosa identification wasn’t verified, explicitly acknowledge this as a limitation.

Identification wasn’t verified; explicitly acknowledge this as a limitation.

3. Rogueing definition and fidelity are ambiguous

“Frequent rogueing” is cited as protective, but no definition is provided:

•Was it self-reported by farmers? Observed? Documented in logs?

•What constituted “rogueing” — removal of symptomatic plants only? Or also asymptomatic neighbors (a recommended practice)?

•Was timing (e.g., pre- or post-symptom onset) recorded?

Suggestion: Define rogueing operationally in Methods and discuss the reliability of measurement (e.g., inter-farmer variability, recall bias). Consider adding a brief validation note if field staff verified reports.

4. Lack of BBTV detection confirmation

The study assumes BBTD diagnosis = BBTV infection. However, visual symptoms can mimic other stresses (e.g., nematode damage, drought, nutrient deficiency). No mention is made of:

• Diagnostic confirmation (e.g., PCR, ELISA, or lateral flow assays) on a subsample,

• Inter-rater reliability of symptom scoring (if multiple enumerators were used).

Suggestion: State whether lab confirmation was performed — and if not, add a clear limitation acknowledging possible misclassification bias, which may attenuate or distort observed associations.

**********

what does this mean?). If published, this will include your full peer review and any attached files.

If you choose “no”, your identity will remain anonymous but your review may still be made public.

**Do you want your identity to be public for this peer review?**  For information about this choice, including consent withdrawal, please see our Privacy Policy

Reviewer #1: No

**********

[NOTE: If reviewer comments were submitted as an attachment file, they will be attached to this email and accessible via the submission site. Please log into your account, locate the manuscript record, and check for the action link "View Attachments". If this link does not appear, there are no attachment files.]

To ensure your figures meet our technical requirements, please review our figure guidelines: https://journals.plos.org/plosone/s/figures

You may also use PLOS’s free figure tool, NAAS, to help you prepare publication quality figures: https://journals.plos.org/plosone/s/figures#loc-tools-for-figure-preparation.

NAAS will assess whether your figures meet our technical requirements by comparing each figure against our figure specifications.

# Author comment: Author response to Decision Letter 3

**DOI:** 10.1371/journal.pone.0357834.r006

30 Jul 2026

PONE-D-25-42829R2

Drivers of Banana Re-infection in Newly Planted Gardens in Banana Bunchy top Disease Endemic Zones: Implications for the recovery of banana production

PLOS One

Dear Dr. Omondi,

Thank you for submitting your manuscript to PLOS ONE. After careful consideration, we feel that it has merit but does not fully meet PLOS ONE’s publication criteria as it currently stands. Therefore, we invite you to submit a revised version of the manuscript that addresses the points raised during the review process.

Please submit your revised manuscript by Jul 25 2026 11:59PM. If you will need more time than this to complete your revisions, please reply to this message or contact the journal office at plosone@plos.org. When you're ready to submit your revision, log on to https://www.editorialmanager.com/pone/ and select the 'Submissions Needing Revision' folder to locate your manuscript file.

Please include the following items when submitting your revised manuscript:

• A letter that responds to each point raised by the academic editor and reviewer(s). You should upload this letter as a separate file labeled 'Response to Reviewers'.

• A marked-up copy of your manuscript that highlights changes made to the original version. You should upload this as a separate file labeled 'Revised Manuscript with Track Changes'.

• An unmarked version of your revised paper without tracked changes. You should upload this as a separate file labeled 'Manuscript'.

If you would like to make changes to your financial disclosure, please include your updated statement in your cover letter. Guidelines for resubmitting your figure files are available below the reviewer comments at the end of this letter.

If applicable, we recommend that you deposit your laboratory protocols in protocols.io to enhance the reproducibility of your results. Protocols.io assigns your protocol its own identifier (DOI) so that it can be cited independently in the future. For instructions see: https://journals.plos.org/plosone/s/submission-guidelines#loc-laboratory-protocols. Additionally, PLOS ONE offers an option for publishing peer-reviewed Lab Protocol articles, which describe protocols hosted on protocols.io. Read more information on sharing protocols at https://plos.org/protocols?utm_medium=editorial-email&utm_source=authorletters&utm_campaign=protocols.

As the corresponding author, your ORCID iD is verified in the submission system and will appear in the published article. PLOS supports the use of ORCID, and we encourage all coauthors to register for an ORCID iD and use it as well. Please encourage your coauthors to verify their ORCID iD within the submission system before final acceptance, as unverified ORCID iDs will not appear in the published article. Only the individual author can complete the verification step; PLOS staff cannot verify ORCID iDs on behalf of authors.

We look forward to receiving your revised manuscript.

Kind regards,

Sengottayan Senthil-Nathan, D.Sc

Academic Editor

PLOS One

Journal Requirements:

If the reviewer comments include a recommendation to cite specific previously published works, please review and evaluate these publications to determine whether they are relevant and should be cited. There is no requirement to cite these works unless the editor has indicated otherwise.

Please review your reference list to ensure that it is complete and correct. If you have cited papers that have been retracted, please include the rationale for doing so in the manuscript text, or remove these references and replace them with relevant current references. Any changes to the reference list should be mentioned in the rebuttal letter that accompanies your revised manuscript. If you need to cite a retracted article, indicate the article’s retracted status in the References list and also include a citation and full reference for the retraction notice.

Response: We appreciate and accommodate this comment in our revision

Action: References have been check and format aligned.

Additional Editor Comments:

Comments

1. Causality vs. Association: Clarify Interpretation

The abstract and discussion repeatedly use causal language (“driving”, “positively associate with”, “influencing infection”), but the study design is cross-sectional and observational. While multivariate modeling (e.g., GLM or random forest) likely underpins the reported associations, the manuscript does not explicitly state:

•Which statistical models were used (and justification for selection),

•Whether spatial autocorrelation was tested/accounted for (gardens may be clustered geographically, violating independence assumptions),

•Whether confounding (e.g., soil type, irrigation practices, proximity to infected fallow fields) was assessed or adjusted for.

Suggestion: Revise language to consistently reflect associations, not causation (e.g., “were associated with higher BBTD prevalence”). Add a brief methods subsection on model specification, diagnostics (VIF, residuals, spatial tests), and handling of potential confounders.

Response: The soil type was not considered in this study. While soil fertility could affect plant growth and eventual aphid density or disease trajectory, it was simply not the focus of the study.

Infected fallow fields: We believe the reviewer wishes to say fields without regular crop but which have diseased banana plants (?). All fields are considered with the presence of banana or not as a key indicator of banana density, given that the banana aphid and the BBTV do not have known alternative hosts. Aroids could have been an important crop to consider in a more controlled future study, as they have been reported to host Pentalonia spp.

Spatial tests are added in the Supplementary Figures following earlier recommendations. We are happy to move them back if this works.

Irrigation practices: There was no irrigation. Differences in cropping management could include intercropping, and perhaps soil fertility management. As banana is grown as a perennial, these factors are not considered in our model, we are happy to discuss them.

Action: The entire manuscript has been revised to remove implications of causality and put forth association as the key line of argument.

Model selection: This is an important comment and is accommodated in the manuscript. We describe the data summary and exploration to identify the most appropriate models given the data structure. Indeed we further evaluate the spatial autocorrelation as a way to test the support of our conclusions (Supplemetary Figure 1). See Lines 228 – 238.

2. The aphid quantification method lacks detail

The manuscript states “increased aphid populations” correlate with BBTD, yet it does not specify:

•How aphids were sampled (visual counts? yellow traps? leaf inspections?),

•Timing and frequency (single snapshot vs. seasonal monitoring),

•Whether Pentalonia nigronervosa (the sole BBTV vector) was confirmed morphologically or molecularly, or whether non-vector aphids were inadvertently included.

Suggestion: Clarify sampling protocol in Methods (ideally with a citation or standard protocol reference, e.g., IITA or FAO guidelines). If P. nigronervosa identification wasn’t verified, explicitly acknowledge this as a limitation.

Identification wasn’t verified; explicitly acknowledge this as a limitation.

Response: We agree with the reviewer on the need to add some possible limitation, which has been duly added. We however point out that due to vector host specificity, most colony-forming aphids on banana will be banana aphids (Pentalonia nigronervosa, or rarely P. caladii) (See Foottit 2010). Indeed aphids collected by us in multiple countries (Uganda, DRC, Burundi – same gardens as this study; Rwanda, Benin and Malawi) targeting multiple Musaceae hosts showed than a very small sample of banana aphids only from DRC were P caladii, the rest being P. nigronervosa. No other aphid species was recovered forming colonies on banana (See Wessels et al., 2019).

Action: We add a paragraph on aphid sampling (lines 186-200) with references. The limitation of potential misidentification of aphids is addressed in the discussion (lines 650 – 675) alongside other potential limitations

3. Rogueing definition and fidelity are ambiguous

“Frequent rogueing” is cited as protective, but no definition is provided:

•Was it self-reported by farmers? Observed? Documented in logs?

•What constituted “rogueing” — removal of symptomatic plants only? Or also asymptomatic neighbors (a recommended practice)?

•Was timing (e.g., pre- or post-symptom onset) recorded?

Suggestion: Define rogueing operationally in Methods and discuss the reliability of measurement (e.g., inter-farmer variability, recall bias). Consider adding a brief validation note if field staff verified reports.

Response: Roguing of asymptomatic neighbours is not really presently recommended, as BBTD does not spread in a diffusion model from a central plant. We have used the approach we previously tested (see Omondi et al., 2020) in Malawi, Burundi (this community) and Benin, involving regular roguing of diseases plants only. Some asymptomatic plants could be missed, at the tune of 2% (see Omondi et al., 2020). Where monthly roguing is done, asymptomatic plants were not significant subsequent infection sources (Chabi et al., 2023) and would be removed in subsequent assessment. Thus, only symptomatic plants were removed.

Asymptomatic mats close to the diseased plant were not removed (See Omondi et al., 2020; and 2026 – which tested this approach). This is not the recommended practice where monitoring is continuous, and is particularly repulsive to smallholder farmers due to the potential heavy loss of plants. Out later studies (Chabi et al., 2023) did show, in three cultivars, that aphids were not as successful in transmitting the virus from asymptomatic exposed plants to healthy ones. Spatial assessment in Australia (John Thomas, personal communication) since 2019 are consistent with this assessment. So only backyard bananas are treated like this given that they are not subjected to frequent/ consistent monitoring. We agree with the reviewer that farmer reported roguing without a parallel monthly technical log could be erroneous, but this kind of study was already done by us (Omondi et al., 2020) when we sought to evaluate the performance of roguing among smallholders. In this study, monthly reporting and three layer supervision (Farmer Group, Extension officer and Project Technician) was included. The bias of farmer reported roguing is addressed in the discussion and could explain why roguing does not come up strongly as associated with BBTD prevalence.

Action: We have now added a definition of the roguing protocol used and the reference in lines 206 – 2011 (corrected version with Track changes). That farmer-reported roguing was done is and added limitations associated with it in the text (lines the limitations of the approach is explained in the text) lines 211 – 214.

4. Lack of BBTV detection confirmation

The study assumes BBTD diagnosis = BBTV infection. However, visual symptoms can mimic other stresses (e.g., nematode damage, drought, nutrient deficiency). No mention is made of:

• Diagnostic confirmation (e.g., PCR, ELISA, or lateral flow assays) on a subsample,

• Inter-rater reliability of symptom scoring (if multiple enumerators were used).

Suggestion: State whether lab confirmation was performed — and if not, add a clear limitation acknowledging possible misclassification bias, which may attenuate or distort observed associations.

Response: This limitation has now been addressed. In an associated study, we had carried out ELISA and PCR tests (Burundi and Benin respectively) to verify infection. All symptomatic plants were positive in these studies.

While we agree that some symptoms of BBTD could resemble nutrient deficiency symptoms, especially Potassium; BBTD presents multiple symptoms that together are used to diagnose the disease symptomatically quite accurately. We have added this evaluation to the text,

Action: We have now added a definition of the roguing protocol used and the reference in lines 206 – 2011 (corrected version with Track changes). That farmer-reported roguing was done is and added limitations associated with it in the text (lines the limitations of the approach is explained in the text) lines 211 – 214.

Reviewer #1: Strengths

•Timely and applied relevance: BBTD remains a critical constraint to food security and livelihoods in smallholder banana systems across Africa and Asia. This study addresses a practical, underexplored question: why do virus-free plantings still fail?

•Strong field design: The assessment of 121 independently managed gardens — all established with certified virus-free plantlets — provides valuable real-world context beyond controlled trials.

•Ecologically grounded variables: Inclusion of landscape-level factors (elevation, hedgerows, varietal diversity, rogueing frequency) reflects a systems-thinking approach aligned with agroecological disease management principles.

•Clear translational messaging: Recommendations (e.g., barrier plants, mixed cropping, community rogueing protocols) are actionable and farmer-informed — highly appropriate for PLOS ONE’s mission of accessible, policy-relevant science.

Comments

1. Causality vs. Association: Clarify Interpretation

The abstract and discussion repeatedly use causal language (“driving”, “positively associate with”, “influencing infection”), but the study design is cross-sectional and observational. While multivariate modeling (e.g., GLM or random forest) likely underpins the reported associations, the manuscript does not explicitly state:

•Which statistical models were used (and justification for selection),

•Whether spatial autocorrelation was tested/accounted for (gardens may be clustered geographically, violating independence assumptions),

•Whether confounding (e.g., soil type, irrigation practices, proximity to infected fallow fields) was assessed or adjusted for.

Suggestion: Revise language to consistently reflect associations, not causation (e.g., “were associated with higher BBTD prevalence”). Add a brief methods subsection on model specification, diagnostics (VIF, residuals, spatial tests), and handling of potential confounders.

Response:

Response: The soil type was not considered in this study. While soil fertility could affect plant growth and eventual aphid density or disease trajectory, it was simply not the focus of the study.

Infected fallow fields: We believe the reviewer wishes to say fields without regular crop but which have diseased banana plants (?). All fields are considered with the presence of banana or not as a key indicator of banana density, given that the banana aphid and the BBTV do not have known alternative hosts. Aroids could have been an important crop to consider in a more controlled future study, as they have been reported to host Pentalonia spp.

Spatial tests are added in the Supplementary Figures following earlier recommendations. We are happy to move them back if this works.

Irrigation practices: There was no irrigation. Differences in cropping management could include intercropping, and perhaps soil fertility management. As banana is grown as a perennial, these factors are not considered in our model, we are happy to discuss them.

Action: The entire manuscript has been revised to remove implications of causality and put forth association as the key line of argument.

Model selection: This is an important comment and is accommodated in the manuscript. We describe the data summary and exploration to identify the most appropriate models given the data structure. Indeed we further evaluate the spatial autocorrelation as a way to test the support of our conclusions (Supplemetary Figure 1). See Lines 228 – 238.

2. The aphid quantification method lacks detail

The manuscript states “increased aphid populations” correlate with BBTD, yet it does not specify:

•How aphids were sampled (visual counts? yellow traps? leaf inspections?),

•Timing and frequency (single snapshot vs. seasonal monitoring),

•Whether Pentalonia nigronervosa (the sole BBTV vector) was confirmed morphologically or mole

Attachment Submitted filename: PLOSOne_ Response to Reviewers R2.docx

# Editor report: Decision Letter 3

**DOI:** 10.1371/journal.pone.0357834.r007

**Authors:** Sengottayan Senthil-Nathan

23 Aug 2026

Drivers of Banana Re-infection in Newly Planted Gardens in Banana Bunchy top Disease Endemic Zones: Implications for the recovery of banana production

PONE-D-25-42829R3

Dear Dr. Omondi,

We’re pleased to inform you that your manuscript has been judged scientifically suitable for publication and will be formally accepted for publication once it meets all outstanding technical requirements.

Within one week, you’ll receive an e-mail detailing the required amendments. When these have been addressed, you’ll receive a formal acceptance letter and your manuscript will be scheduled for publication.

An invoice will be generated when your article is formally accepted. Please note, if your institution has a publishing partnership with PLOS and your article meets the relevant criteria, all or part of your publication costs will be covered. Please make sure your user information is up-to-date by logging into Editorial Manager at Editorial Manager® and clicking the ‘Update My Information' link at the top of the page. For questions related to billing, please contact billing support.

If your institution or institutions have a press office, please notify them about your upcoming paper to help maximize its impact. If they’ll be preparing press materials, please inform our press team as soon as possible -- no later than 48 hours after receiving the formal acceptance. Your manuscript will remain under strict press embargo until 2 pm Eastern Time on the date of publication. For more information, please contact onepress@plos.org.

Kind regards,

Sengottayan Senthil-Nathan, D.Sc

Academic Editor

PLOS One

Additional Editor Comments (optional):

Reviewers' comments:

# Editor report: Acceptance letter

**DOI:** 10.1371/journal.pone.0357834.r008

**Authors:** Sengottayan Senthil-Nathan

PONE-D-25-42829R3

PLOS One

Dear Dr. Omondi,

I'm pleased to inform you that your manuscript has been deemed suitable for publication in PLOS One. Congratulations! Your manuscript is now being handed over to our production team.

At this stage, our production department will prepare your paper for publication. This includes ensuring the following:

* All references, tables, and figures are properly cited

* All relevant supporting information is included in the manuscript submission,

* There are no issues that prevent the paper from being properly typeset

You will receive further instructions from the production team, including instructions on how to review your proof when it is ready. Please keep in mind that we are working through a large volume of accepted articles, so please give us a few days to review your paper and let you know the next and final steps.

Lastly, if your institution or institutions have a press office, please let them know about your upcoming paper now to help maximize its impact. If they'll be preparing press materials, please inform our press team within the next 48 hours. Your manuscript will remain under strict press embargo until 2 pm Eastern Time on the date of publication. For more information, please contact onepress@plos.org.

You will receive an invoice from PLOS for your publication fee after your manuscript has reached the completed accept phase. If you receive an email requesting payment before acceptance or for any other service, this may be a phishing scheme. Learn how to identify phishing emails and protect your accounts at https://explore.plos.org/phishing.

If we can help with anything else, please email us at customercare@plos.org.

Thank you for submitting your work to PLOS One and supporting open access.

Kind regards,

PLOS One Editorial Office Staff

on behalf of

Prof. Sengottayan Senthil-Nathan

Academic Editor

PLOS One
